# Vistas in the domain of 3-acetyl-4-hydroxy-2-quinolinone derivatives (AHQ) and their applications

**DOI:** 10.1039/d5ra02813b

**Published:** 2025-06-04

**Authors:** Nedaa N. Elnaggar, Wafaa S. Hamama, Eslam A. Ghaith

**Affiliations:** a Chemistry Department, Faculty of Science, Mansoura University Mansoura 35516 Egypt wshamama@mans.edu.eg wshamama53@gmail.com +20-1093427024; b Chemistry Department, Faculty of Science, New Mansoura University New Mansoura City Egypt

## Abstract

This review discusses the significant advances in the current status and latest synthesis techniques for *N*-substituted 3-acetyl-4-hydroxyquinolinones. They are an important class of alkaloids possessing different electrophilic and nucleophilic centers. In this review, we comprehensively summarize the synthesis of 3-acetylquinolones *via* various reactions, emphasizing recent developments and challenges. Synthetic transformations of quinolone are focused on electrophilic substitution reactions supported by their mechanistic pathways, as well as nucleophilic substitution reactions and cycloaddition reactions, which have allowed access to an extensive scope of binary and fused heterocyclic scaffolds, including pyrazoles, imidazoles, tetrazoles, pyridines, pyrimidines, triazines, azepines, and pyranones. Also, the utilization of quinolone reactions as key intermediates in the ongoing and forthcoming marketed pharmaceutical syntheses is discussed. Additionally, this review covers various potential applications, including complexation, fluorescence sensing, chemical pH sensors, agricultural, and anticorrosion.

## Abstract 2

This review discusses the significant advances in the current status and latest synthesis techniques for *N*-substituted 3-acetyl-4-hydroxyquinolinones.

## Graphical abstract

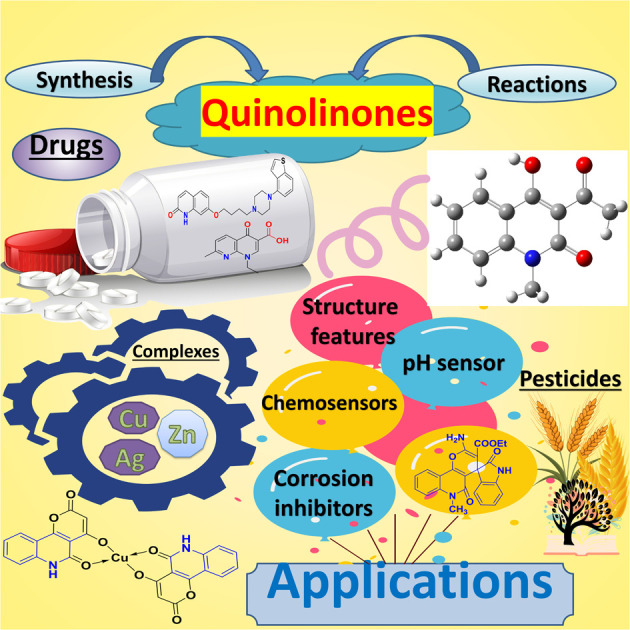

## 1. Introduction and scope

Heterocyclic skeletons which contain a nitrogen atom represent a common unit of a vast amount of marketed drugs,^1^ constituting the most extensive and diverse collection of natural, synthetic organic compounds, agrochemicals, and pharmaceuticals.^2–4^ Furthermore, the chemistry of heterocycles aggregates an essential branch of the field of drug design and the evolution of novel biologically active compounds,^5–11^ including antiparasitic,^12,13^ anti-HIV,^14,15^ antiepileptic drugs,^16,17^ analgesic,^18,19^ anthelmintic,^20,21^ inhibitors of platelet aggregation, anti-inflammatory,^22–25^ antibacterial and antiviral drugs,^26–29^ atherosclerosis,^30,31^ inhibitors for Parkinson's disease,^32^ antiproliferative, pulmonary antifibrotic agents^33^ and antitumor.^34–36^ Additionally, they provide one of the most fruitful sources for drug discovery and development, owing to synthesizing various scaffolds *via* robust synthetic approaches.^37–40^ These are important in synthesizing dyes, pigments, and polymeric materials^41–43^ due to their optical and fluorescence properties.^44^ The ongoing research in synthetic organic chemistry is dedicated to advancing these goals and addressing the demand for improved and sustainable approaches in synthesizing aromatic heterocyclic compounds.^2–4^ Easily accessible nitrogenous scaffolds are fundamental feedstocks; among these, quinoline and quinolones have piqued the interest of scientists since the 19th century, as over 600 quinoline derivatives have been extracted from natural sources to date.

Quinolinones have been perceived for their fascinating structural notions and versatile applications.^45–56^ Also, the quinolinones structures are pivotal building blocks,^46^ as they are commonly conspicuous scaffolds in a wide range of pharmacologically active synthetic and natural compounds.^57–68^ Quinolin-2-one, also referred to as benzo[*b*]pyridine, and 1-azanaphthalene, is important classes of natural product as *N*-based heterocycles which extracted from *Toddalia asiatica* leaves and plants, marine organisms and microorganisms which containing the quinolinone derivatives alkaloid findersine,^52,69–75^ consists of a benzene ring fused with a pyridine ring, sharing two carbon atoms between them and nitrogen atom not present as ring junction atom.

**Fig. 1:**
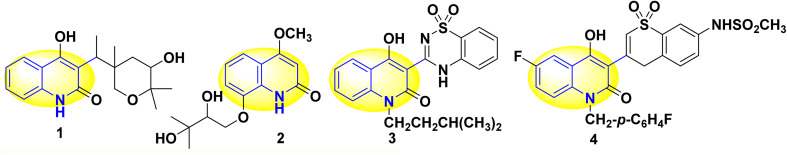
Some natural alkaloids quinolinone-based molecules.

Quinoline is a weak base capable of forming salts with acids. It reacts similarly to pyridine and benzene and undergoes either nucleophilic or electrophilic substitution processes.^47,76–81^ The importance of the quinoline moiety is being a vital component in many naturally occurring heterocyclic and medicinal plant families. In addition, bucharidine 1 and foliosidine 2 are quinoline alkaloids that are extracted from *Haplophyllum foliosum* and *Haplophyllum bucharicum*, respectively (Fig. 1). Also, both of them have viral-RNA polymerase inhibitory action and estrogenic action, such as compounds 3 and 4, which strongly inhibit the replication of the hepacivirus C.^82–84^

**Fig. 2:**
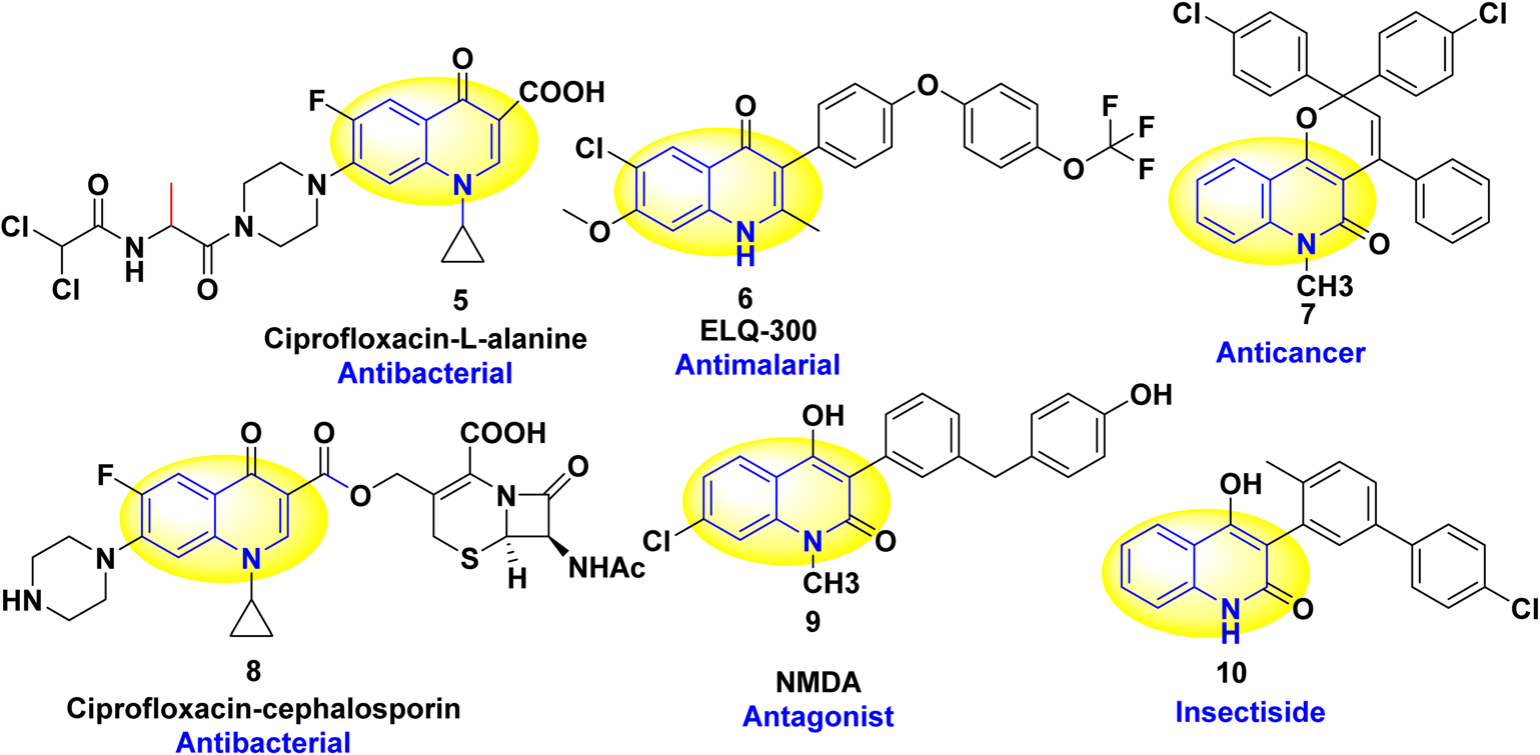
Chemical structure of biologically active quinolinone scaffolds.

Additionally, quinolines have various potential biological and pharmacological activities including antimalarial,^84–86^ antibacterial,^87^ antifungal,^88,89^ anticancer,^90–92^ anti-HIV (Human Immunodeficiency Virus),^93^ antiviral,^94^ antitumor,^95–97^ anthelmintic,^98^ antioxidant,^49^ cardiotonic,^76,99–101^ anticonvulsant,^102,103^ anti-inflammatory and analgesic properties^76,77,104,105^ (Fig. 2).

## 2. Chemical reactivity

### 2.1. 3-Acetyl-*N*-substituted/(un)-4-hydroxy-quinolin-2(1*H*)one derivatives (AHQ)

AHQ is the basic ring structure of numerous alkaloids and is a highly versatile scaffold with an extensive range of applications. Progress in synthetic technologies is leading to the production of various functionalized quinolinone derivatives as biosteric analogues to linomide (4-hydroxyquinolinone) derivatives.^82,106–112^

### 2.2. Structural feature of AHQ

#### 2.2.1. Tautomeric structures

Whereas, the *β,β*′-tricarbonyl (TC) group in AHQ compound provides an analogue of 3-acyl pyrrolidine-2,4-diones (tetramic acid), and has suitable sites for interaction with different nucleophilic reagents, with its regioselectivity of TC for AHQ [(A)–(D)] given below in a framework of molecular orbital perturbation.^113^

**Fig. 3:**
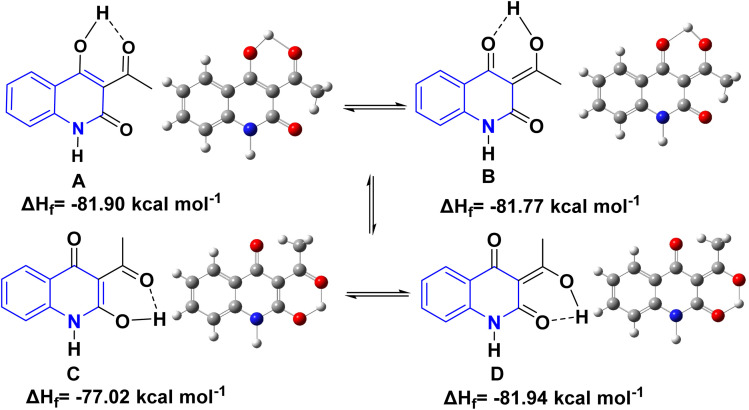
Tautomeric form of AHQ 12a and its geometrical structures.

Detsi and coworkers^114^ predicting the four tautomeric structures A–D. of AHQ [R=H] 12a (Fig. 3). The tautomeric equilibrium showed the presence of tautomeric forms that could be in one of four possible structures, A–D (Fig. 3). Whereas, semiempirical quantum calculations showed that the tautomer D was the most stable favorable one according to the total energy values.^114,115^ Whereas, the constitutions of AHQ derivatives were fully elucidated *via* different spectroscopic analyses as shown in Table 1.^114,116–118^

**Table 1:** Spectral data of AHQ 12a–c

Spectroscopic technique	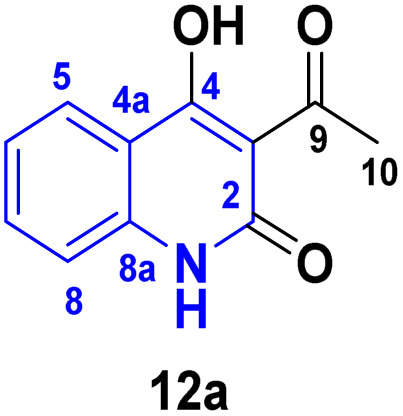	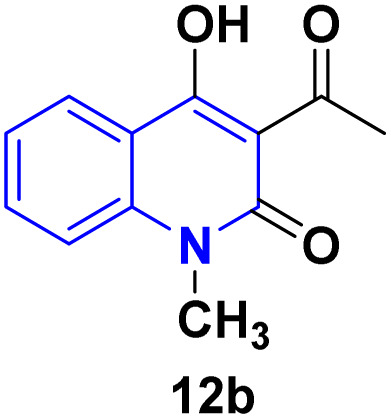	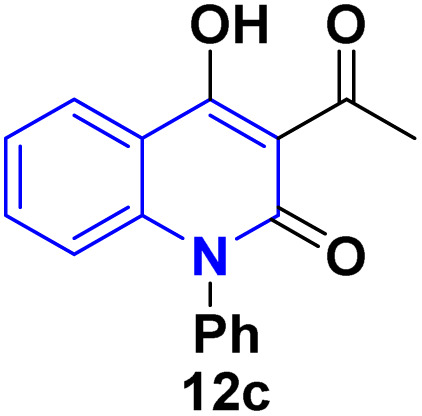
^1^H NMR (DMSO-*d*_6_)	2.72 (s, 3H, COCH_3_), 7.23 (t, 1H, H-6), 7.30 (d, 1H, H-8, *J* = 8.3 Hz), 7.65 (t, 1H, H-7), 7.99 (dd, 1H, H-5, *J* = 8.0, *J* = 1.2 Hz), 11.53 (s, 1H, NH) and 17.04 (s, 1H, OH)^116^	2.79 (s, 3H, COCH_3_), 3.52 (s, 3H, N–CH_3_), 7.30 (t, 1H, H-6), 7.50 (d, 1H, H-8, *J* = 8.1 Hz), 7.78 (t, 1H, H-7), 7.96 (dd, 1H, H-5, *J* = 8.4 Hz, *J* = 1.5 Hz) and 17.04 (s, 1H, OH)^116^	2.77 (3H, s, 3-COCH_3_), 7.12–7.41 (9H_Arom_), 8.23 (s, OH)^117^
			Our work
			2.79 (s, 3H, COCH_3_, 6.58 (d, 1H, *J* = 9 Hz), 7.23 (m, 1H), 7.27 (d, 2H, *J* = 8 Hz), 7.45 (t, 1H, *J* = 8 Hz), 7.53 (t, 1H, *J* = 7 Hz), 7.61 (t, 2H, *J* = 8 Hz), 8.24 (d, 1H, *J* = 8 Hz), 17.16 (s, 1H, OH)
^13^C NMR (DMSO-*d*_*6*_)	205.7 (C-9), 174.7 (C-4), 161.1 (C-2), 140.5 (C-8a), 134.8 (C-7), 124.7 (C-5), 122.0 (C-6), 115.5 (C-8), 113.3 (C-4a), 105.7 (C-3) and 30.5 (C-10)^116^	206.7 (C-9), 173.3 (C-4), 160.6 (C-2), 141.6 (C-8a), 135.7 (C-7), 125.4 (C-5), 122.3 (C-6), 114.4 (C-8), 115.3 (C-4a), 105.7 (C-3), 31.3 (C-10) and 28.9 (N–CH_3_)^116^	206.9 (C-9), 175.0 (C-4), 161.9 (C-2), 142 (1C), 137.4 (1C), 134.4 (1C), 130.3 (2C), 129.0 (2C), 125.8 (1C), 122.3 (1C), 117.7 (1C), 116.0 (1C), 115.2 (1C), 106.0 (1C), 31.4 (1C)
IR (KBr, *v*_max/_cm^−1^)	3360 (OH), 3160 (NH), 1661 (C=O, acetyl), 1622 (C=O, amide), 1606 (C=C)^116^	3250 (OH), 1658 (C=O, acetyl), 1623 (C=O, amide 1598 (C=C)^117^	3072 (CH_Arom_), 2924 (CH_Aliph_), 1654(C=O, acetyl, enol form), 1618 (C=O, amide).^114,118^

#### 2.2.2. X-ray crystallographic

**Fig. 4:**
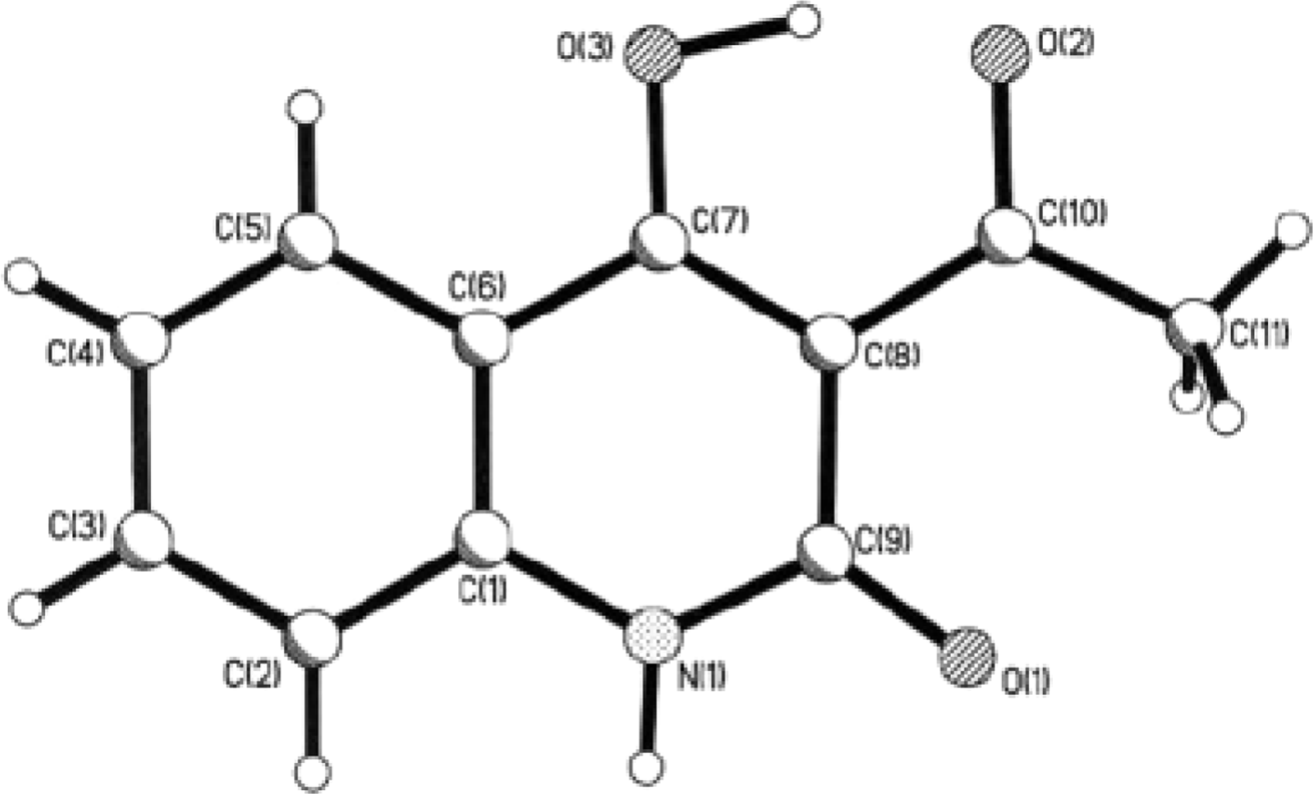
X-ray structural analysis 12a.^120^

The constitution of AHQ 12a was supported by X-ray analysis, as the non-hydrogen atoms in compound 12a are located in a single plane with an accuracy of 0.02 Å, this phenomena indicated the formation of an intramolecular hydrogen bond O(3)–H(3O)⋯O(2) (H⋯O 1.41 Å, O–H⋯O 157°). Also, this phenomenon displayed the re-estimation of the intramolecular electronic charge as shown by the bond lengths O(2)–C(10) 1.255(2) and C(7)–C(8) bonds 1.393(2) (Fig. 4).^119,120^

## 3. Aspects of the AHQ synthetic approach

**Fig. 5:**
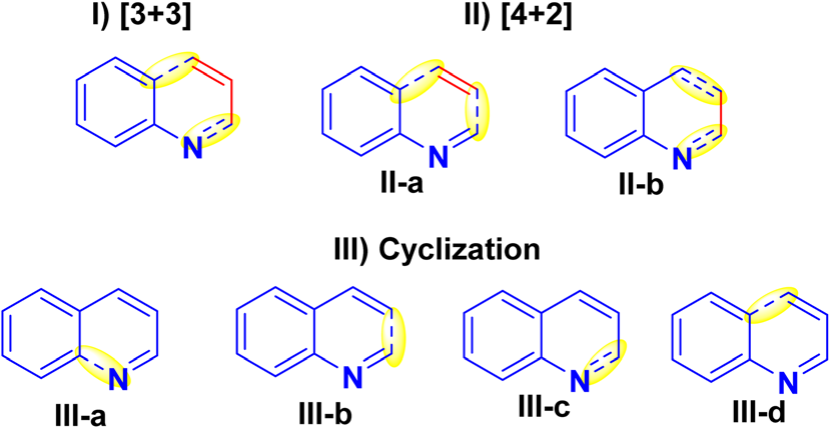
Common synthetic methods of quinolines.

Quinolines can be synthesized *via* different synthetic strategies involving [3 + 3], [4 + 2] and cyclization methodologies (I–III).^121^

(I) [3 + 3] Annulation: This strategy includes established procedures, for instance, the Skraup, Conrad-Limpach, Doebner-von Miller, Combes, and Gould-Jacobs syntheses. However, the drawback of these techniques is that they are restricted in regioselectivity for the synthesis of multi-substituted quinolines.^121–123^

(II) [4 + 2] Annulation: There are two techniques to build pyridine nuclei annulated to the benzene ring as shown in designed forms (II-a and II-b), as the first one has minimal regioselectivity with a narrow substrate range.^121,124,125^ While the other method is more prevalent and employs easily available substrates to produce products with high regioselectivity as Ptzinger and Friedlander reactions.^121,122,126,127^

(III) Cyclization: There are four distinct ways to generate the pyridine ring of quinolines (III-a to III-d) using cyclization. However, they have not been achieved due to the complicated reaction methodology and the limited availability of the starting substrates (Fig. 5).^128,129^

Synthetic approaches to construct AHQ derivatives have been achieved *via* many routes: aniline, alkaline hydrolysis of pyranoquinoline, anthranilic acid derivatives, heterocyclization of 3,1-benzoxazine-4-one and furo[3,2-*c*]quinoline.

### 3.1. Synthesis from amines/pyrano[3,2-*c*]quinolindione

**Scheme 1:**
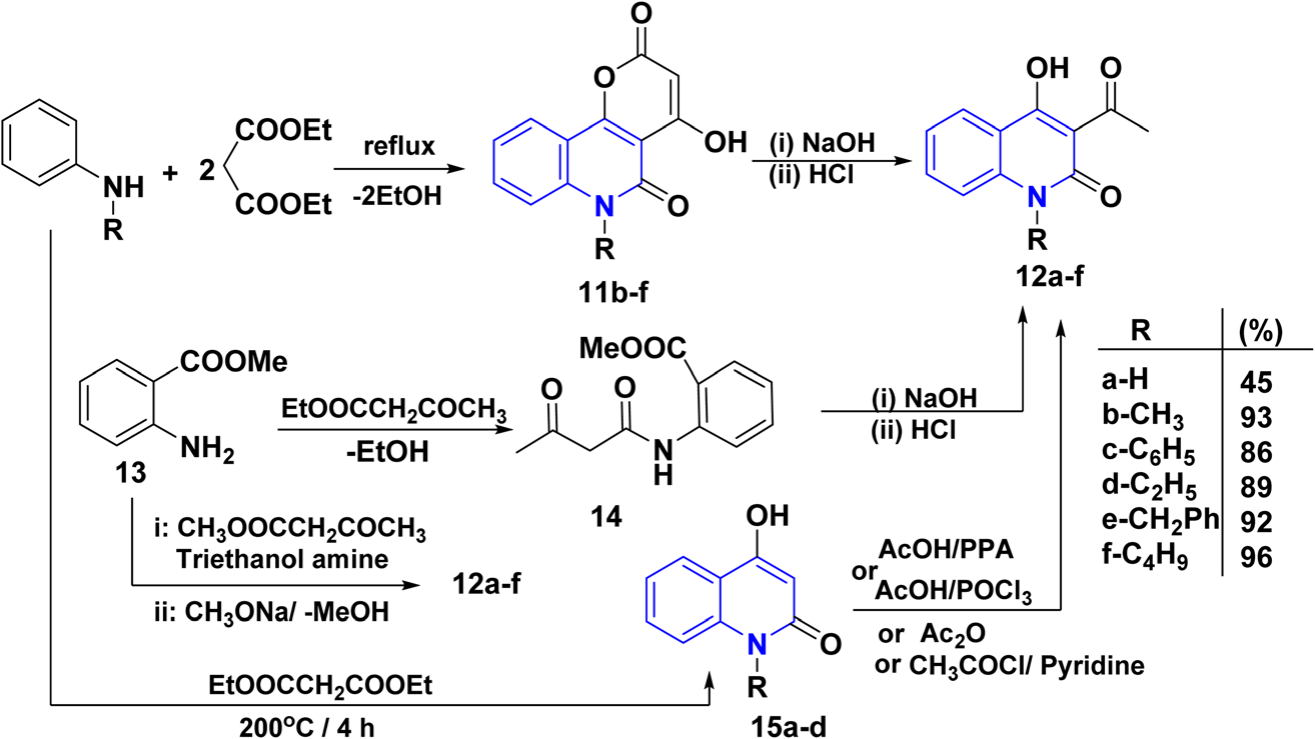
Synthesis of *AHQ*12a–f.

Quinoline derivatives 12a–f were prepared by cyclocondensation of substituted amines and diethyl malonate in a molar ratio of 1 : 2 for 3–6 h. Till the calculated amount of ethanol was collected using Dean Stark by a short Vigreux column as distillation system afforded the lactone 11, followed by alkaline hydrolysis [NaOH (2 N)], of lactone 11 and subsequent decarboxylation of the intermediate *β*-oxocarboxylate then acidified with HCl (2 N) to yield the products 12a–f in high yields (86–96%). In the case of 12b, compound 11 was synthesized following the methods of Kappe and Stadlbauer, which involved reacting *N*-methylaniline with diethyl malonate in diphenyl ether. However, Kappe's method utilized an excess of diethyl malonate, which served a dual purpose as both a reagent and a solvent (Scheme 1).^115,130–135^ Also, the ring opening of 11b–f by NaOH and subsequent spontaneous decarboxylation afforded 4-hydroxyquinolinone derivatives 12b–f (Scheme 1).^112,133,135–137^

### 3.2. Synthesis from 4-hydroxyquinolin-2-ones

Cyclocondensation reaction of secondary amine and diethyl malonate afforded 4-hydroxyquinolin-2-ones 15a–d. After that, acetylation reaction of *N*-unsubstituted AHQ 15a–d with acetyl chloride using acetic acid or pyridine in the presence of polyphosphoric acid (PPA) or AcOH with phosphorus oxychloride (POCl_3_) yielded acetylquinoline derivatives 12a–d (Scheme 1).^135–140^

### 3.3. Synthesis from methyl 2-aminobenzoate/*o*-aminobenzaldehydes/*o*-aminoarylketones

Synthesis of acetylquinoline 12a was accomplished *via* acylating methyl anthranilate 13 using ethyl acetoacetate or methyl acetoacetate and triethanolamine by continuous removal of methanol or ethanol using Dean–Stark apparatus, resulting in the formation of 2-methoxycarbonyl anilide 13, followed by Dieckmann intramolecular cyclization of anilide derivative 14 (Scheme 1).^120,141–143^

### 3.4. Synthesis from 2-methyl-3,1-benzoxazin-4-one

**Scheme 2:**
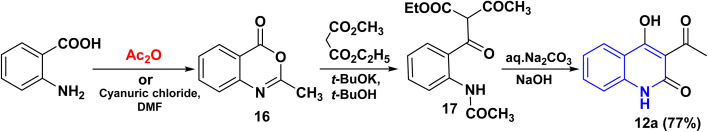
Synthesis of 12a from anthranilic acid.

The synthesis of 12a was performed through a two-step. First, 2-methyl-3,1-benzoxazin-4-one 16 was synthesized by [4 + 2] annulation of anthranilic acid or by using the iminium cation from a mixture of cyanuric chloride and dimethylformamide as a cyclizing agent, and was achieved under mild conditions.^144^ Next, C-acylated with ethyl acetoacetate by 3,1-benzoxazin-4-one 16 to form the ester 17. Finally, cyclization of ester 17 in a basic medium [aqueous Na_2_CO_3_/NaOH] at room temperature furnished the target compound 12a. This reaction pathway involves the nucleophilic addition of the active methylene of ethyl acetoacetate (EAA) to the carbonyl group of the benzoxazine ring 16, followed by intramolecular cyclization (Scheme 2).^108,116,145–147^

**Scheme 3:**
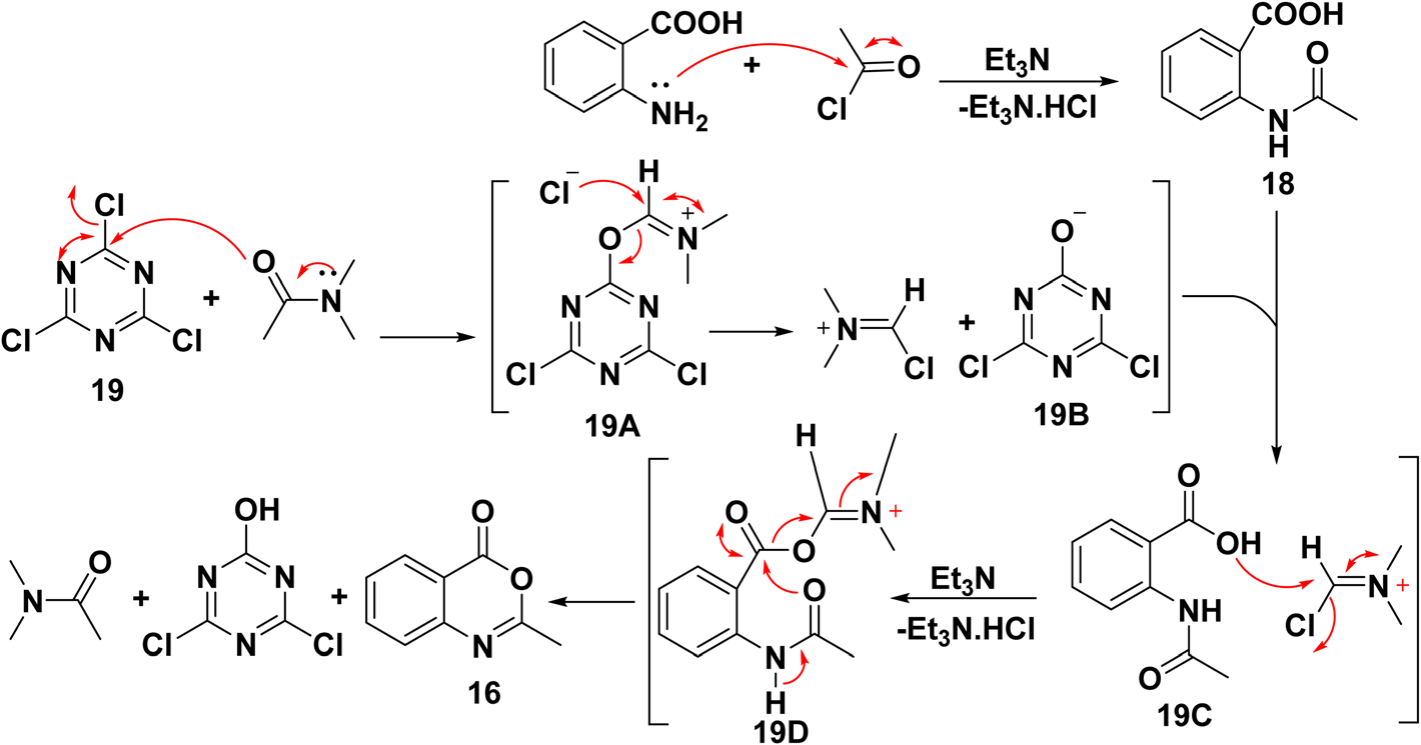
Suggested mechanism for the synthesis of benzoxazinone derivatives 16.

*N*-Acylated anthranilic acid intermediate 18 is created from anthranilic acid and acid chloride in the presence of a catalytic amount of triethylamine (TEA) as the HCl scavenger *via* N-acylation reaction, affording *N*-acylated anthranilic acid. After that, intramolecular nucleophilic attack at intermediate 18 yielded benzoxazin-4-one ring 16. The cyclization reaction can be achieved by converting the carboxylic group of acetylated derivative 18 into a dynamic ester through either microwave irradiation or traditional heating (Scheme 3).^144^

### 3.5. Synthesis from 1,3-dioxinone with methyl 2-(methylamino)benzoate

**Scheme 4:**
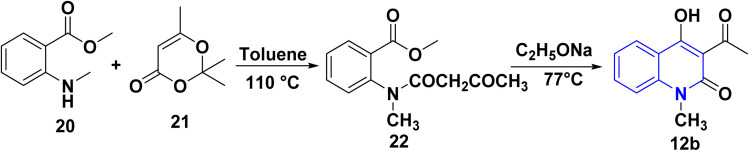
Synthesis of 12b through acylation of 20 followed by cyclization of compound 22.

The synthesis of 12b was accomplished *via* an acylation reaction of secondary amine 20 with trimethyl-1,3-dioxinone 21, followed by a cyclization reaction of the acylation product 22 under basic conditions (Scheme 4).^108^

### 3.6. Synthesis from angular furo[3,2-*c*]quinolinone derivatives

**Scheme 5:**
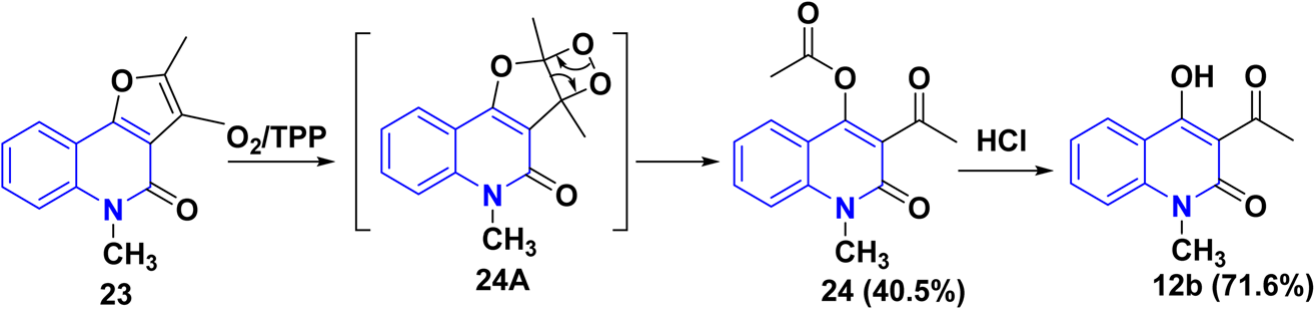
Photooxygenation reaction of 23.

In 2021, the group of Elgogary described the photooxygenation reaction of furo [3,2-*c*] quinolin-4(5*H*)-one derivatives 23 in CHCl_3_ led to the formation of the photocleaved product 24*via* the intermediate 24A. Next, acid hydrolysis of quinolinone derivative 24 furnished 12b (Scheme 5).^139^

### 3.7. Synthesis from ketene dithioacetal

**Scheme 6:**
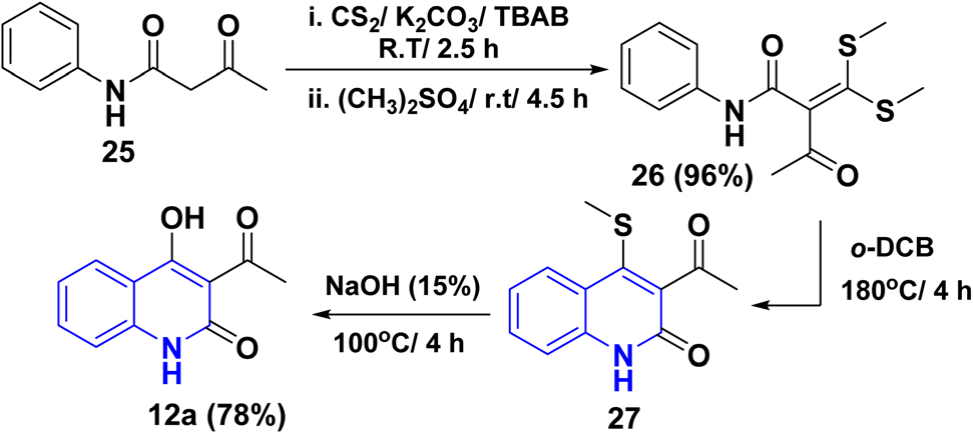
Synthesis of 12a from acetoacetanilide.

Compound 12a was synthesized through *in situ* direct addition of carbon disulfide (CS_2_) to acetoacetanilide 25 in the presence of K_2_CO_3_ and tetrabutylammonium bromide (TBAB) as a green ionic liquid. The alkylation process was achieved using dimethyl sulfate, which yielded the ketene dithioacetal 26. In the same context, thermal cyclization of ketene 26 afforded the quinolinone 27 in fair yield. Finally, basic hydrolysis of the quinolone 27 furnished 3-acetyl-4-hydroxyquinolinone 12a (Scheme 6).^140^

### 3.8. Synthesis of dehydroxylated-3-acetylquinoline (AQ)

**Scheme 7:**
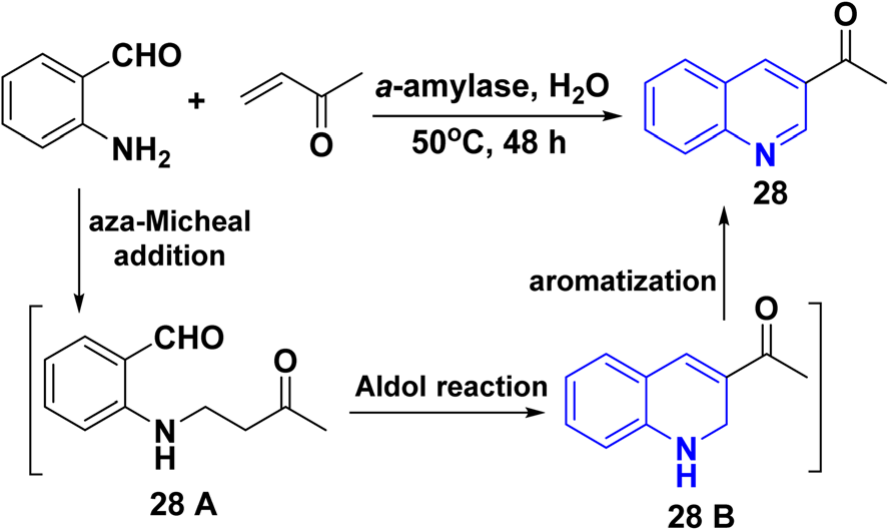
Synthesis of dehydroxylated-3-acetylquinoline (AQ) 28.

Also, synthesis of dehydroxylated-3-acetylquinoline (AQ) 28*via* the reaction of 2-aminobenzaldehydes with *α*,*β*-unsaturated carbonyl compounds as methyl vinyl ketone in high catalytic efficiency (56–86% yield), through sequence process as aza-Michael reaction gave 28 A followed by Aldol reaction afforded 28 B then aromatized in presence of α-amylase catalyzed (Scheme 7).^148^

### 3.9. Synthesis of 2-acetylquinoline derivatives

**Scheme 8:**
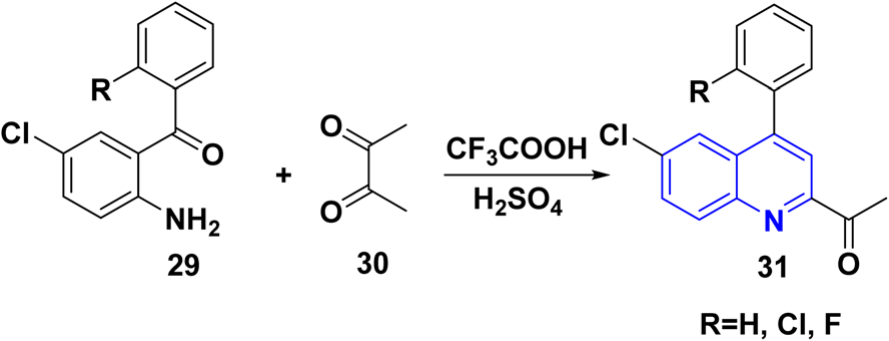
Synthesis of 2-acetylquinoline 31.

**Scheme 9:**
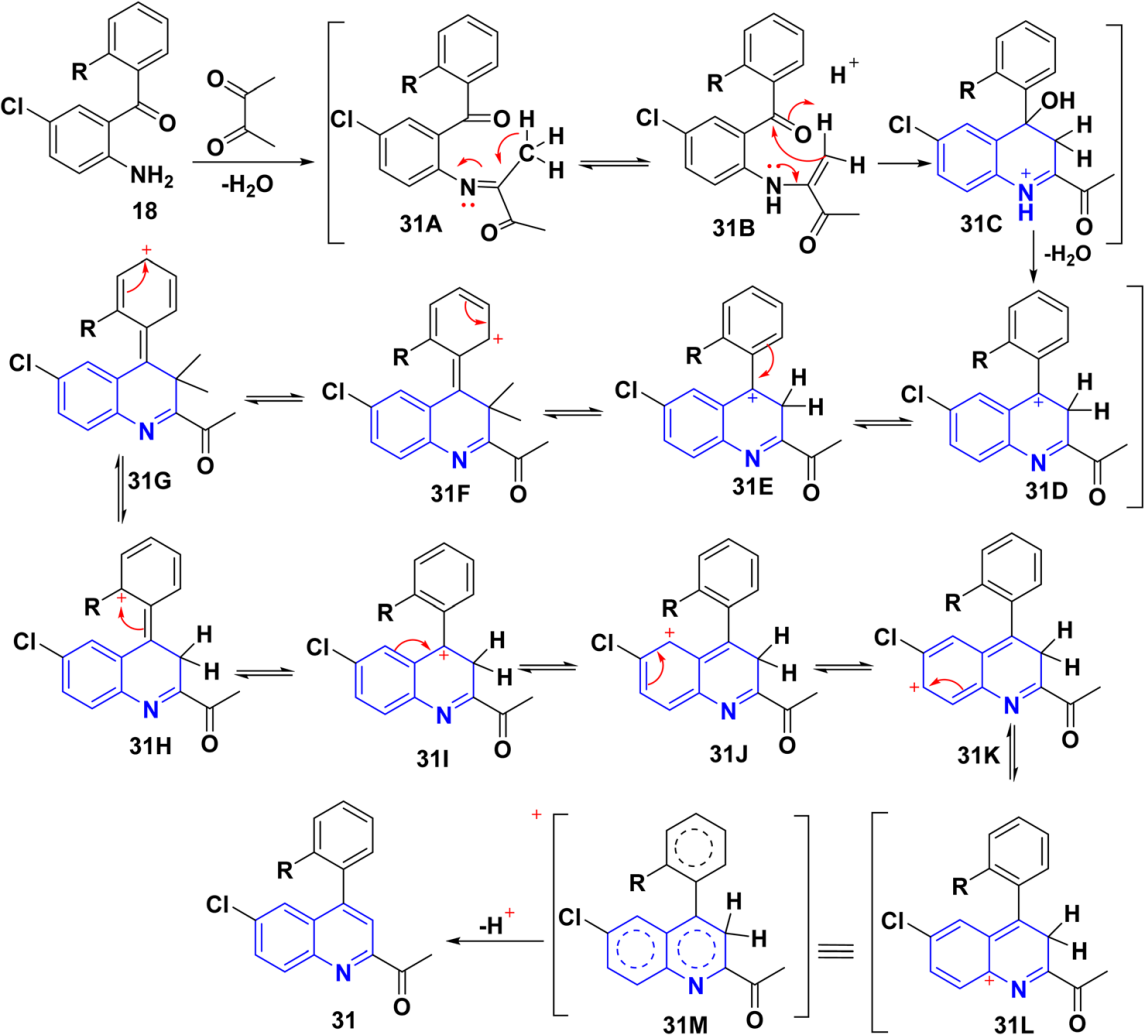
Proposed mechanism for synthesis of 2-acetylquinoline 31.

The reaction of *o*-aminoarylketones 29 with butanedione as a symmetrical 1,2-diketone 30 according to Friedländer synthesis. Similarly, *o*-aminoarylketones condensed with pentanedione (unsymmetrical 1,2-diketone) under acidic conditions led to the formation of regioselective 2-acetylquinoline derivatives 31 (Scheme 8). The suggested reaction mechanism (Scheme 9) is analyzed using density functional theory calculations at the B3LYP/6-311G (d,p) level. It was determined that all relative energy barriers and activation energies for the reaction steps are minimized at these theoretical levels.^149^

A plausible mechanism for forming product 31 is shown in Scheme 9. Initially, *o*-aminoaryl ketone (29) condenses with butan-2,3-dione (30) in the presence of TFA with conc. H_2_SO_4_ to give an imine intermediate 31A as geometrical isomer E by eliminating H_2_O. Then, tautomerization of the 31A into a more activated form, the enamine intermediate 31B. Followed by nucleophilic attack of *β*-position of enamino group on the carbonyl carbon and this gives the benzylic carbocation (which stabilise through eight resonance hybrid structures 31(G-H) and subsequently loses a proton to form final product subsequently loses a proton *via* aromatization to form final product 31.

### 3.10. Synthesis of quinolinones

**Scheme 10:**
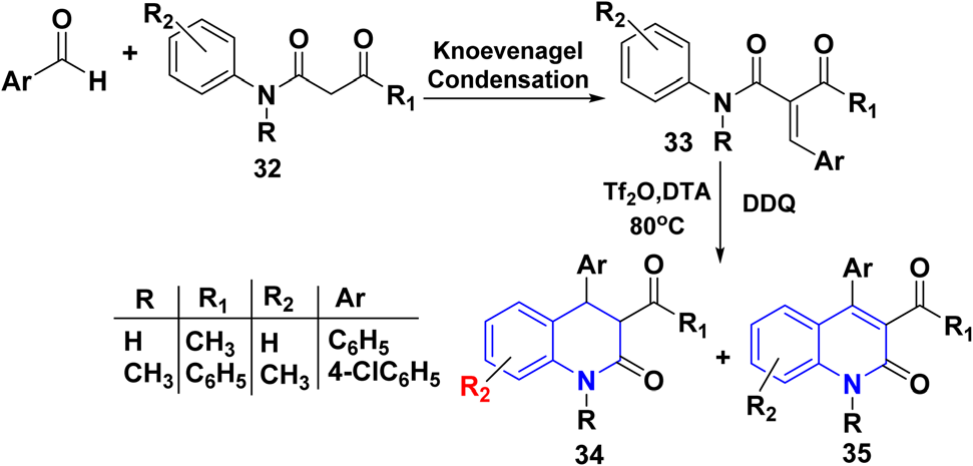
Triflic anhydride-mediated synthesis of quinolinone.

Zhang *et al.*^150^ reported an efficient methodology for preparing of 3,4-dihydroquinolinone 34 and quinolinone derivatives 35 through intramolecular cyclization of *N*-aryl cinnamides 33. Compound 33 was synthesized through Knoevenagel condensation of *β*-oxo-amides 32 with various aryl aldehydes, which were then subjected to intramolecular cyclization. The optimized conditions for cyclization, including solvent, the reaction temperature, and various catalysts such as Tf_2_O and DDQ, were investigated, whereas the optimum temperature is 80 °C, and *N*,*N*-dimethyl trifluoroacetamide (DTA) as solvent afforded the most satisfactory results. Under the optimised conditions, the reaction of a wide range of substrates such as *α*-acetyl-*N*-aryl cinnamides and *α*-benzoyl *N*-aryl secondary cinnamides proceeded efficiently to afford the corresponding quinoline-2(1*H*)-ones 34 and 35 (Scheme 10).^150^

## 4. Reactivity of AHQ

According to the current literature survey, the reactivity of AHQ 12a–f is part of scientific research with many reagents, as illustrated below. AHQ is a quinolin-2-one derivative with a unique chemical structure consisting of a *β*,*β*′-tricarbonyl (TC) group and thus a quinolinone ring with a carbonyl (acetyl), hydroxyl (enolate), and carbonyl (lactamic) group attached to it.

### 4.1. Electrophilic substitution reactions

An electrophilic substitution reaction is a chemical reaction in which an electrophile replaces a functional group attached to a compound, typically displacing a hydrogen atom. These reactions usually follow a three-step mechanism that includes forming an electrophile, forming a carbocation (an intermediate), and removing a proton from this intermediate. AHQ derivatives can efficiently undergo electrophilic substitution reactions such as alkylation, halogenation, Friedel–Crafts, and formylation.^151^

#### 4.1.1. Alkylation

##### 4.1.1.1. *N*_1_-Alkylation

**Scheme 11:**
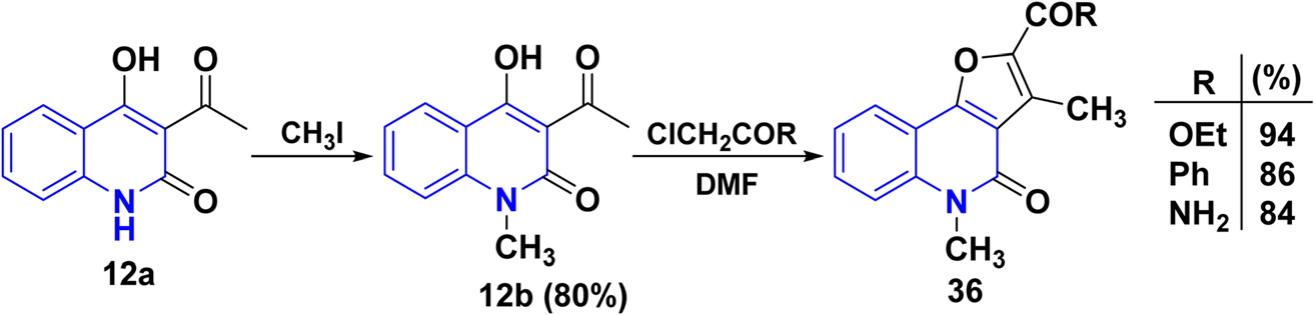
Formation of angular *N*-methylfuro[3,2-*c*]quinolinones 36.

Compound 12a can potentially undergo methylation at either the nitrogen atoms at positions 1 or the oxygen atom at C_4_. However, the methylation exclusively occurs at the nitrogen atom rather than the oxygen atom.^115,116,143,145^ Following that, angular *N*-methylfuro[3,2-*c*]quinolinone derivatives 36 were synthesized from 12b through the Rap–Stöermer reaction, with *α*-chlorocarbonyl substrates in the presence of K_2_CO_3_ in DMF (Scheme 11).

**Scheme 12:**
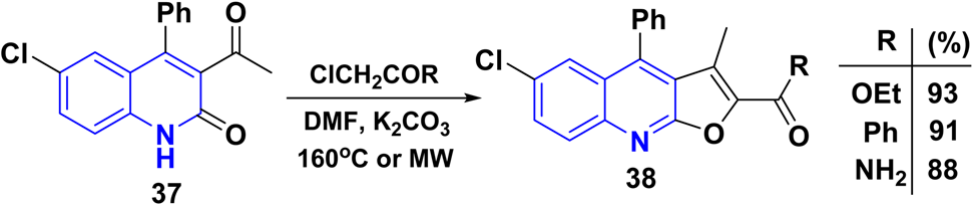
Formation of linear furo[2,3-*b*]quinolinones 38.

Whereas, the reaction of 3-acetyl-6-chloro-4-phenylquinolinone 37 with different *α*-halocarbonyl compounds afforded linear tetrasubstituted furo[2,3-*b*]quinolines 38 (Scheme 12). Thus, angular fluoroquinolinones 36 have been synthesized *via* the reaction of 12b derivatives with *α*-chlorocarbonyl compounds, as applied to the Rap–Stöermer reaction through both conventional and/or microwave methods. Thus, the microwave irradiation provided higher yields (88–93%) compared to the conventional method (64–72%) (Scheme 12).^143,152,153^

##### 4.1.1.2. *O*-Alkylation and its transformation

**Scheme 13:**
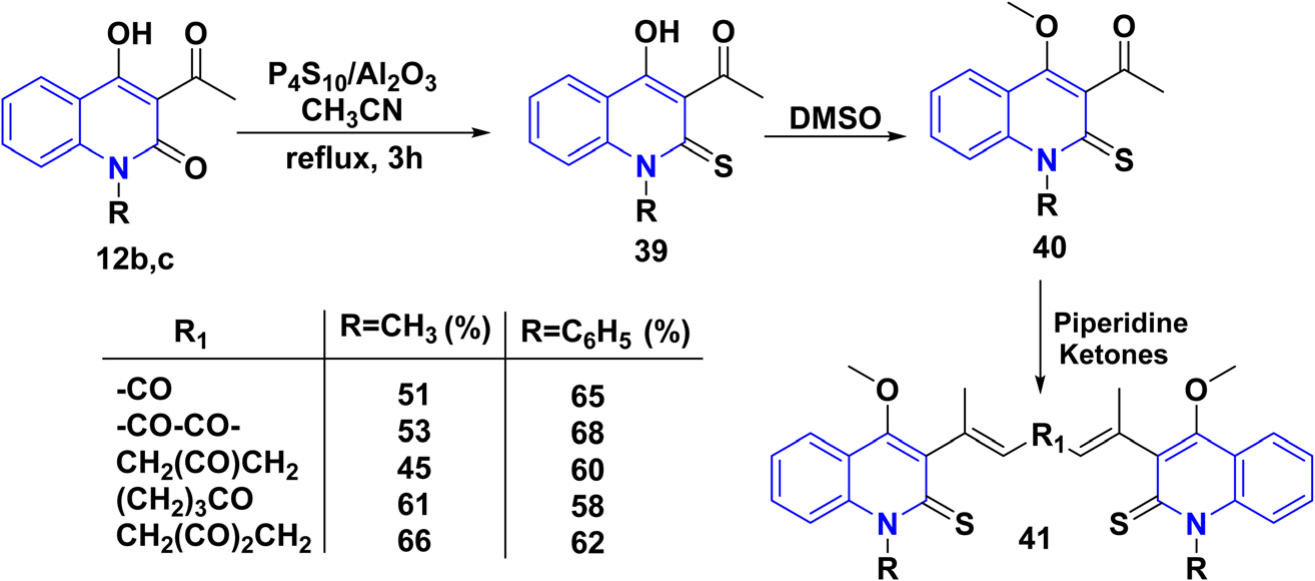
Synthetic route of 41 from 12b,c.

Compounds 12b,c underwent thionation using phosphorus pentasulfide, aluminum oxide as a catalyst/CH_3_CN. The thionated compound 39 by dimethyl sulfoxide (DMSO) underwent *o*-methylation of the hydroxyl group (enol), yielding 40 as a sole product, which prevents keto–enol tautomerism at the 3rd and 4th positions, which could have interfered with the Aldol condensation reaction. Whereby, compound 40 was further reacted with various ketones through Aldol condensation in piperidine as a basic medium, to produce 1-(4-methoxy-2-thioxoquinolin-3-yl)ethenone 41 (Scheme 13).^154^

**Scheme 14:**
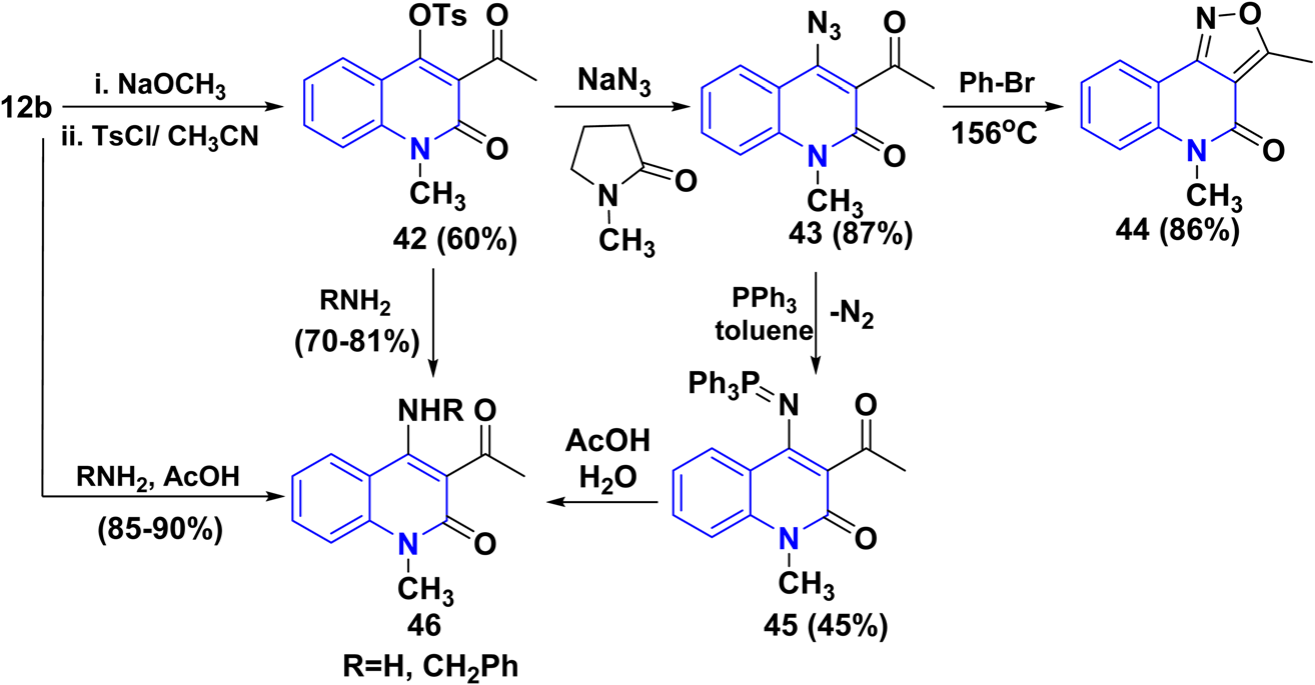
Transformation of the hydroxyl group of 12b.

Tosylation of the sodium salt of 12b furnished a very reactive compound 3-acetyl-4-tosyloxyquinolone 42, which was used as the key compound to synthesize *o*-acetylazido-quinolinone 43*via* transformation of the tosyloxy group to the azido group by using NaN_3_/*N*-methylpyrrolidone as solvent. Followed by ring closure through elimination of nitrogen gas by thermal heating in bromobenzene at 156 °C, 3-acetylazido-quinolinone afforded angular isoxazolo[4,3-*c*]quinolones 44. The azide 43 undergoes the Staudinger reaction with triphenylphosphane, yielding the phosphazene 45*via* nitrogen loss. The hydrolysis of 45 in AcOH (80%) produced the 4-aminoquinolone 46. This amine can also be produced by reacting tosyloxyquinolone 42 with ammonia. Similarly, benzylamine combines with 42 to yield substituted quinoline 46 (Scheme 14).^155–157^

**Scheme 15:**
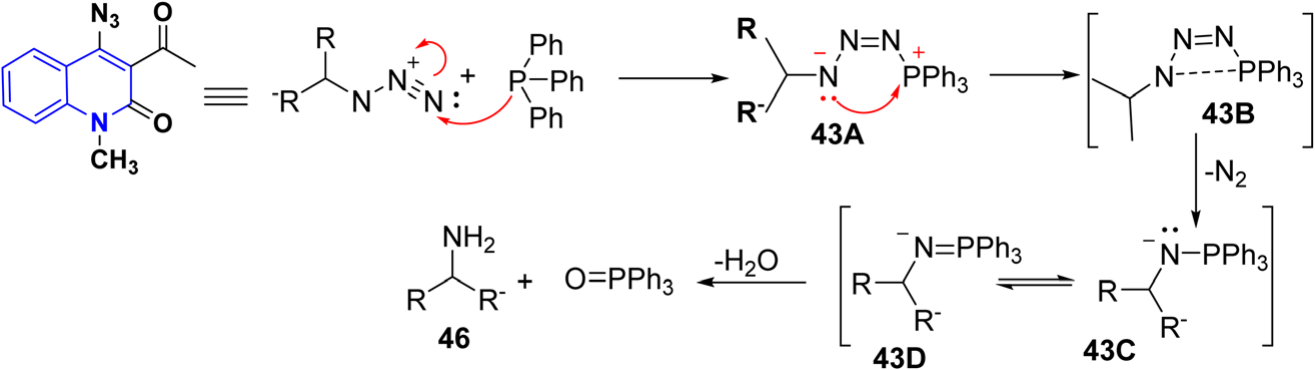
Mechanism of the Staudinger reaction.

Mechanism of Staudinger reaction *via* very mild reduction of the azide 43 with triphenylphosphane yields the phosphazene 43B by loss of nitrogenous gas. The hydrolysis of 43C/43D in AcOH (80%) produced the 4-aminoquinolone 46 (Scheme 15).^155^

**Scheme 16:**
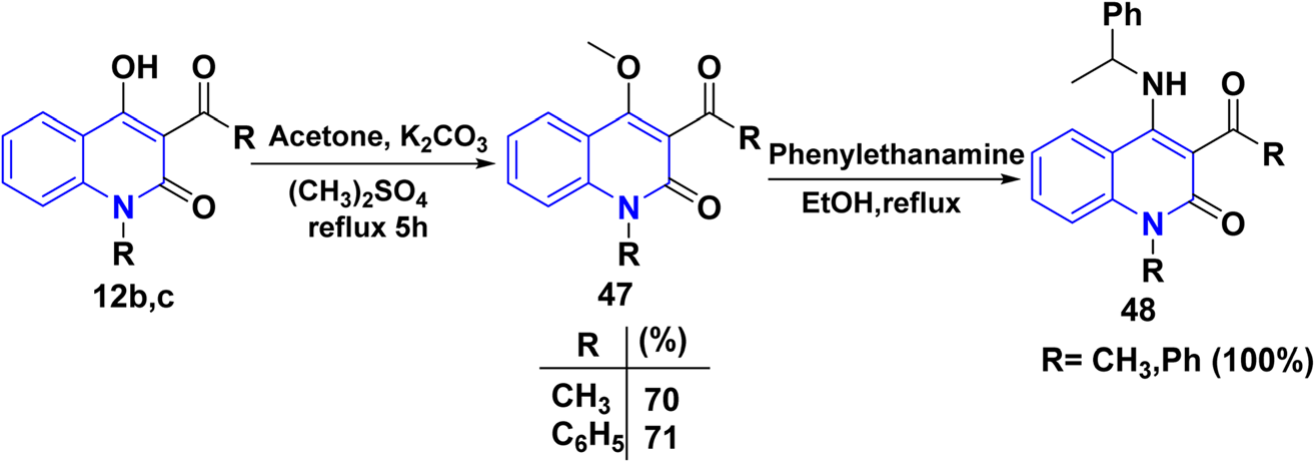
Synthesis of phenylethylaminoquinolinone 48.

Whereas, methylation of compound 12b,c by dimethyl sulphate, a potent methylating agent, in acetone, which replaced a hydrogen atom in the hydroxyl group. This process yielded a single product 3-acetyl-4-methoxyquinolinone 47.^158,159^ Following this interpretation, the product reacted with a primary amine in EtOH, yielding phenylethylaminoquinolin-2-one 48 (Scheme 16).^158^

**Scheme 17:**
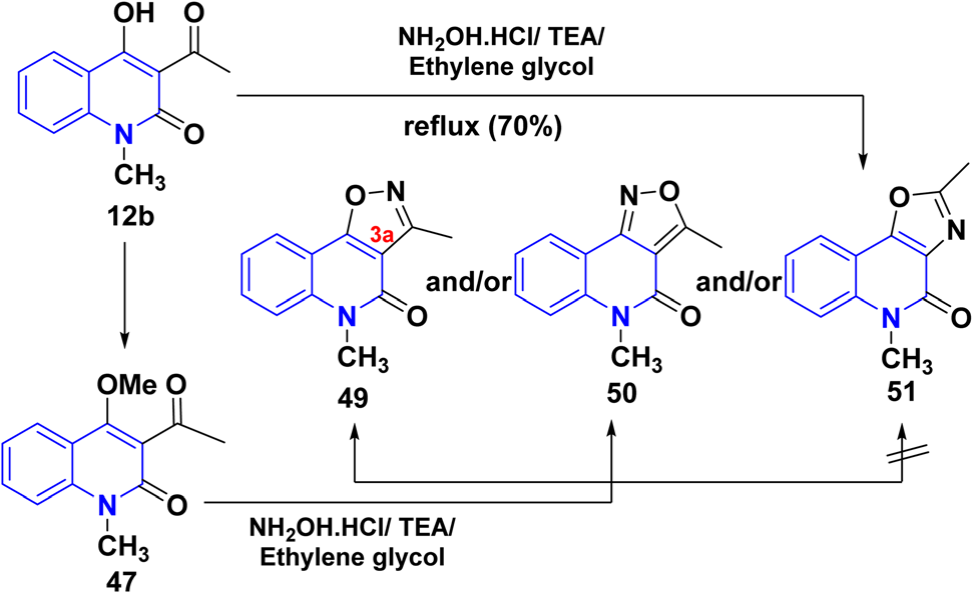
Synthesis of isoxazoloquinolinone.

Treatment of 3-acetyl-4-methoxyquinolinone 47 was observed by the Chimichi group^158^ with 1,2-bisnucleophiles, such as hydroxylamine, to synthesize fused isoxazolo[4,5-*c*]-and/or isoxazolo[4,3-*c*]quinolin-4(5*H*)ones, which gave three different products 49–51 depending on the conditions (Scheme 17). As a result, the utilization of either hydroxylamine hydrochloride or its free base leads to the formation of two distinct products. The obtained product was through a reaction of 47 with NH_2_OH·HCl is structurally assigned to the regioisomeric isoxazolo[4,5-*c*] or isoxazolo[4,3-*c*]quinolin-4(5*H*)one in the following ways:

(a) The presence of a three-bond connection between C-3a and the methyl group at position 3 of compounds 49 and 50 led to the exclusion of the oxazole structure 51.

(b) The chemical shift of the C-3 atom being analyzed, which resembles that of a 3-substituted isoxazole more than a 5-substituted one, was used as a preliminary criterion to distinguish between the two regioisomeric isoxazole skeletons. In contrast, compound 47 exclusively and preferentially generates the regioisomeric isoxazolo[4,5-*c*]- or isoxazolo[4,3-*c*]quinolin-4(5*H*)on e (compounds 49 and 50, respectively).^158^

**Scheme 18:**
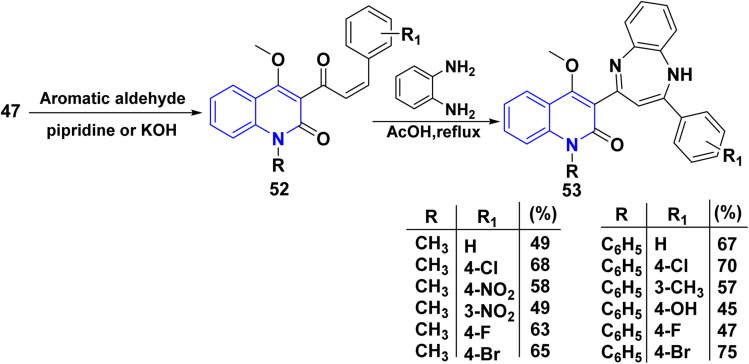
Claisen–Schmidt reaction of compound 47.

Whereby, synthesis of *α*,*β*-unsaturated ketones 52 was achieved through Claisen–Schmidt reaction of compound 47 with various aromatic aldehydes in glacial AcOH. Michael addition reaction of *o*-phenylenediamine to *α,β*-unsaturated ketone 52 afforded binary diazepin-quinolinones 53 (Scheme 18), whereas derivatives of 53 have shown gratifying sedative, anxiolytic, and muscle relaxant activities.^159^

**Scheme 19:**
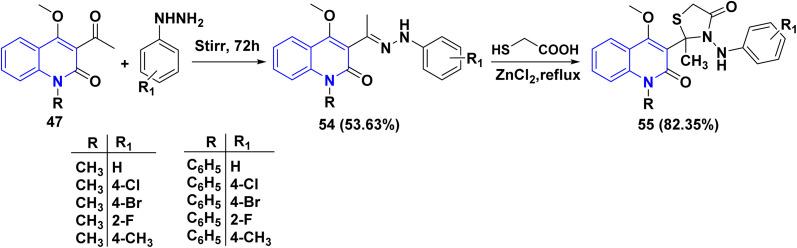
Cyclization reaction of 54 to yield 4-thiazolidinone derivatives 55.

Methylated compound 47 was reacted with substituted phenylhydrazines, which resulted in the formation of hydrazone 54, which were then cyclized with thioglycolic acid in methanol using zinc chloride (ZnCl_2_) as catalyst, affording 4-thiazolidinone derivatives 55 (Scheme 19).^160^

**Scheme 20:**
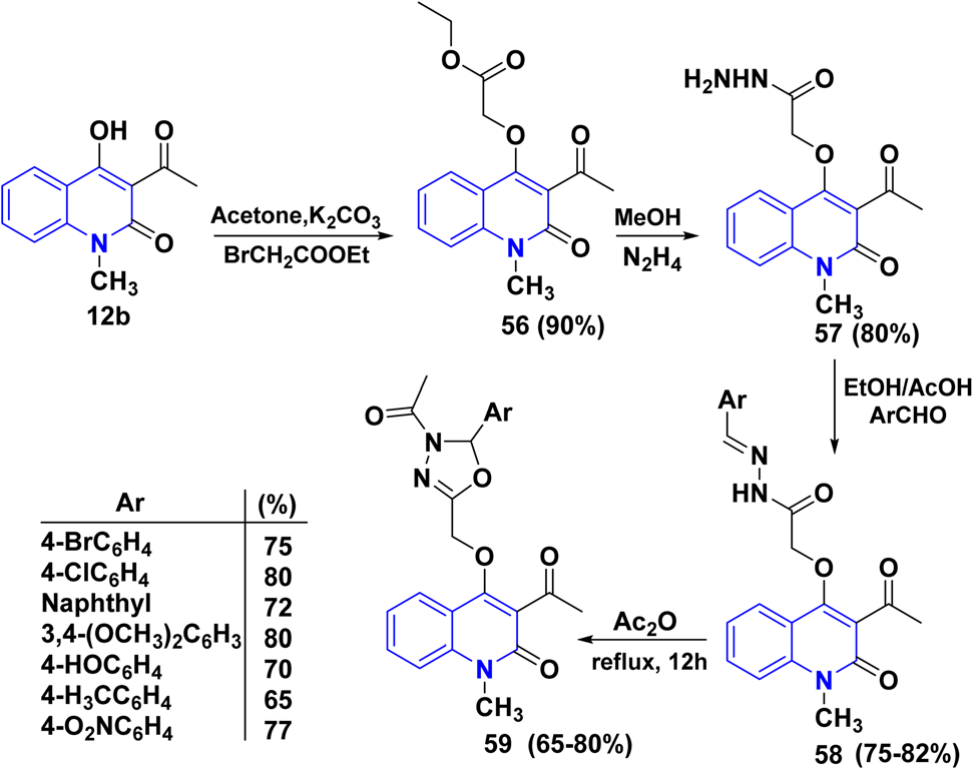
Synthesis of oxadiazolylquinolinone derivatives 59.

The *o*-alkylation of 12b with ethyl bromoacetate in anhydrous acetone and K_2_CO_3_ led to the formation of ethyl oxoquinolinoxyacetate 56. Hydrazinolysis of 56 with hydrazine hydrate in methanol at room temperature (RT) yielded hydrazide compounds 57 in 80%, after which heating hydrazide compound 57 with different aromatic aldehydes in refluxing EtOH yielded derivatives 58. Finally, compounds 58 were refluxed with Ac_2_O to obtain the corresponding substituted 1,3,4-oxadiazolyl)methoxy)-1-methylquinolinone 59 (Scheme 20).^161^

##### 4.1.1.3. Aminoalkylation

**Scheme 21:**
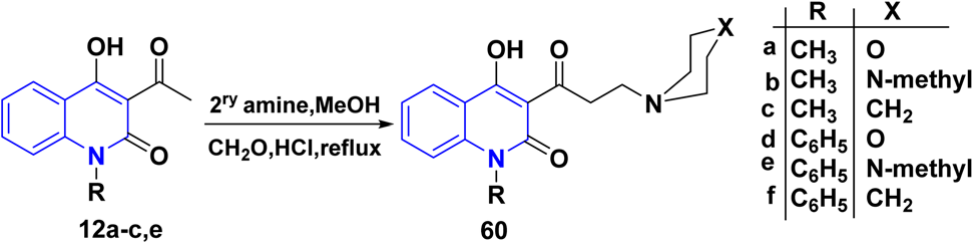
Synthesis of Mannich base 4-hydroxy-3-(3-morpholinopropanoyl)quinolinone 60.

AHQ 12a–f were subjected to Mannich reaction with formaldehyde and morpholine salt in boiling MeOH for 6 h, furnishing Mannich base 4-hydroxy-3-(3-morpholinopropanoyl) quinolinone 60 (Scheme 21).^162^

**Scheme 22:**
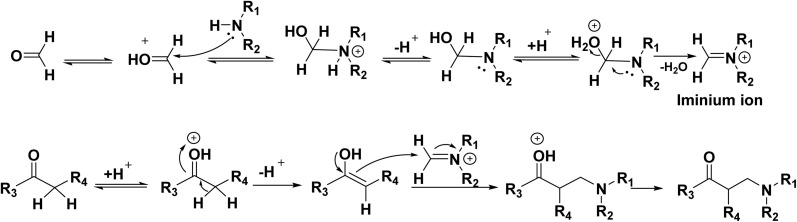
The proposed Mannich reaction mechanism.

The Mannich reaction mechanism starts with the reaction between formaldehyde and amine, leading to the formation of the iminium ion. The enol form of the organic compound is obtained *via* tautomerization of the ketone form. The reactive iminium ion is attacked by this enol form, which ultimately produces the necessary *β*-amino-carbonyl molecule or Mannich base (Scheme 22).

#### 4.1.2. Halogenation and its synthetic value for constructing various heterocyclic systems

##### 4.1.2.1. Synthesis of 3-dichloroacetyl derivative and its utility

**Scheme 23:**
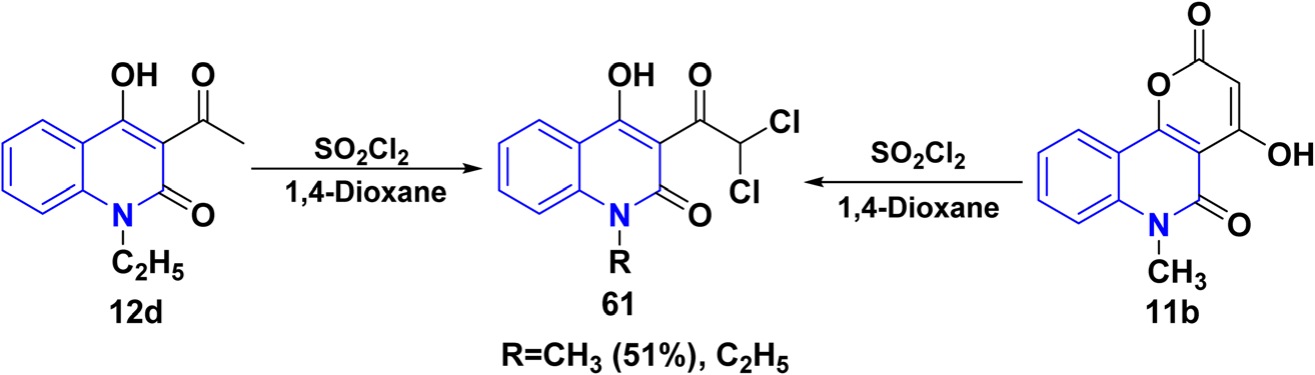
Chlorination of acetylquinolinone 12d and pyranoquinolindione 11b.

Among organic synthesis transformations, chlorination holds one of the most fundamental reactions that can be achieved directly by using molecular chlorine or chlorinating agents. Thus, the electrophilic chlorination reaction of 12b,d with sulfuryl chloride (SO_2_Cl_2_) in 1,4-dioxane yielding 3-dichloroacetyl derivative 61, also by heating pyranoquinolindione 11b with SO_2_Cl_2_ (Scheme 23).^163,164^

Treatment of compound 61 with some 1,3-binucleophilic reagents, namely, guanidine hydrochloride, cyanoguanidine, and thiourea, was carried out under reflux in DMF, to afford the corresponding 3-imidazolylquinolinones 62. While the reaction of 61 with NaN_3_ in DMF occurred at ambient temperature, the intermediate diazido compound 63A yielded the tetrazole 63 under loss of N_2_ gas. Condensation of compound 61 with 1,4-*N*,*N*-binucleophiles such as *o*-phenylenediamine and 1,6-diaminopyridine derivative 64 under reflux in DMF, afforded the quinoxalinylquinolinone 65 and the pyridotriazine derivative 66, respectively.^163^

**Scheme 24:**
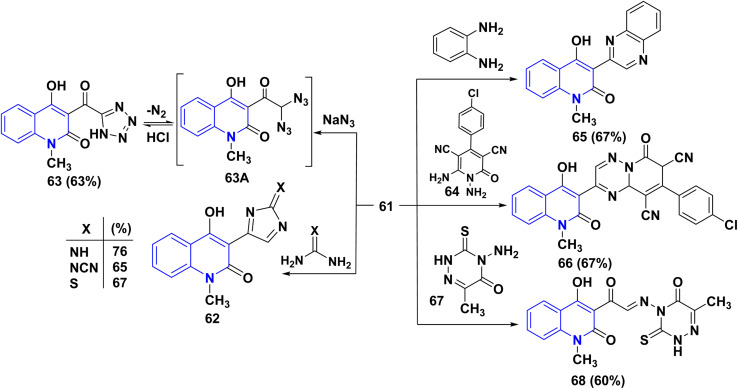
Treatment of 3-dichloroacetyl 61 with nucleophilic nitrogenous reagents.

Reaction of 61 with 4-aminotriazine derivative 67 was carried out in boiling pyridine. Interestingly, the only product that was obtained revealed the absence of chlorine; analysis results indicate that a nucleophilic replacement took place with the leaving of a chlorine atom, which is followed by elimination of hydrogen chloride, leading to the *α*-iminone 68 (Scheme 24).^164^

**Scheme 25:**
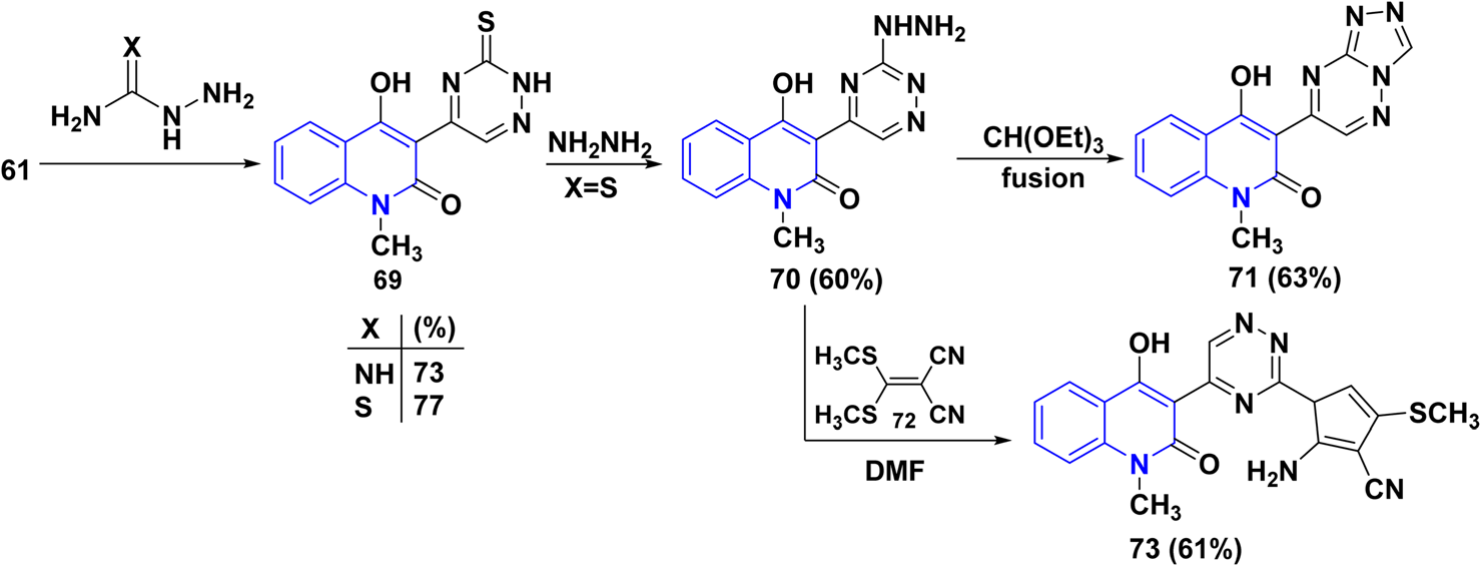
Hydrazinolysis of the 1,2,4-triazine derivative 69.

Reaction of compound 61 with aminoguanidine and thiosemicarbazide as 1,4-binucleophiles, under reflux in DMF, yielded the corresponding 1,2,4-triazine derivatives 69. Hydrazinolysis of 69 in DMF produced the hydrazinotriazine 70, Thermal cyclocondensation reaction of 70 with CH(OEt)_3_ under fusion condition was carried out to get the triazolotriazine derivative 71 whereas, reaction with [*bis*(methylthio)methylene] malononitrile 72, in DMF under reflux, gave the compound 73 (Scheme 25).^163^

**Scheme 26:**
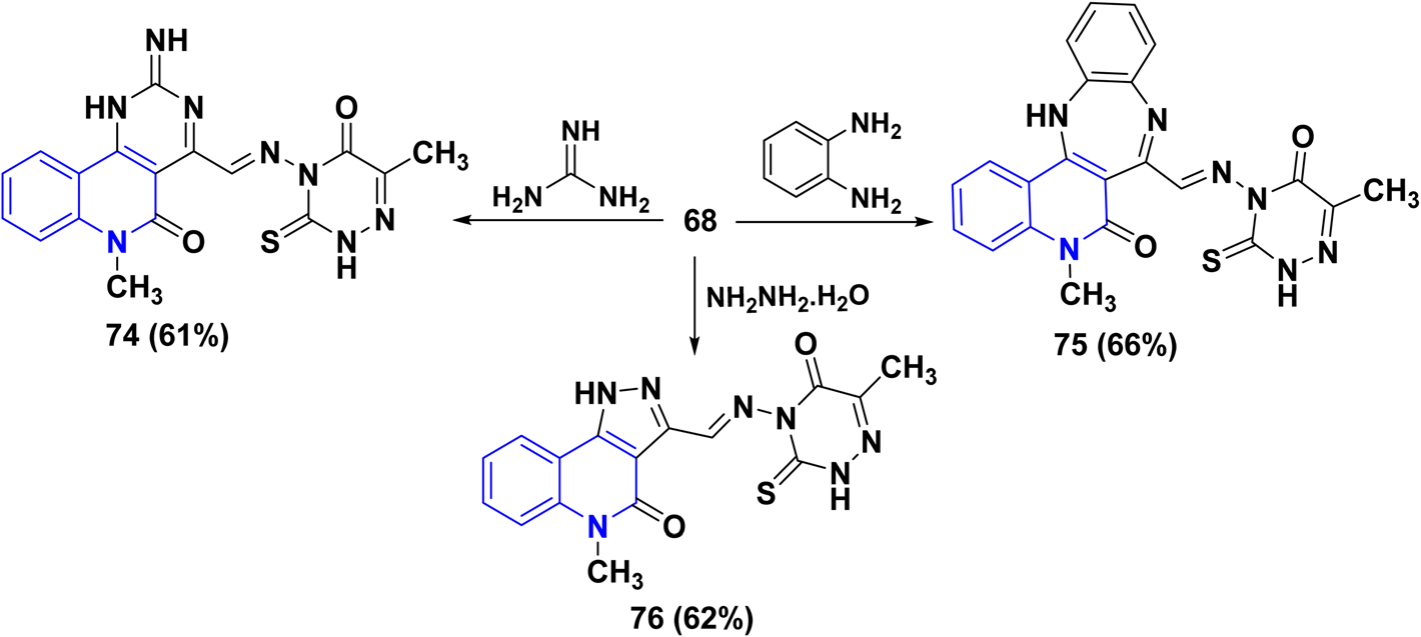
Synthesis of pyrimidine, diazepine, and pyrazole heterocycles from 68.

*α*-Iminone 68, as an interesting starting material, was subjected to interaction with some binucleophilic reagents to synthesize new quinolinone systems fused with pyrimidine, benzodiazepine, and pyrazole heterocycles. Therefore, treatment of *α*-iminone 68 with guanidine, *o*-phenylenediamine, and hydrazine hydrate, in DMF under reflux, afforded the pyrimidoquinolinone 74 and benzodiazepinoquinolinone 75, and pyrazoloquinolinone 76, respectively (Scheme 26).^163^

##### 4.1.2.2. Synthesis of 3-(bromoacetyl)-4-hydroxyquinolinone and its benefits

**Scheme 27:**
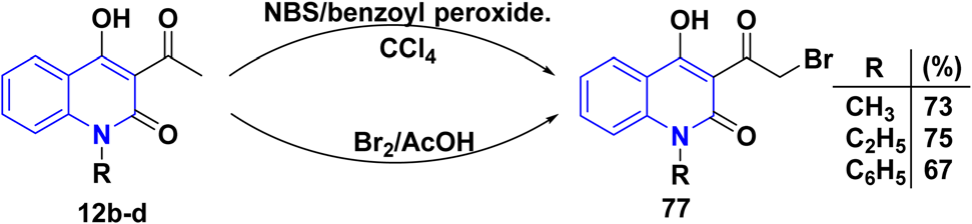
Bromination of *N*-substituted-3-acetyl-4-hydroxyquinolinones 12b–d.

The bromination of AHQs 12b–d can be performed using bromine or *N*-bromosuccinimide (NBS) in the presence of benzoyl peroxide. 3-(Bromoacetyl)-4-hydroxyquinolinone derivatives 77 were synthesized by the free radical bromination mechanism of acetyl 12b–d using NBS in dry carbon tetrachloride (CCl_4_) with a catalytic amount of benzoyl peroxide (3 mol%) *via* homolytic fission,^165^ also reaction of 12b–d with bromine in the presence of AcOH *via* electrophilic bromination gave the same product 77 (Scheme 27).^115,134,166,167^

**Scheme 28:**
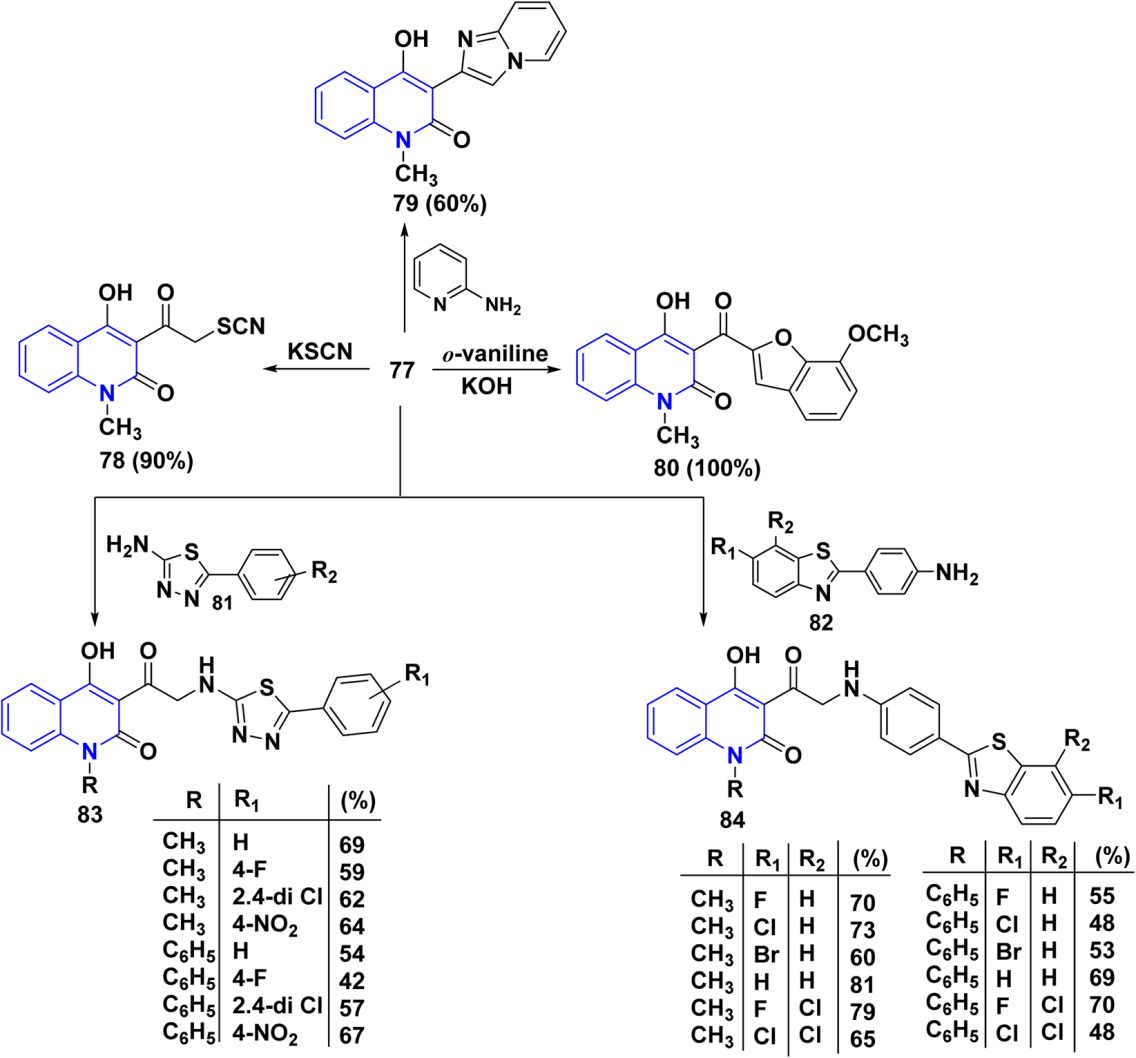
Reactions of bromo-derivatives 77 with variable reagents.

The desired thiocyanate 78 was obtained from the reaction of 77 with potassium thiocyanate. Meanwhile, phenacyl halides were used for the alkylation of 2-aminopyridine, followed by cyclization to imidazo[1,2-*a*]pyridines. Thus, when 77 was heated with 2-aminopyridine in ethanol, 4-hydroxy-3-imidazoyl methylquinolinone 79 was formed.^167^ Moreover, condensation of bromo-derivatives 77 with *o*-vaniline gave 4-hydroxy-3-(7-methoxybenzofuran-2-carbonyl)-1-methylquinolin-2(1*H*)-one 80. Whereas, compound 77 is a versatile intermediate in synthesizing compounds 83 and 84. So that, the synthesis of 83 were achieved through condensation reaction of 77 with various thiadiazole 81 and the observed compound benzo[*d*]thiazol-2-yl-phenylglycylquinolinone 84 were synthesized in good yield (60–81%) through the reaction of disubstituted-2-benzo[*d*]thiazole 82 with 77 in the presence of AcOH (Scheme 28).^166,168^

#### 4.1.3. Formylation of AHQ and its uses

**Scheme 29:**
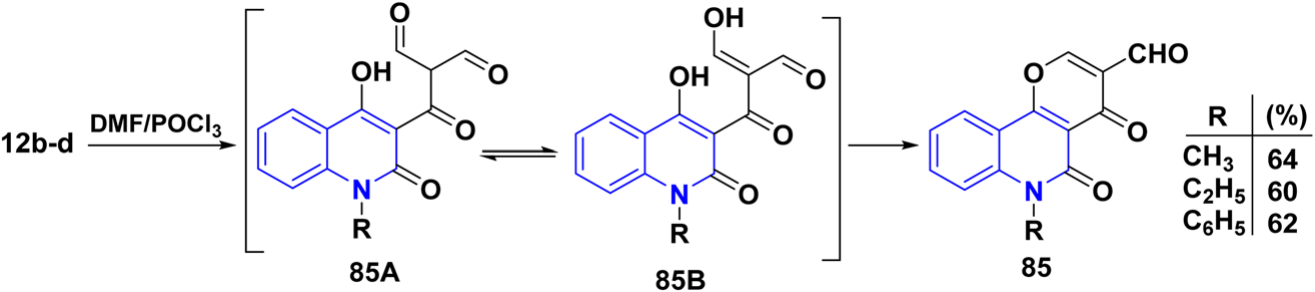
Vilsmeier–Haack reaction of skeletons 12b–d.

**Scheme 30:**
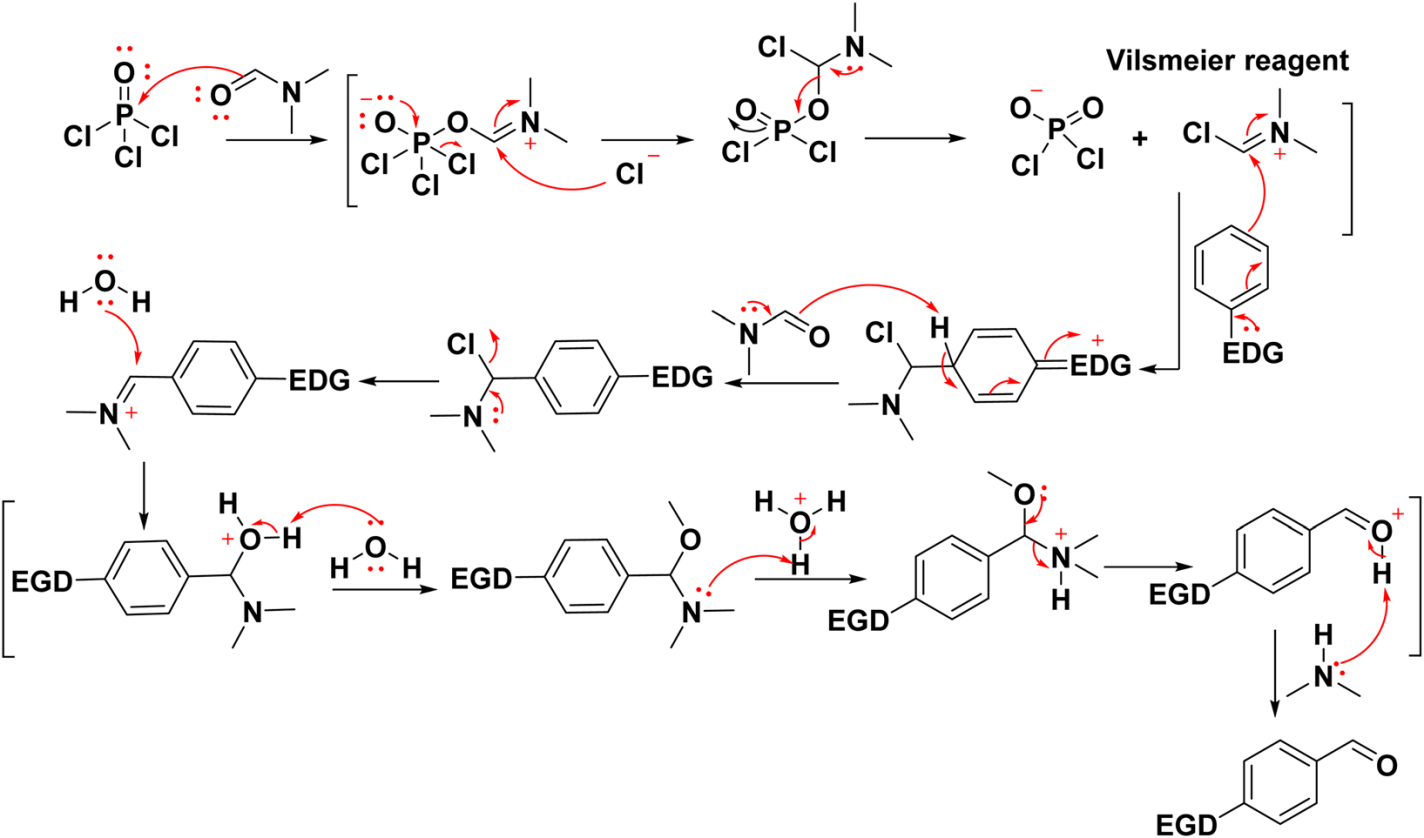
Plausible mechanism of Vilsmeier reaction.

The Vilsmeier–Haack reaction was known since 1927,^169^ which uses DMF, an acid chloride and an aqueous work-up to change an aromatic ring with a lot of electrons into an aryl aldehyde. The “Vilsmeier reagent” is an iminium salt that is created when DMF reacts with acid chloride at the start of the mechanism (Scheme 30). Moreover, double formylation of the methyl group present in acetylquinolone 12b–d with subsequent *in situ* cyclization of intermediates 85A and 85B yielded pyrano[3,2-*c*]quinoline-3-carboxylaldehyde derivatives 85*via* Vilsmeier–Haack reaction of acetylquinolinones 12b–d (Scheme 29).^137,170^

Ibrahim *et al.*^170^ independently reported the treatment of 85 with various nucleophilic reagents affording pyrano[3,2-*c*]quinolines *via* ring-opening followed by ring closure (RORC).

**Scheme 31:**
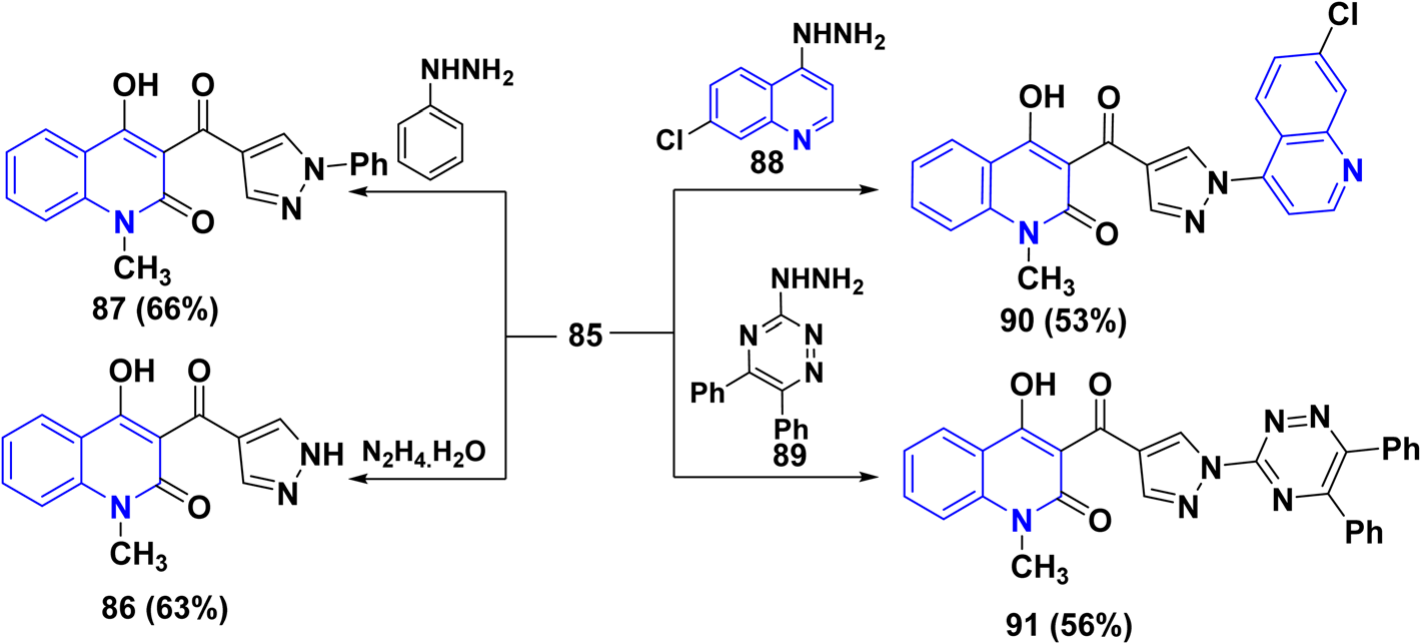
Synthesis of pyrazolylquinolinone skeletons.

Also, reaction of carboxaldehyde 85 with hydrazine hydrate in refluxing EtOH yielded 4-hydroxypyrazol-4-ylcarbonylquinolin-2-one 86, *via* the non-isolable hydrazone intermediate, which underwent intramolecular nucleophilic attack of NH_2_ at the C-2 position with concomitant *γ*-pyrone ring opening *in situ*. In the same way, the condensation reaction of 85 with phenyl hydrazine in EtOH containing a catalytic amount of TEA afforded phenylpyrazole derivative 87. Whereby, the condensation of carboxaldehyde 85 with hydrazinylquinoline quinoline 88 and 3-hydrazinyl-1,2,4-triazine-1,2,4-triazine 89 under the same reaction conditions was achieved yielding quinolinylpyrazolylquinolinone 90 and triazinylpyrazolylquinolinone 91, respectively (Scheme 31).^170^

**Scheme 32:**
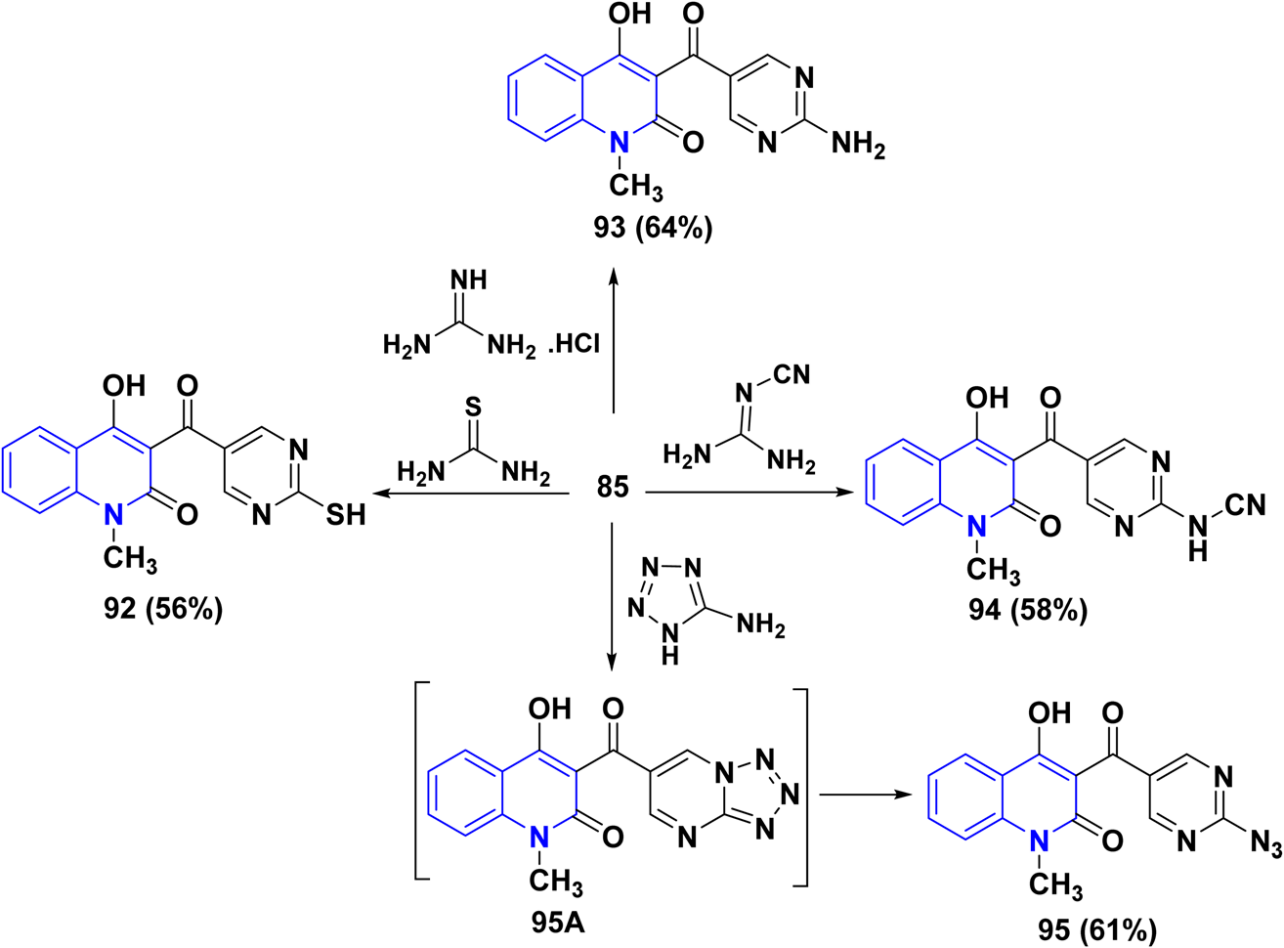
Synthesis of six-membered heterocyclic scaffolds.

Furthermore, aldehyde 85 was subjected to react with some 1,3-*N*,*N*-binucleophiles such as thiourea, guanidine hydrochloride and cyanoguanidine in ethanolic KOH yielded the corresponding pyrimidine derivatives 92, 93 and 94, respectively. Whereby, refluxing of 85 with 5-aminotetrazole in EtOH afforded 3-[(2-azidopyrimidin-5-yl)carbonyl]4-hydroxyquinolin-2-one 95 through non-isolable tetrazolo[1,5-*a*]pyrimidine intermediate 95A (Scheme 32).

Meanwhile, condensation of aldehyde 85 with 3-aminotriazole in EtOH yielded triazolo[4,3-*a*]pyrimidine 96 containing the quinolinylcarbonyl moiety. Ultimately, condensation of 85 with 2-aminobenzimidazole in EtOH containing one crystal of *p*-toluenesulfonic acid afforded pyrimido[1,2-*a*]pyrimidine derivative 97. Also, reaction of 85 with 1*H*-benzimidazol-2-ylacetonitrile and *N*-benzyl-2-cyanoacetamide in EtOH containing catalytic drops of TEA yielded 98 and 99, respectively.

**Scheme 33:**
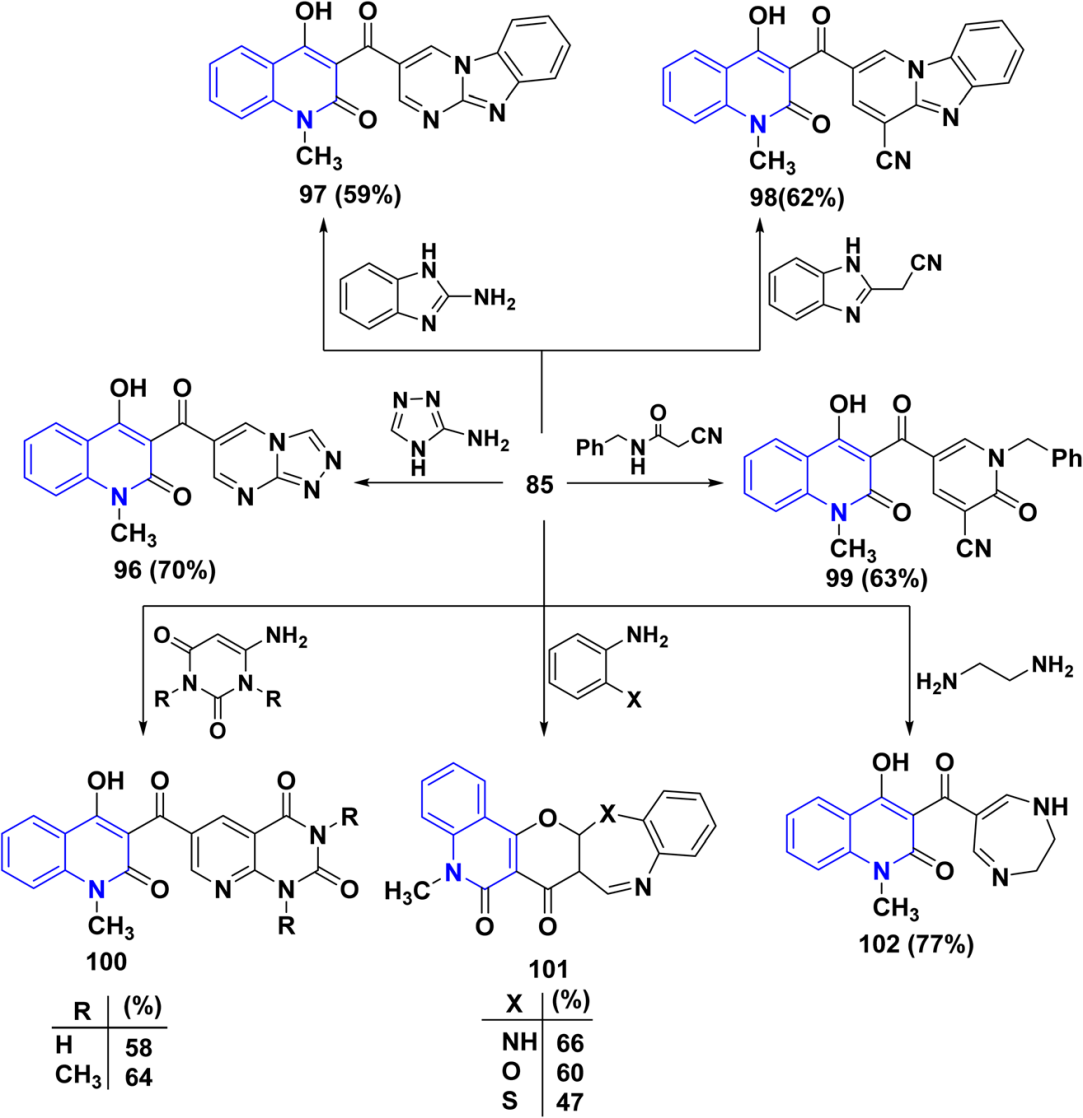
Synthesis of different heterocyclic systems containing quinolone scaffolds.

Next, condensation reaction 85 with *o*-phenylenediamine, *o*-aminophenol, and *o*-aminothiophenol in AcOH yielded the corresponding heteroannulated pyrano[3,2-*c*]quinoline derivatives 101. Whereby, reaction of 85 with 6-aminouracil and 6-amino-1,3-dimethyluracil in EtOH afforded pyrido[2,3-*d*]pyrimidines 100. Furthermore, the chemical reactivity of 85 was investigated towards different 1,4-binucleophiles. By the way, the condensation reaction of 85 with ethylenediamine in EtOH yielded 1,4-diazepinylcarbonylquinolin-2-one 102. The reaction was accomplished through the synthesis of the corresponding Schiff base intermediate, followed by an intramolecular nucleophilic addition at the C-2 position associated with *γ*-pyrone ring opening to afford 102 as the final product (Scheme 33).^170^

**Scheme 34:**
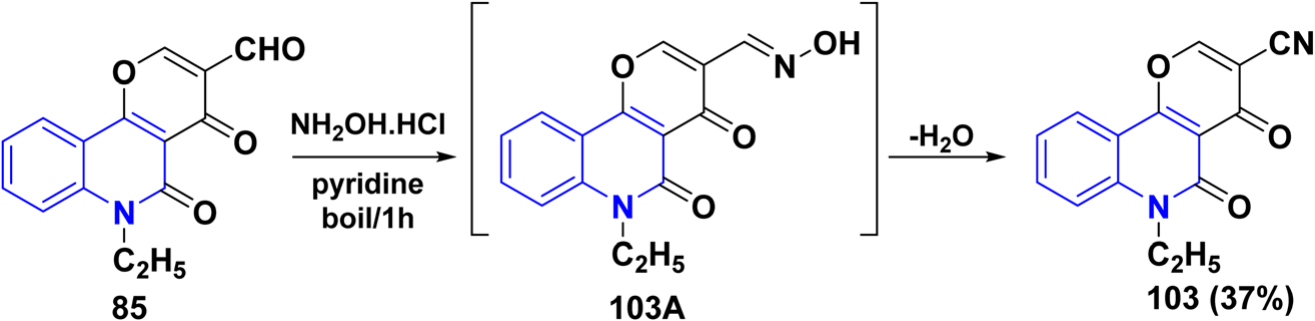
Synthesis of tricyclic carbonitrile derivative 103.

In refluxing pyridine, the reaction of tricyclic aldehyde 85 with NH_2_OH·HCl yielded the corresponding carbonitrile derivative 103, through the formation of non-isolable oxime 103A, after *in situ* dehydration step (Scheme 34).^137^

**Scheme 35:**
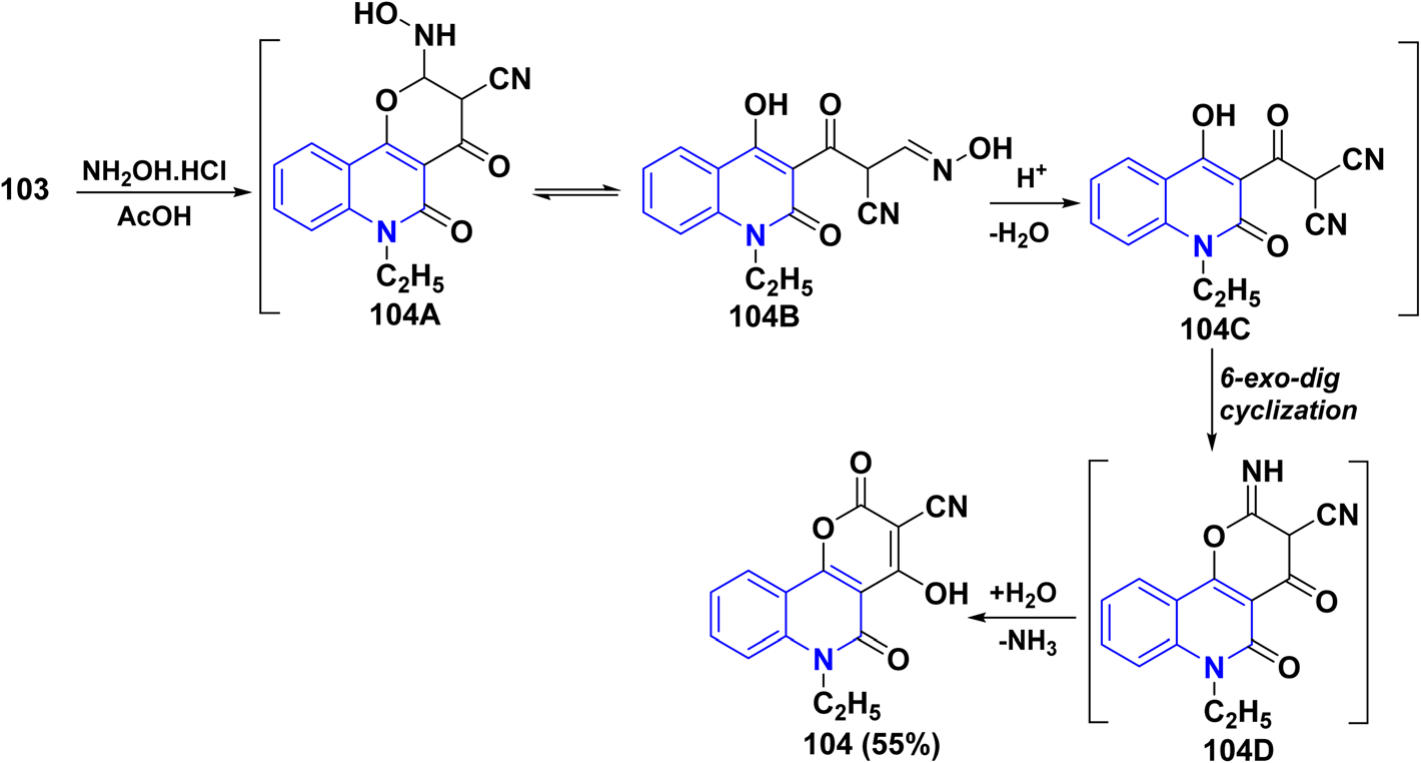
Synthesis of 4-hydroxypyrano[3,2-*c*]quinoline-3-carbonitrile derivative 104.

Reaction of aldehyde 85 with NH_2_OH·HCl, in AcOH, did not yield either oxime 103A or carbonitrile 103 but gave unexpected product showed that dioxopyrano[3,2-*c*]quinoline-3-carbonitrile 104 through two molecules of hydroxylamine reacted step-wisely with 85 yielded 103 which is regarded as a highly reactive cyclic push–pull system that quickly produces the adduct 104B, which exists in equilibrium due to ring-chain tautomerism. The dehydration of oxime tautomer readily afforded the corresponding intermediate 104C. Intramolecular 6-*exo-dig* cyclization type of intermediate 104C gave 2-iminopyranoquinoline 104D following that hydrolysis of 104D yielded the final product 104 (Scheme 35).^137^

**Scheme 36:**
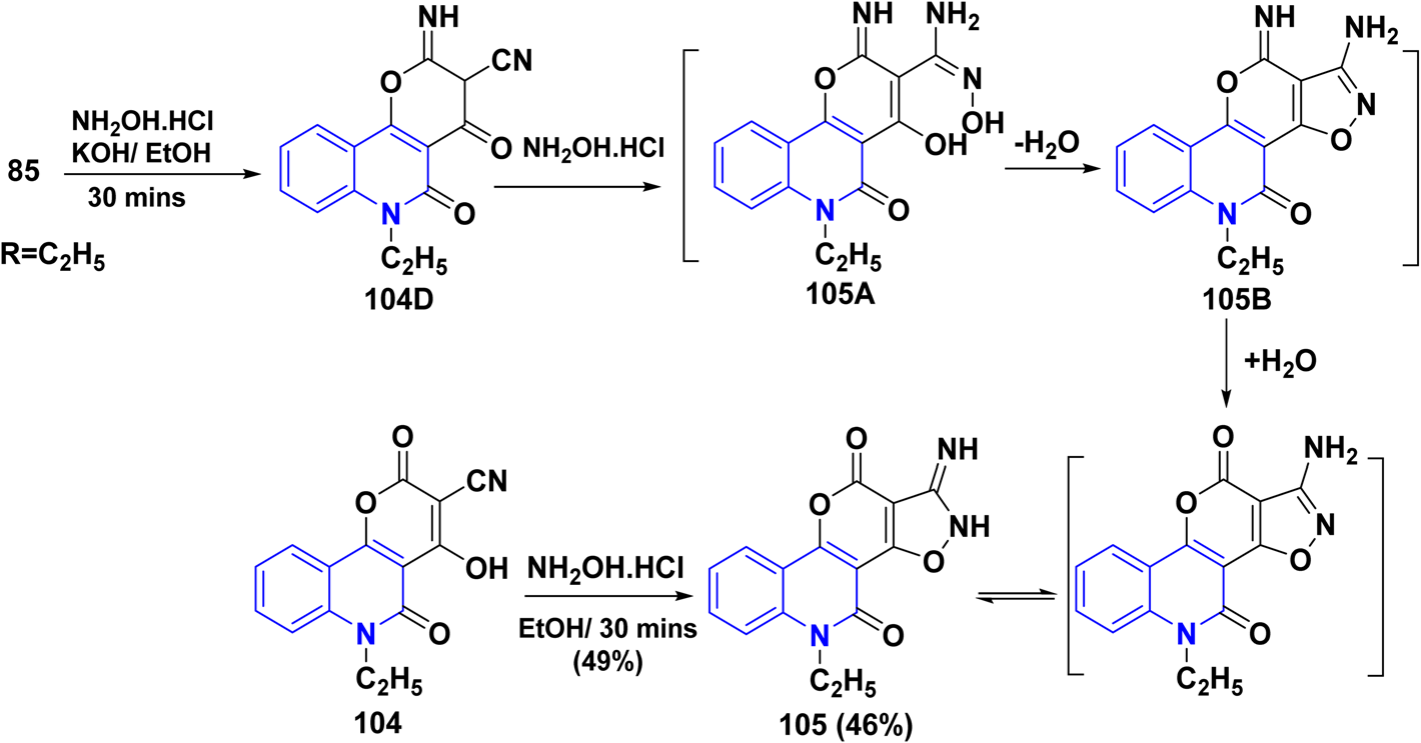
Formation of 1-amino-5-ethylisoxazolopyrano[3,2-*c*]quinolindione 105.

Reaction of aldehyde 85 with NH_2_OH·HCl, in ethanolic KOH (1%) as a basic catalyst, instead of an acidic medium, gave 105 in 46% yield. The reaction gave the same intermediate 104D, in a basic catalyzed route, which underwent the addition of another molecule of NH_2_OH·HCl led to the formation of 2-imino-5-oxopyrano[3,2-*c*]quinoline-3-carboximidamide 105A. Whereas, the cyclization of imidamide through a condensation reaction yielded isoxazole derivative 105B. After that, hydrolysis of imine 105B was achieved, leading to the final product 105. Additionally, compound 105 was accurately prepared from the reaction of 104 with NH_2_OH·HCl in refluxing EtOH (Scheme 36).^137^

#### 4.1.4. Synthesis of 1,3,2-dioxaborinino[5,4-*c*]quinolinone *via* metallation and its utility

**Scheme 37:**
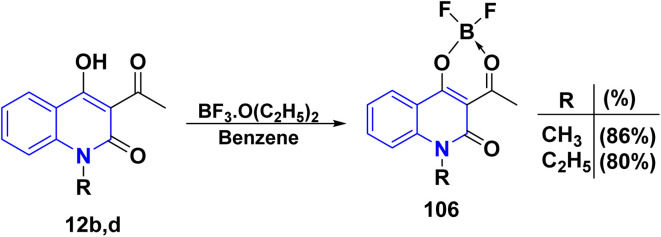
Complexation of compounds 12b,d.

The metallation reaction of acetylquinolinone 12b,d with boron trifluoride etherate BF_3_·(OC_2_H_5_)_2_ in benzene gave its boron difluoride chelate complex 106 (Scheme 37).^171^

**Scheme 38:**
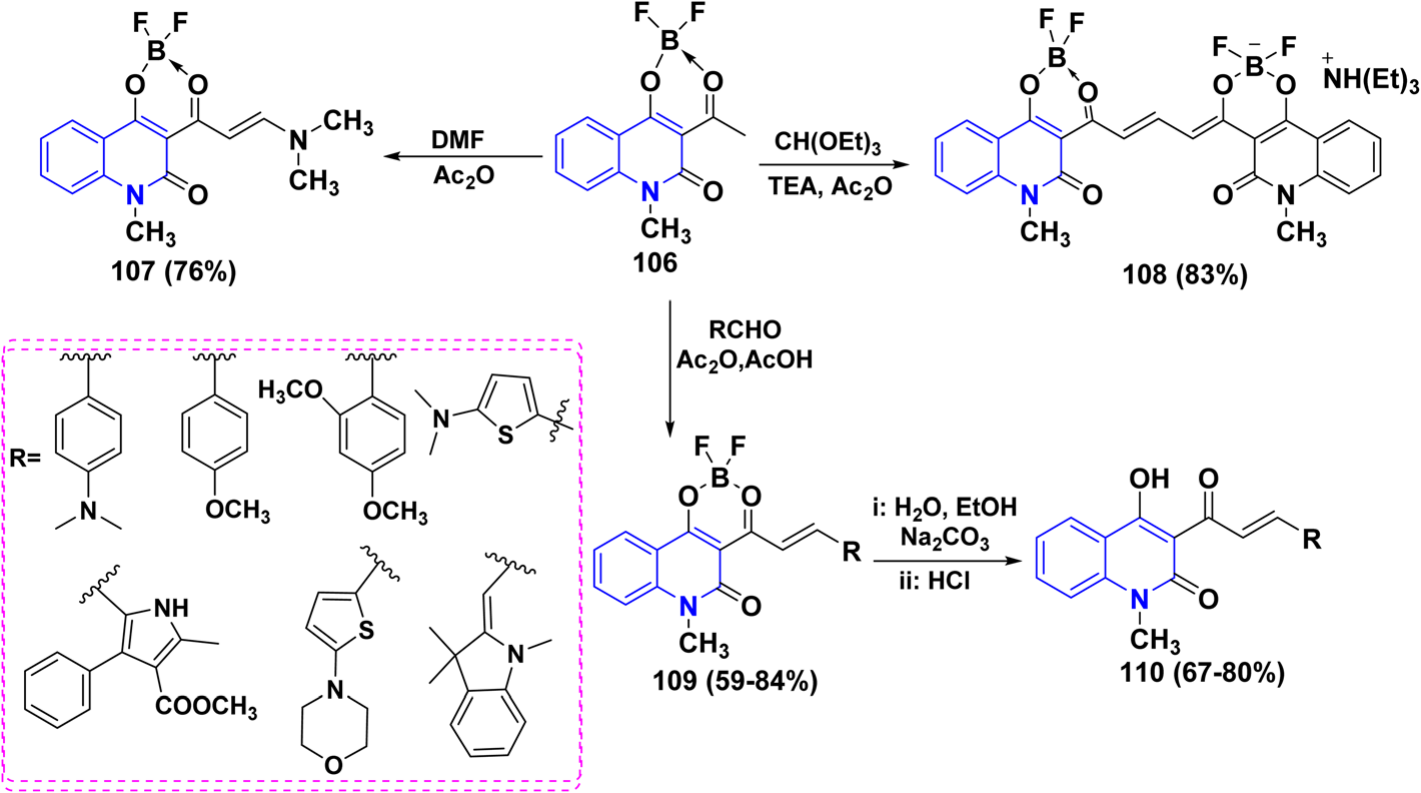
Condensation reactions on complex 106.

Reaction of complex 106 with DMF gave enamine 107, which can serve as a valuable synthon for transformations of the two-quinolinone fragment. Moreover, complex 106 was treated with different carbonyl compounds to yield the corresponding condensation products at the methyl group. For example, a reaction of two moles of complex 106 with CH(OEt)_3_ with a catalytic amount of TEA afforded symmetric polymethine dye 108. In acetonitrile, complex 108 absorbs at 581 nm, displaying noticeable fluorescence. Lastly, boron-containing styryl dyes 109 were produced by the condensation reaction of several aromatic and heterocyclic aldehydes with complex 106. Compounds 109 were hydrolyzed to produce novel 4-hydroxy-2-quinolinone derivatives 110, which exhibit fluorescence in both solid and solution states (Scheme 38).^171^

### 4.2. Nucleophilic substitution reactions

The substitution of a nucleophile at a tetrahedral or sp^3^ carbon is known as aliphatic nucleophilic substitution. Substitutions involving aliphatic nucleophiles are not glamorous or essential in the field of chemistry. The way carbonyl additions and carboxyloid replacements seem to occur in biochemistry, they do not occur in all significant processes. Rather, they are commonplace, minor reactions with significant, minor effects everywhere.

#### 4.2.1. Reactions with nitrogen bases

##### 4.2.1.1. With amines

**Scheme 39:**
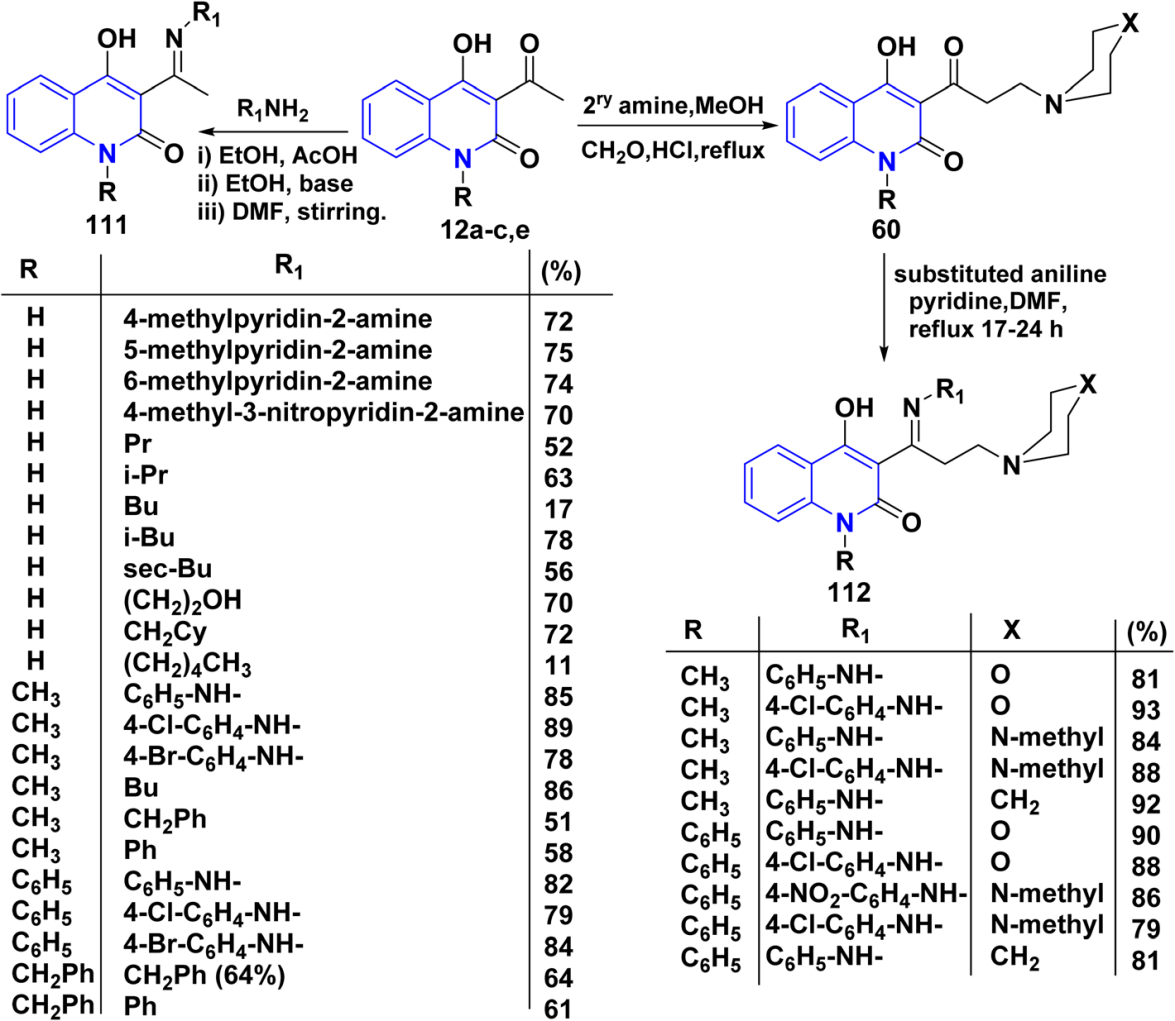
Synthesis of imine 111, Mannich base (QVIR) 60 and its imine analogue 112.

Condensation reaction of *N*-substituted (un)acetylquinolinone 12a–c,e with different amines under various conditions afforded enaminones 111.^143,155,162,172,173^ While, addition of a solution of substituted aniline in dimethylformamide (DMF) and pyridine to 60 and refluxed for 17–24 h to give 112 (Scheme 39).^162,173^

**Scheme 40:**
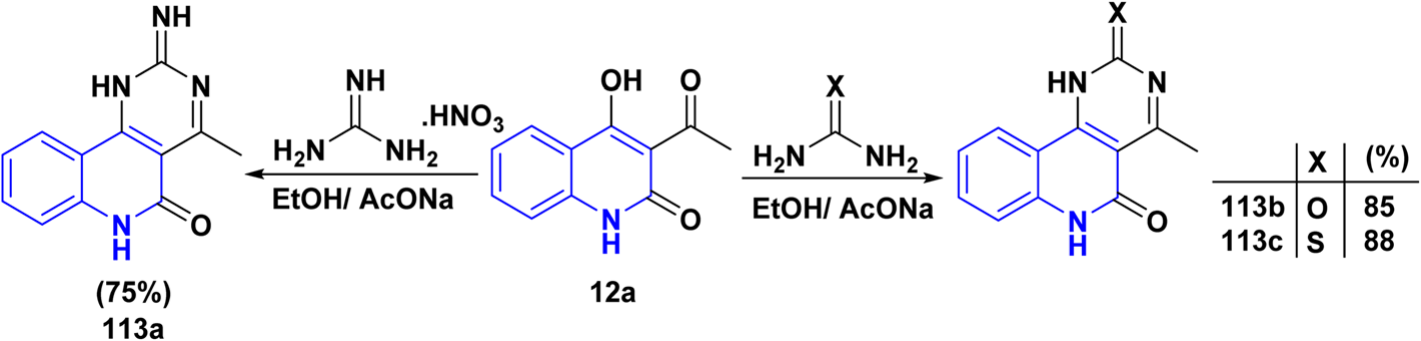
Synthesis of pyrimido[5,4-*c*]quinolinones 113a–c.

The reaction of quinolinone 12a with nitrogen bases derivatives such as guanidine nitrate, urea and thiourea in boiling ethanol using AcONa as a catalyst through nucleophilic addition of the amino group with the regioselectively at the C=O of acetyl function of 12a accompanied by intramolecular cyclocondensation afforded regioisomer pyrimido[5,4-*c*]quinolinones 113a–c (Scheme 40).^174^

##### 4.2.1.2. With hydrazines

**Scheme 41:**
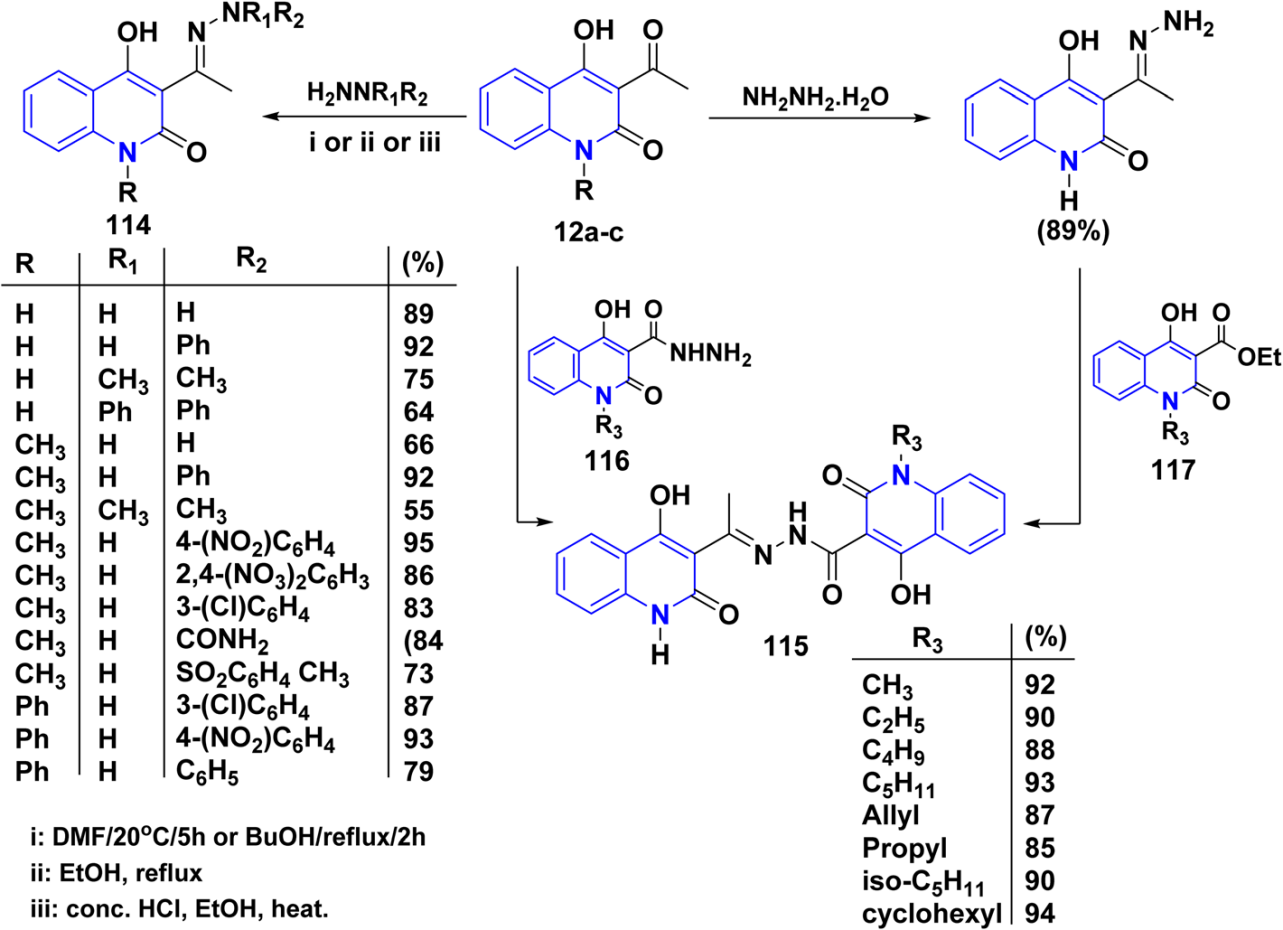
Reactions of quinolinones 12a–c with variable hydrazines.

Condensation reaction of hydrazines and arylhydrazines with AHQ derivatives 12a–c in boiling EtOH or DMF yielded corresponding hydrazones 114.^115,118,134,172^ The reaction conditions varied depending on the aryl substituents, with phenylhydrazine reacting in dimethylformamide (DMF) at ambient temperature, while nitro- and chlorophenyl hydrazines required refluxing in BuOH (Scheme 41).^135,155^

The formation of compound 115 can be accomplished through various methods. This reaction involves two straightforward and efficient approaches. The first method involves a one-step condensation of acetylquinoline 12a with hydroxyquinolone-3-carboxylic acids hydrazides 116, resulting in high yields. The second method consists of two stages: initially, treatment of acetylquinoline 12a to quinolinone, followed by acylation using ethyl hydroxyquinoline-3-carboxylates 117. This method also proves to be effective in the production of the hydrazones 115 described in this study; both methods were found to be equally valuable (Scheme 41).^115,120,134^

**Scheme 42:**
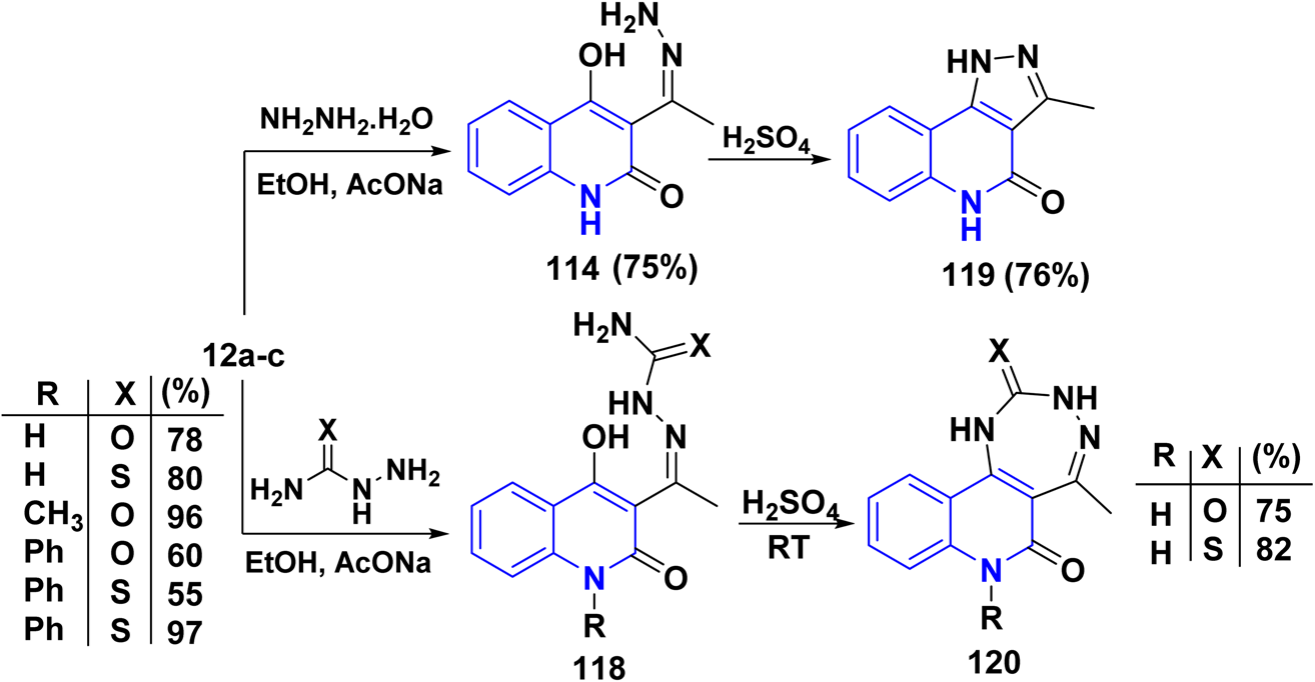
Condensation and cyclization of 114 and 118 to synthesize compounds 119 and 120.

Similarly, compounds 119 and 120 were not achieved immediately by the condensation of 12a–c with the following hydrazines as hydrazine hydrate, semicarbazide, or thiosemicarbazide but gave acyclized hydrazone 114 and 118, respectively. These products were cyclized with Conc. H_2_SO_4_ to yield angular fused pyrazoloquinolinone 119 and angular triazepinoquinolinone 120 (Scheme 42).^118,174^

**Scheme 43:**
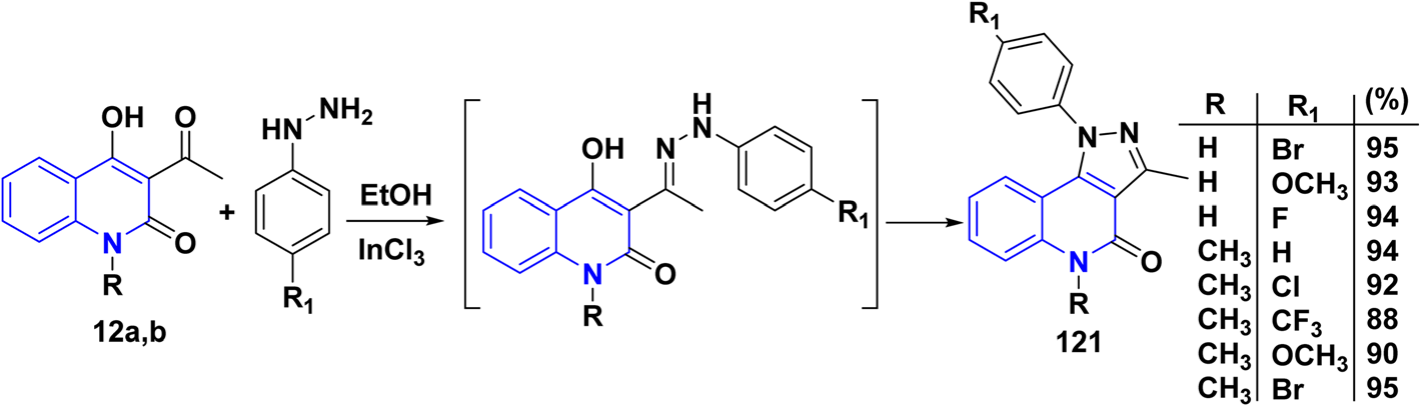
Synthesis of pyrazolo[4,3-*c*]quinoline 121.

Angular pyrazolo[4,3-*c*]quinoline derivatives 121 were synthesized under *via* enolizable 3-acetylquinolinone 12a,b with phenylhydrazine derivatives, utilizing InCl_3_ as an effective Lewis acid in providing greater yields of products 121 under microwave irradiation (Scheme 43).^175^

**Scheme 44:**
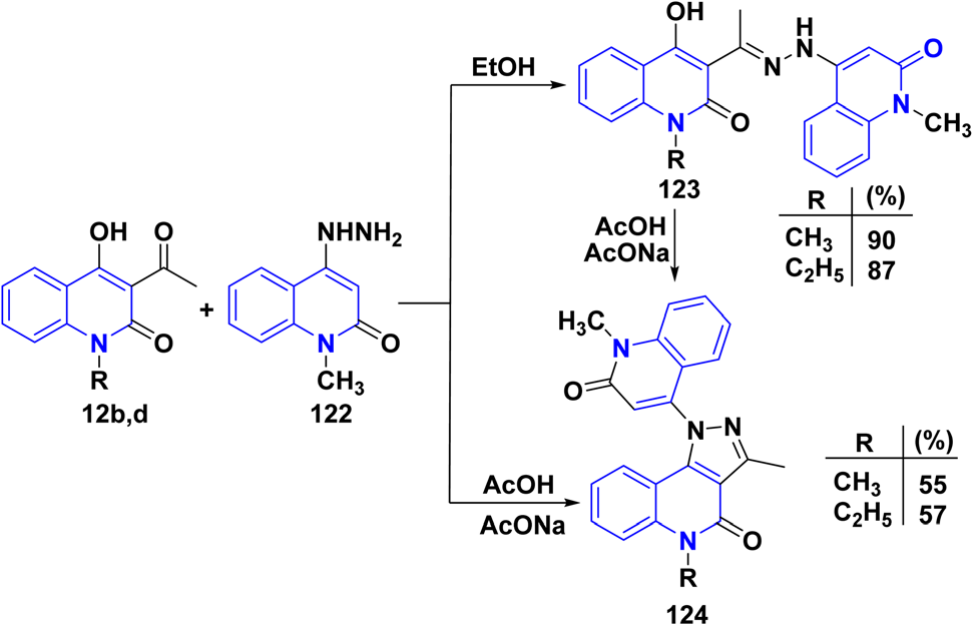
Synthesis of compounds 123 and 124 through condensation and cyclization.

Also, bis quinolinone 123 and 124 were obtained by reaction of 12b,d with hydrazinyl derivative 122 in different conditions, in hot ethanol or glacial acetic acid and sodium acetate (AcONa) (Scheme 44).^176^

**Scheme 45:**
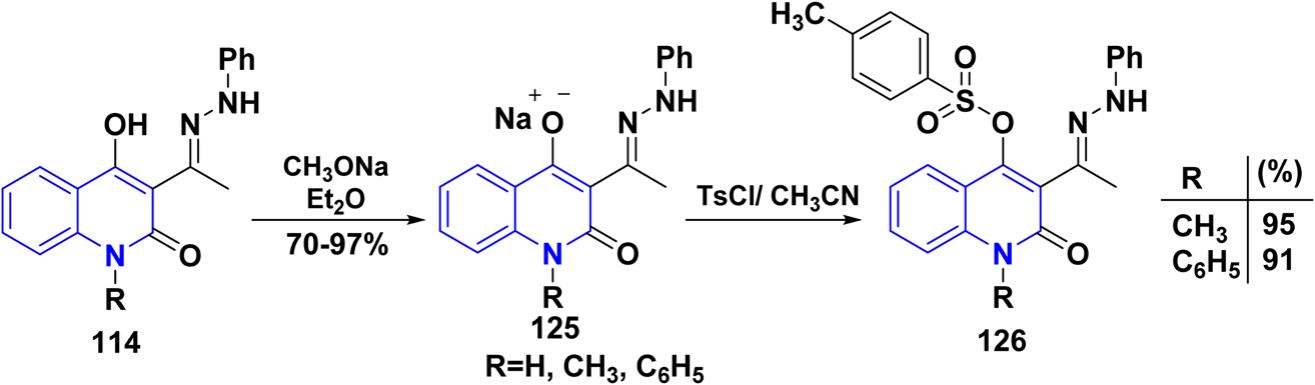
Synthesis of phenylhydrazonoquinolinylmethylbenzenesulfonate 126.

Initially, the reaction of hydrazone derivatives 114 with CH_3_ONa in diethyl ether converted the hydroxy group at position 4 to the sodium enolate group, affording 125. The reaction of sodium salts with toluenesulfonyl chloride in dry CH_3_CN yielded targeted toluenesulfonyloxy quinolones 126. Conversely, the attempts to obtain the toluenesulfonyloxy compounds 126 (R=H) were not avail However, the separation of sodium salts 125 (R=H) in good yields may be attributed to the high reactivity, in both cases, after reaction with TsCl yielded non-isolable mixture of products (Scheme 45).^135^

**Scheme 46:**
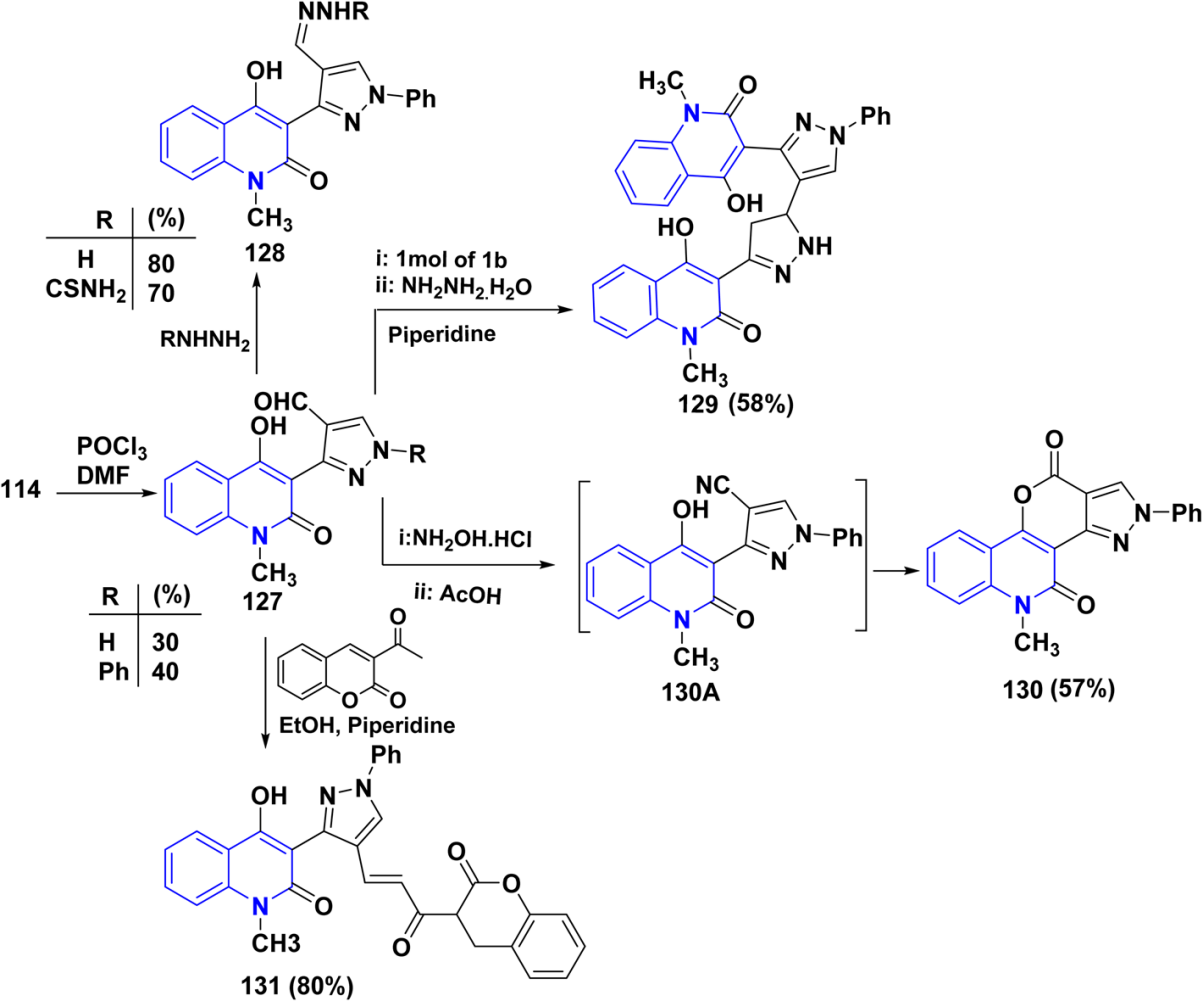
Synthesis of functionalized quinolone derivatives 128–131.

For the synthesis of pyrazole-4-carbaldehydes 127, the hydrazones 114 were exposed to Vilsmeier–Haack reagent (DMF-POCl_3_).^177,178^ While traditional condensation of pyrazole-4-carbaldehydes 127 with hydrazine hydrate or thiosemicarbazide furnished hydrazone derivatives 128 in acceptable yields. Another system in this area was created in two steps. Condensing acetylquinolinone 12b with aldehyde 127 (R = Ph) furnished the *α*, *β*-unsaturated ketone, which was then treated with NH_2_NH_2_, affording polynuclear heterocyclic skeleton 129 (Scheme 46).

Unexpectedly, the condensation of 127 (R = Ph) with NH_2_OH·HCl in AcOH did not produce the predicted oxime or the likely carbonitrile 130A but yielded phenylpyrazolopyrano[3,2-*c*]quinolindione 130, while the oxime transformed to carbonitrile *via* a dehydration process. The carbonitrile 130A was then cycloadditionally converted to an iminopyranoquinolinone, hydrolyzed to yield the isolated tetracyclic system 130. Another option is that the hydrolysis of the intermediate 130A into the equivalent carboxylic acid causes the pyrone ring to form *via* intramolecular condensation. Anyway, both routes are rational and feasible.^178^ Condensation of equimolar amounts of carbaldehyde 127 (R = Ph) with the 3-acetylcoumarin in the presence of piperidine as catalyst afforded the corresponding chalcone 131 (Scheme 46).^177^

**Scheme 47:**
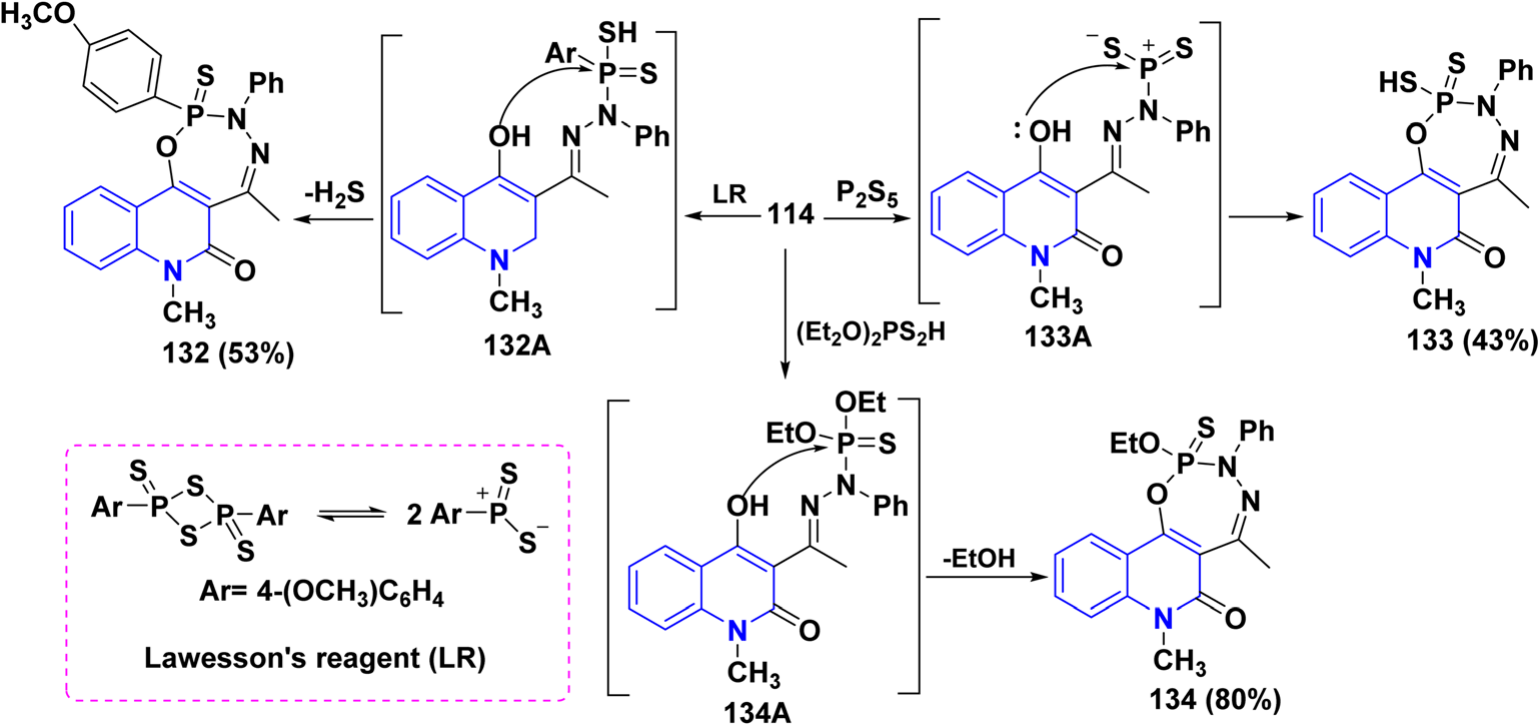
Reaction of hydrazone 114 with sulfur-containing phosphorus reagents.

Additionally, the synthesis of fused phosphorus heterocycles with quinolinone moieties was achieved through an efficient protocol *via* the reaction of hydrazone 114 with some phosphorus reagents. As the treatment of hydrazone 114 with Lawesson's reagent (LR) [which forms two reactive dithiophosphine ylides (R–PS_2_)], phosphorus pentasulfide (P_2_S_5_) and diethyldithiophosphoric acid in refluxing dioxane afforded the corresponding fused tricyclic oxadiazaphosphepino[6,7-*c*]quinolinones 132–134 (Scheme 47).^179^

**Scheme 48:**
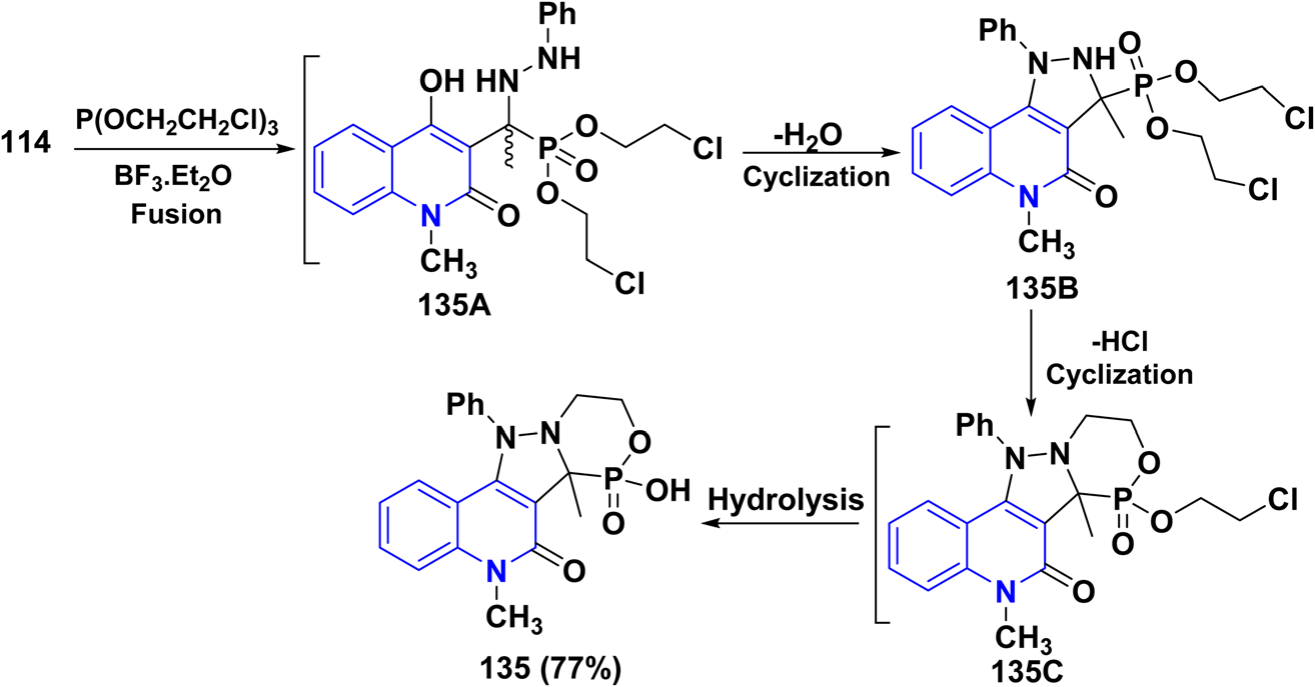
Treatment of hydrazone 114 with P(OCH_2_CH_2_Cl)_3_.

Synthesis of cyclic phosphonic ester 135 was accomplished through treatment of hydrazone 114 with tris(2-chloroethyl)phosphite P(OCH_2_CH_2_Cl)_3_. The credible mechanism for synthesis of compound 135 starting with Michael addition of the phosphite to the azomethine bond to produce the non-isolatable intermediate 135A, which undergoes cyclization through nucleophilic attack of NH functionality at position 4 of the quinoline ring to remove a water molecule yielding the pyrazolyl non-isolatable intermediate 135B which underwent cyclization through elimination of HCl, followed by acidic hydrolysis yielding 135 as final product (Scheme 48).^179^

**Scheme 49:**
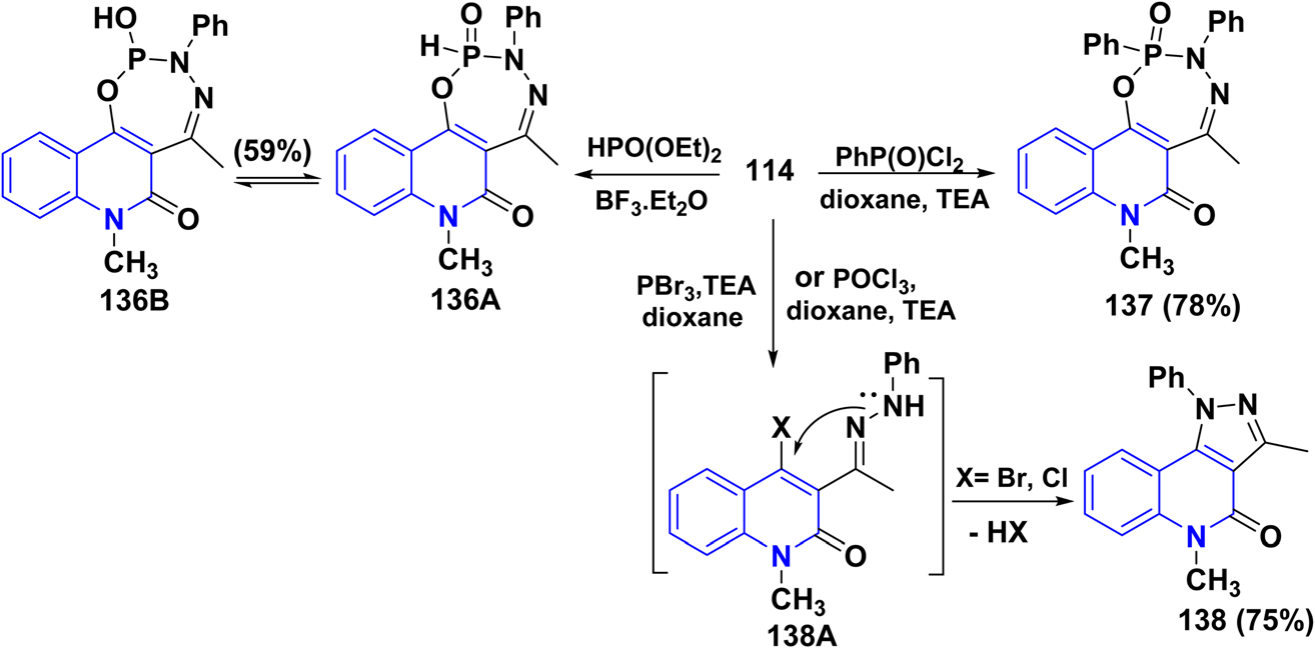
Annulation of hydrazone 114 with diethyl phosphite and phosphohalogenating agents.

Furthermore, Pudovik reaction of hydrazone 114 with diethyl phosphite in the presence of BF_3_ etherate at 80–90 °C furnished oxadiazophosphepino[6,7-*c*]quinolinone 136A,B. The plausible mechanism involves a cyclization of hydrazone 114 through the nucleophilic attack of OH and NH groups at the phosphorus atom, followed by stripping off two molecules of EtOH. Additionally, heterocyclization of hydrazone 114 with phenylphosphonic dichloride (C_6_H_5_Cl_2_PO) in dioxane afforded the corresponding oxadiazaphosphepinoquinolinone 137(Scheme 49).^179^

Finally, reaction of hydrazone 114 with equimolar amounts of with diethyl phosphite, PhPOCl_2_, phosphorus tribromide (PBr_3_) or POCl_3_ in dry dioxane in molar ratio (1 : 1) containing catalytic amount of TEA afforded 138, The proposed mechanism for the synthesis of fused tricyclic 138 may be attributed to halogenation of OH group process through the at C_4_. A phosphorus halide of the quinolinone ring, cascade by cyclization of intermediate 138A through dehydrohalogenation elimination of HX using TEA yielding 138 (Scheme 49).^179^

##### 4.2.1.3. With hydroxylamine

**Scheme 50:**
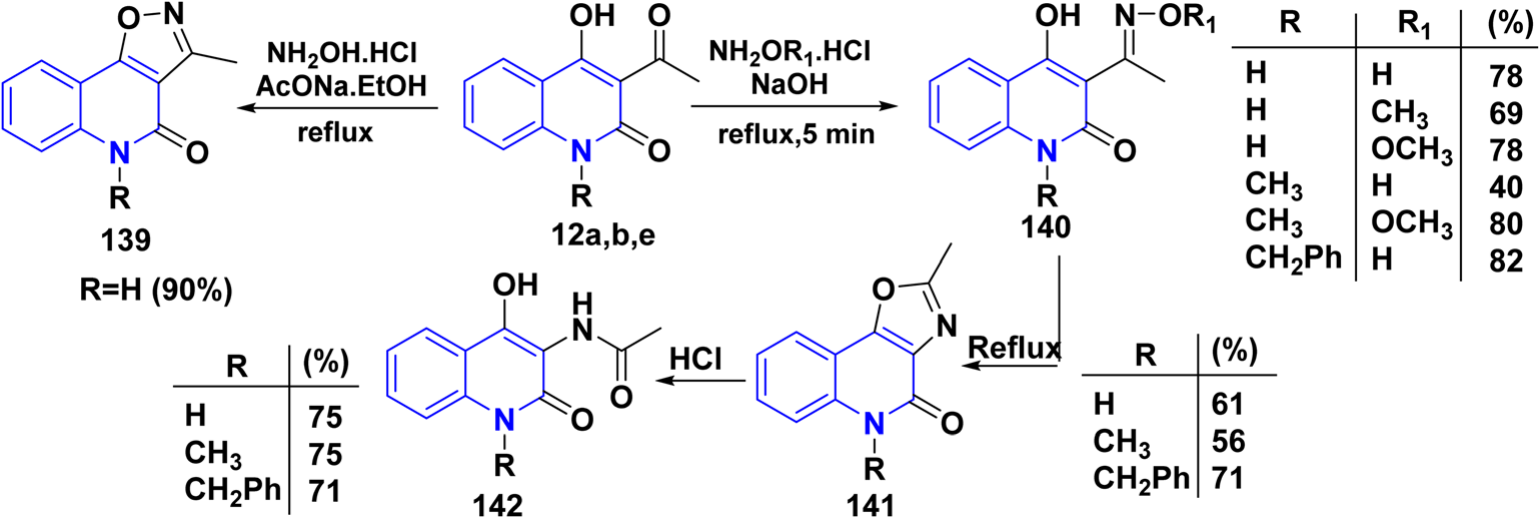
Condensation and thermal Beckmann rearrangement of oxime product.

Reaction of acetylquinolinone 12a with NH_2_OH·HCl was accomplished in an ethanolic solution in the presence of AcONa furnished the corresponding oxazole 139. Whereas, repeating the reaction in the presence of NaOH instead of AcONa furnished corresponding oxime 140 (Scheme 50).^115,118,134,145,172,174^

3-Oximatoacyl-4-hydroxyquinolones 140 were synthesized by reacting AHQ 12a,b,e with excess hydroxylammonium chloride and hydrogencarbonate, yielding hydroxyiminoquinolones 140 (R_1_ = H). To obtain the oximethers 140 (R_1_ = methyl), quinolinone 12a,b were reacted with methyloxyammonium chloride and hydrogencarbonate. Both types of compounds 140 structurally resemble biologically active compounds like alloxydim or sethoxy. When oximes are thermolyzed according to Beckmann rearrangement and converted into isomeric oxazolo[5,4-*c*]quinolinones 141 in high-boiling solvents like 1,2-dichlorobenzene and diphenyl ether. Whereby, the hydrolysis process of oxazole derivatives 141 with dil. HCl led to formation of 3-acylamino-4-hydroxyquinolones 142 (Scheme 50).^172^

#### 4.2.2. With phenylisothiocyante

**Scheme 51:**
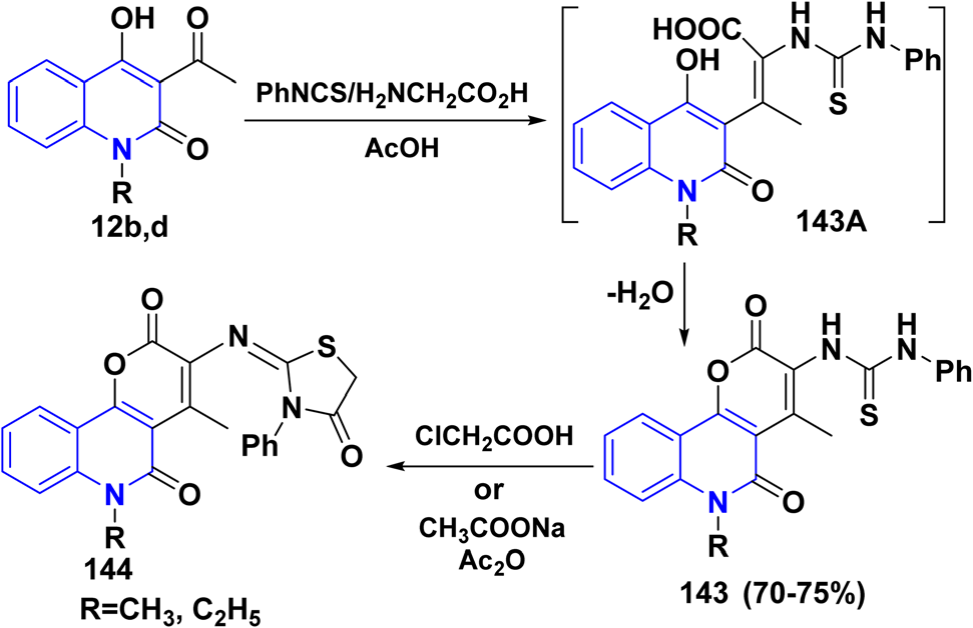
Formation of thiazolidine derivatives 144.

Multicomponent reaction (MCR) of 12b,d, phenylisothiocyanate and glycine furnished *N*-pyrano[3,2-*c*]quinolinedion phenylthiourea 143 in acceptable yields *via* condensation of the carbonyl group of 12b,d with the active methylene group in glycine to yield the intermediates 143A which readily cyclized through stripping off water molecule to afford 143. Whereas, the condensation of 143 with chloroacetic acid and Ac_2_O afforded thiazolidine derivatives 144 (Scheme 51).^151^

### 4.3. Condensation reactions

#### 4.3.1. With aldehydes (synthesis of *α*,*β*-unsaturated ketones)

**Scheme 52:**
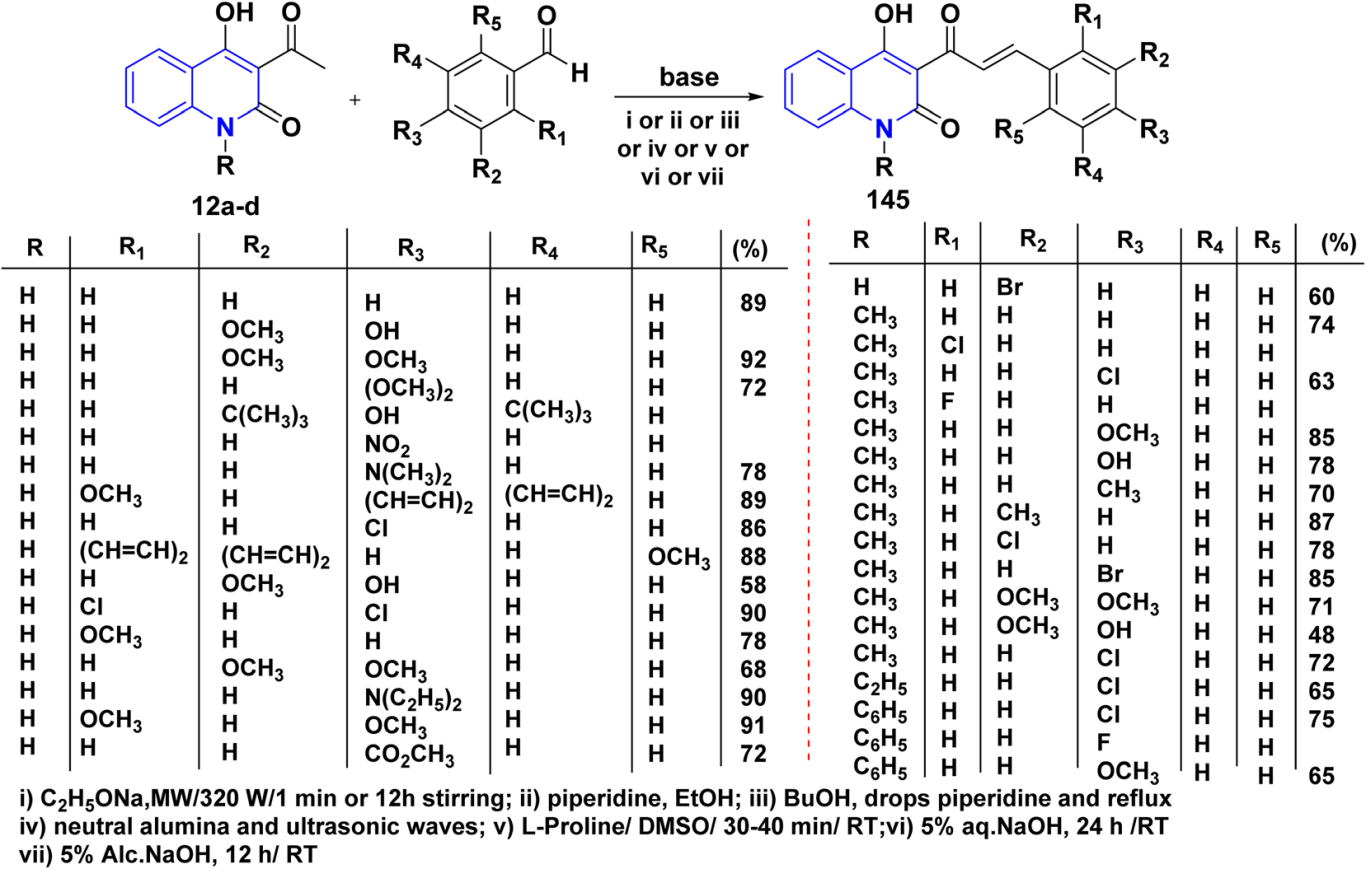
Synthesis of *α*,*β*-unsaturated ketones 145.

**Scheme 53:**
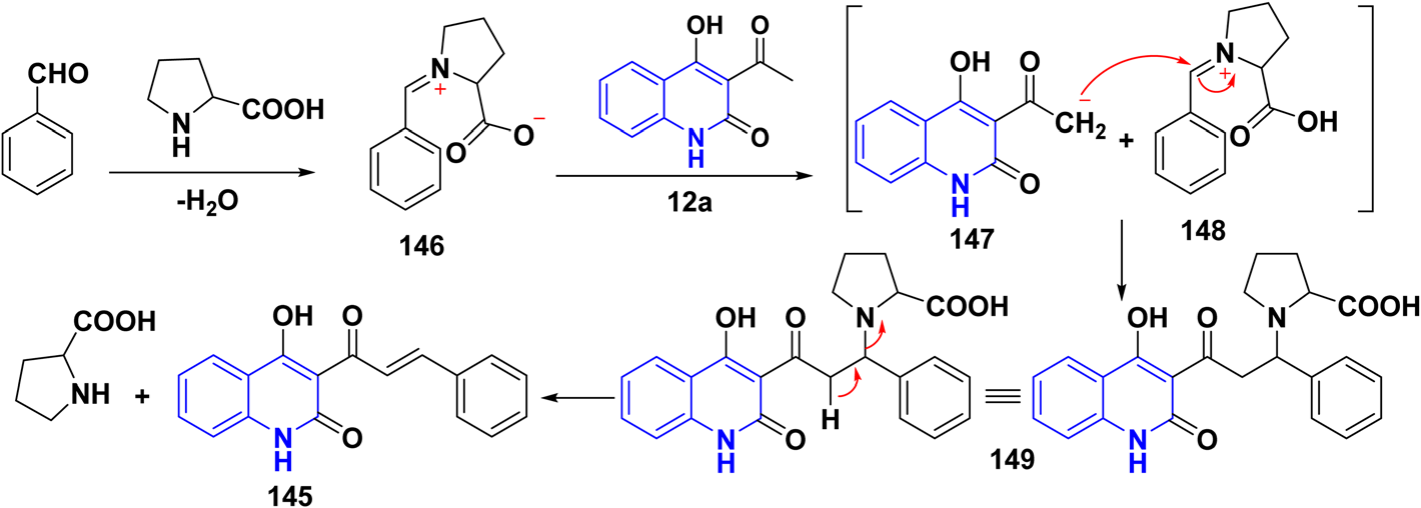
Proposed mechanism for the synthesis of *α*,*β*-unsaturated ketone 145 in the presence of l-proline.

A series of *α*,*β*-unsaturated ketones of quinolinone analogues 145 were synthesized through Claisen–Schmidt condensation of 12a–d with aldehydes under reflux or microwave irradiation.^115,131,146,180–184^ The mechanism of the reaction started by reacting of formyl group with l-proline to form an iminium carboxylate ion 146, after that this ion eliminating a proton from active methylene of 12a affording the carboxylic acid containing iminium ion 148 and quinoline anion 147 as ion pairs that combine to form the adduct 149 which loses proline to afford α,*β*-unsaturated ketone 145 (Schemes 52 and 53).^183^

**Scheme 54:**
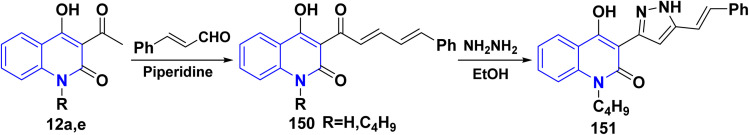
Reaction of quinolinone 12a,e with cinnamaldehyde.

Similarly, reaction of quinolone 12a,e with cinnamaldehyde in the presence of piperidine in refluxing EtOH afforded *α*,*β*-unsaturated ketone 150. After that, treatment of 150 with hydrazine hydrate led to the formation of styrylpyrazolylquinolinone 151 (Scheme 54).^146,185^

**Scheme 55:**
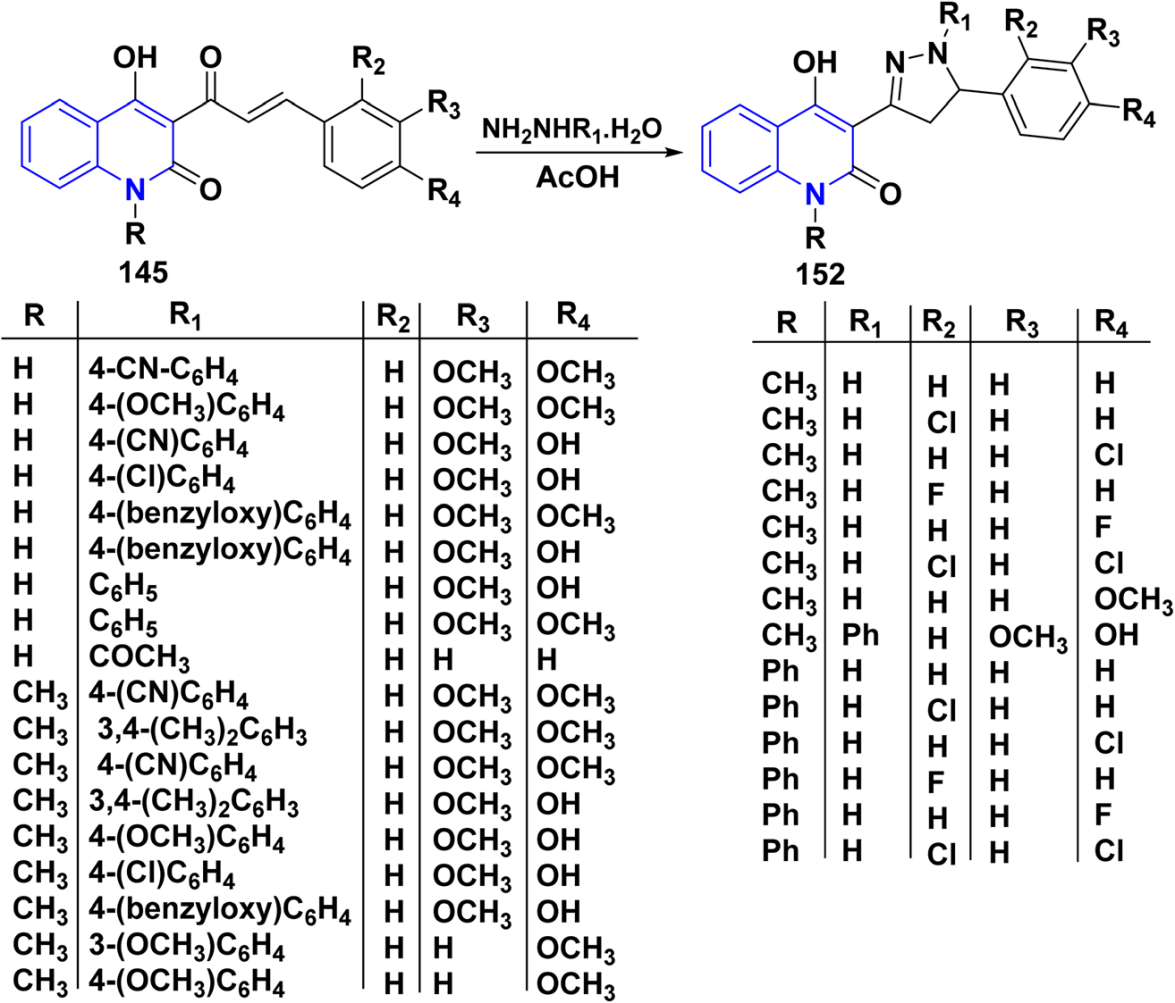
Synthesis of pyrazolylquinolinones through the reaction of 145 with hydrazines.

The predominant transformations for generating pyrazole rings typically involve the use of *α*,*β*-unsaturated carbonyl group derivatives as parent skeletons. Chalcones 145 were treated with appropriate hydrazines in AcOH, furnished functionalized pyrazoline analogues 152 (Scheme 55).^108,146,184,186^

**Scheme 56:**
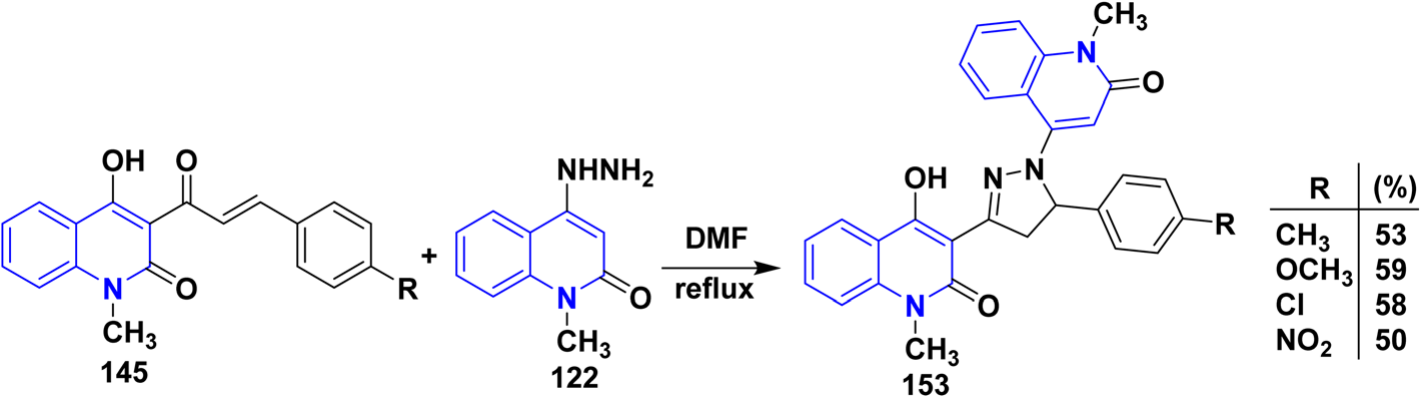
Synthesis of binary pyrazolylquinolinone derivatives.

Whereby, the hydrazinoquinolinone 122 underwent condensation followed by cycloaddition reaction with *α*,*β*-unsaturated ketone derivatives 145, affording binary quinolinylpyrazoloquinolinone derivatives 153 up to yield 59% (Scheme 56).^176^

**Scheme 57:**
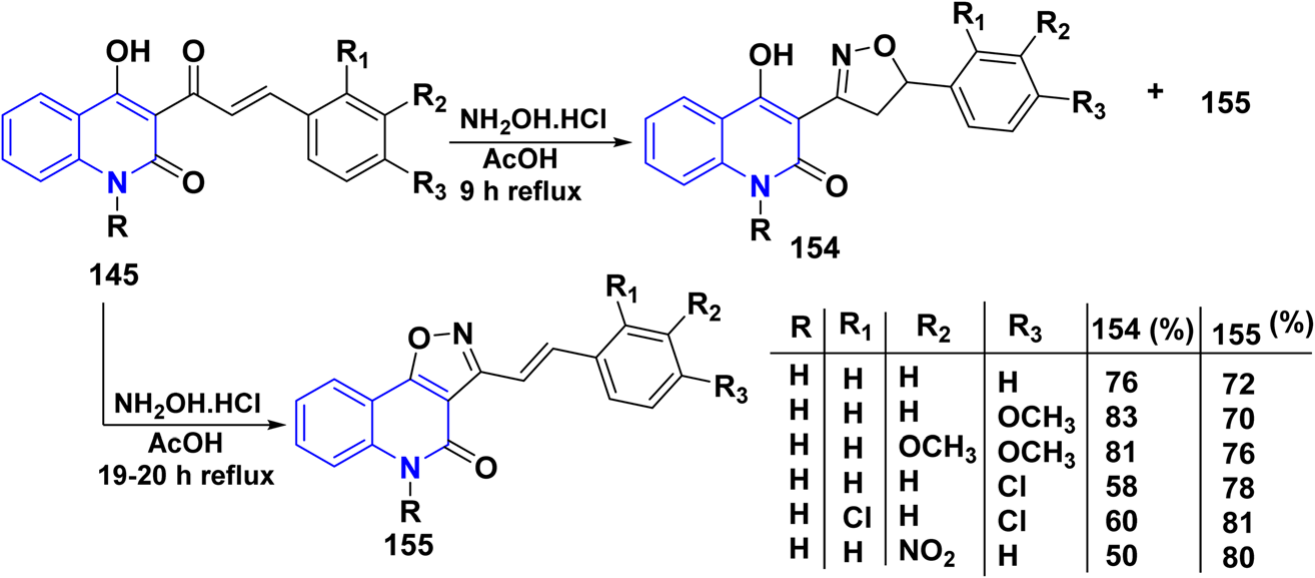
Synthesis of fused and binary isoxazolo and isoxazolyl quinolinone 154 and 155.

Whereas, the reaction of 145 with NH_2_OH·HCl in glacial AcOH yielded a mixture of two products 154 and 155. Conversely, repeating the same reaction in other solvents such as benzene, MeOH, and EtOH was unsuccessful. Since the isoxazolo[4,5-*c*]quinolin-4(5*H*)-ones 155 formation could not be controlled to get only isoxazolines 154, increasing the reaction duration to 19–20 h led to isoxazoles 155 as major products, and the minor products could not be isolated (Scheme 57).^180^

**Scheme 58:**
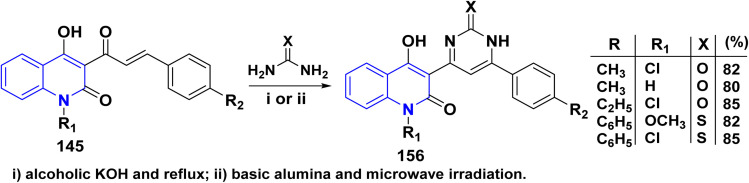
Synthesis of pyrimidinylquinolinone 156.

Pyrimidinylquinolinone quinolinyl pyrimidine derivatives 156 have been synthesized through both traditional methods and microwave irradiation. These compounds were obtained *via* condensing *α*,*β*-unsaturated ketone derivatives 145 with urea or thiourea in basic media (Scheme 58).^182^

**Scheme 59:**
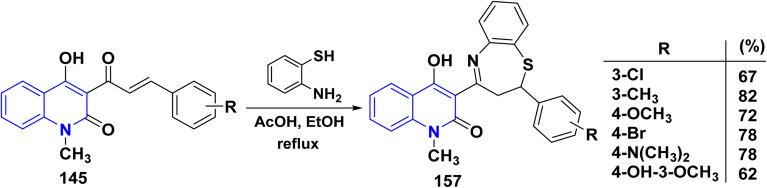
Synthesis of 1,5-benzothiazepin ylquinolinones 157 anchored on quinolone.

Furthermore, substituted 1,5-benzothiazepinylquinolinones 157 were synthesized by cyclocondensation reaction followed by Michael addition. Whereas, a mixture of *α*,*β*-unsaturated ketone 145, *o*-aminothiophenol in absolute EtOH then added glacial AcOH (5 mol%). The obtained reaction mixture was refluxed for 5–7 h, furnishing the target product 1,5-benzothiazepinylquinolinones 157 in yields 62–82% (by the optimized condition) (Scheme 59).^133^

**Scheme 60:**
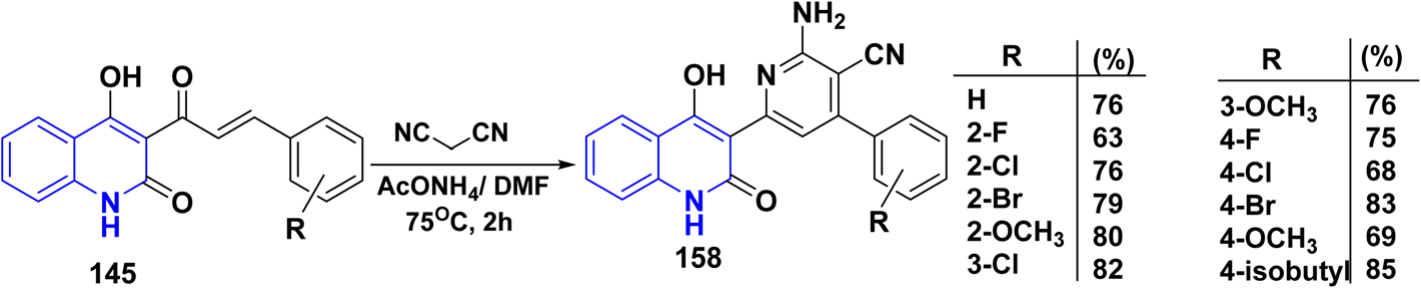
Synthesis of 4-hydroxy-oxoquinolin-4-arylpyridine-3-carbonitriles 158.

MCRs of *α*,*β*-unsaturated ketone 145, malononitrile, and ammonium acetate in DMF at 75 °C afforded functionalized pyridine binary with quinoline systems 158 (Scheme 60).^187^

**Scheme 61:**
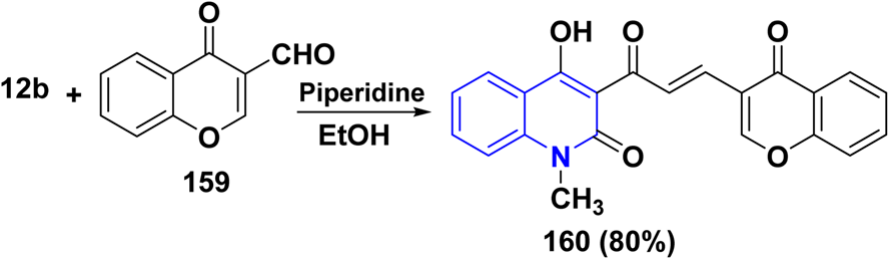
Reaction of quinolinone 12b with 3-formylchromone 159.

The desired precursor chromenacryloylquinolinone 160 was obtained smoothly *via* a one-pot Aldol condensation dehydration reaction of 12b with 3-formylchromone 159 (Scheme 61).^188^

In refluxing EtOH, the treatment of compound 160 with an equimolar amount of hydrazine hydrate led to the opening of the pyrone ring and the enone side chain unaffected, affording a new product assigned as 4-hydroxy-3-[3-(2-hydroxyphenyl)pyrazolyl]acryloylg quinolineone 161. Whereas, using AcOH as the solvent oriented the reaction away from the γ-pyrone nucleus, yielding 3-[1-acetyl-5-(4-oxo-4*H*-chromen-3-yl)-4,5dihydropyrazolyl]-4-hydroxyquinolinone 162.

**Scheme 62:**
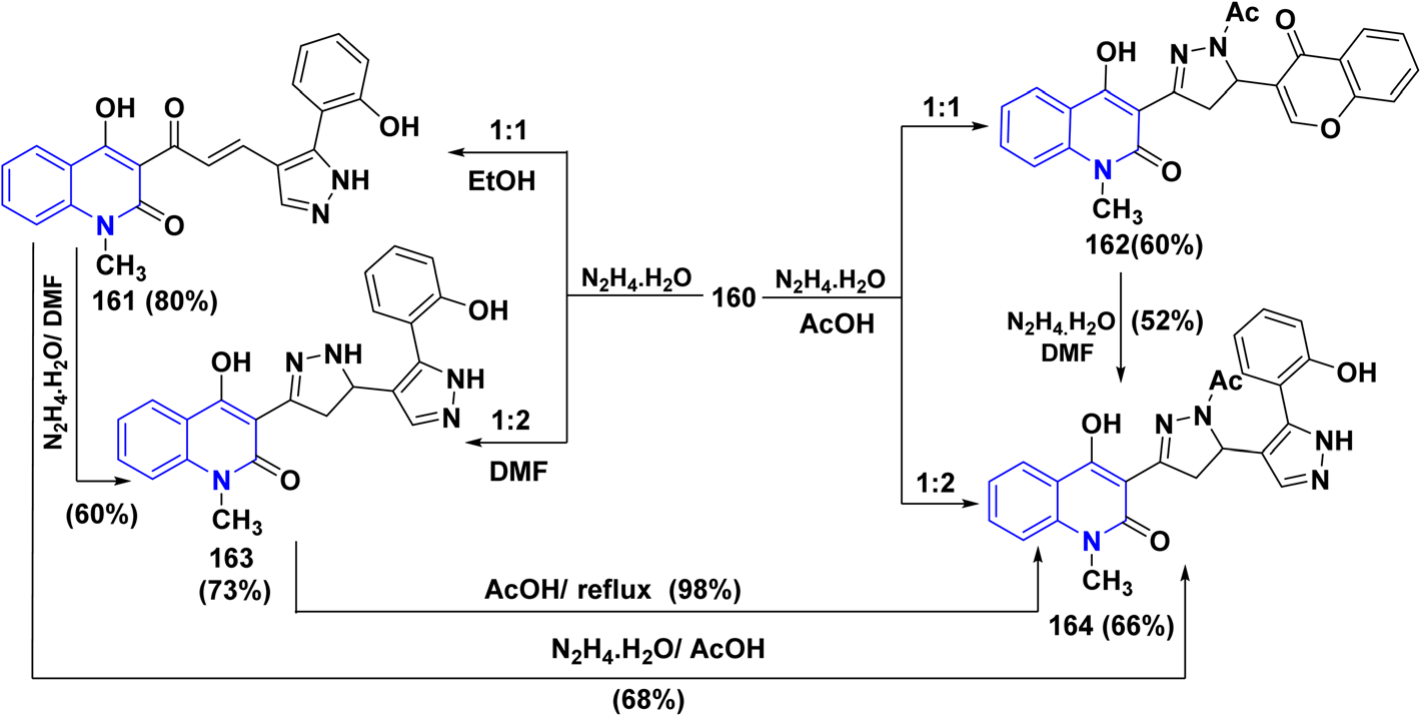
Synthetic pathway to generate pyrazolo and bipyrazoloquinolone.

While by, compound 160 was reacted with of hydrazine hydrate (1 : 2) in refluxing DMF affording 4-hydroxy-3-[30-(2-hydroxyphenyl)bipyrazolyl]quinolinone 163 in 73% yield, through double nucleophilic attack at both the *α*,*β*-unsaturated ketone side chain and chromone ring, where by, the same product was prepared by reacting the pyrazolylacryloylquinolinone 161 with an excess amount of hydrazine hydrate in boiling DMF in 60% yield. Interestingly, the reaction of compound 160 with hydrazine (1 : 2) in AcOH did not give either compound 162 or compound 163 but yielded the *N*-acetyl derivative of compound 163 and formulated as binary bipyrazoles 164. Annotative synthesis of 164 was preceded by reaction of 161 with NH_2_NH_2_ in AcOH or treatment of compound 162 with NH_2_NH_2_ in boiling DMF (Scheme 62).^188^

**Scheme 63:**
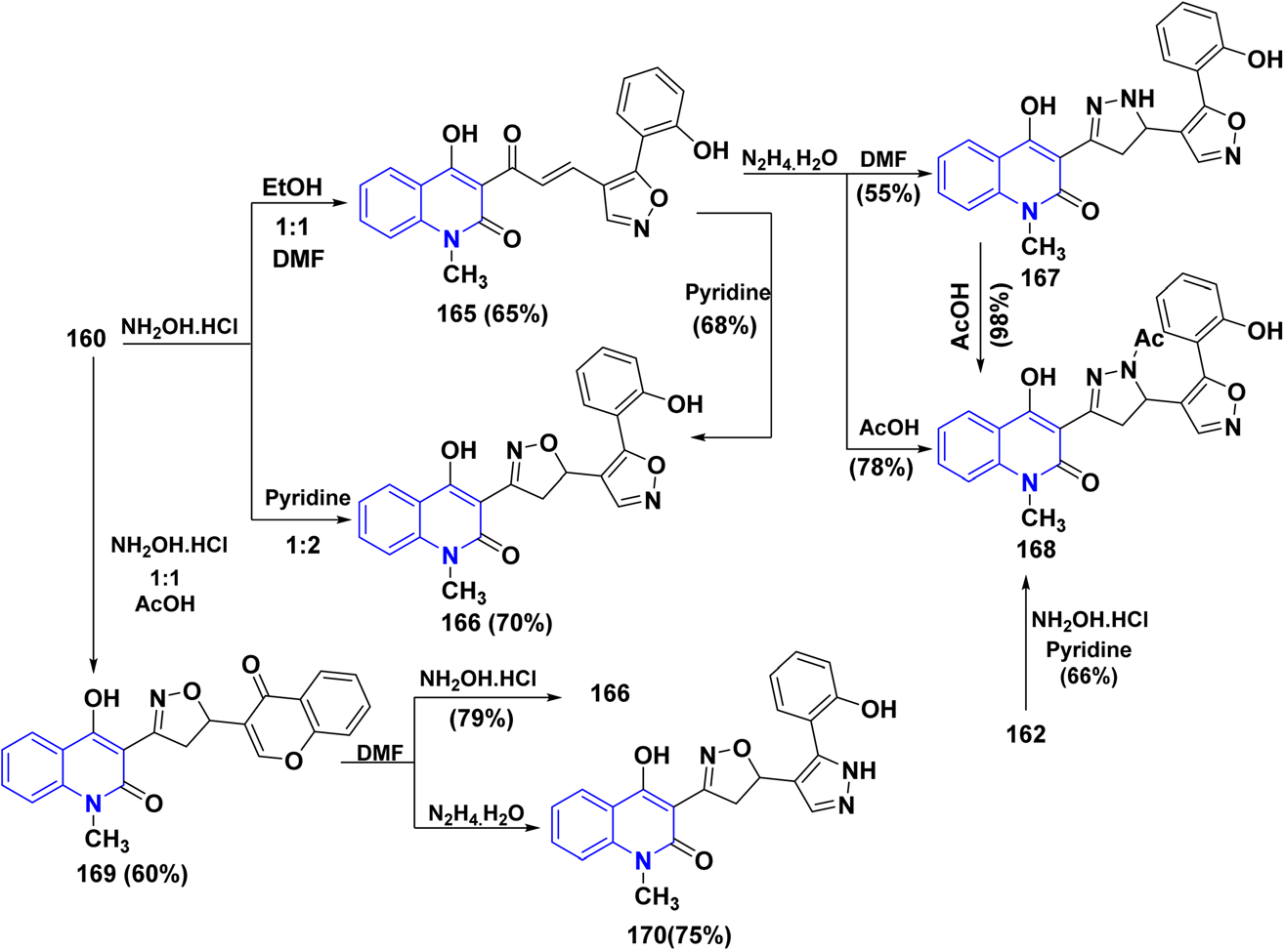
Reaction of 160 with NH_2_OH·HCl in different conditions.

Reaction of equimolar amounts of 160 with NH_2_OH·HCl in a mixed solvent of ethanol–DMF led to the formation of 4-hydroxy[5-(2-hydroxyphenyl)isoxazolyl]acryloylgquinolinone 165. Additionally, the reaction of both compounds 165 with NH_2_OH·HCl in boiling DMF or pyridine afforded 4-hydroxy-3-[5-(2-hydroxypheny1)biisoxazoly1]quinolinone 166, which is obtained directly from 160, when using an excess of NH_2_OH·HCl (1 : 2) in refluxing pyridine. Additionally, polycyclic skeleton 167 was obtained upon reacting isoxazolylacryloy1quinolinone 165 with NH_2_NH_2_ in boiling DMF. Moreover, boiling compound 167 in AcOH led to acetylation, yielding acetyl-5-[5-(2-hydroxypheny1)isoxazol-4-y1]pyrazol-3-y1-4-hydoxyquinolinone 168, a compound that was also obtained during heterocyclization of the acryloy1 derivative 165 by means of hydrazine hydrate in AcOH. In the same line, the reaction of *N*-acetylpyrazoline 162 with NH_2_OH·HCl furnished the same compound 168 (Scheme 63).

Whereas, the reaction of 160 with an equimolar amount of NH_2_OH·HCl in AcOH proceeds in a different manner, and afforded 4-hydroxy-1-methy1-3-[5-(4-oxochromeny1)isoxazol-3-yl)quinolinone] 169. After that, the reaction of chromeny1isoxazoliny1quinolinone 169 with NH_2_OH·HCl in boiling DMF afforded 4-hydroxy-3-[5-(2-hydroxypheny1)biisoxazol-y1]quinolinone 166. Also, chromeny1isoxazoliny1quinolinone 169 was treated with NH_2_NH_2_ in boiling DMF yielded 4-hydroxy[3-(2-hydroxy phenyl) -pyrazol-4-yl]isoxazol-3-y1-quinolinone 170 (Scheme 63).^188^

**Scheme 64:**
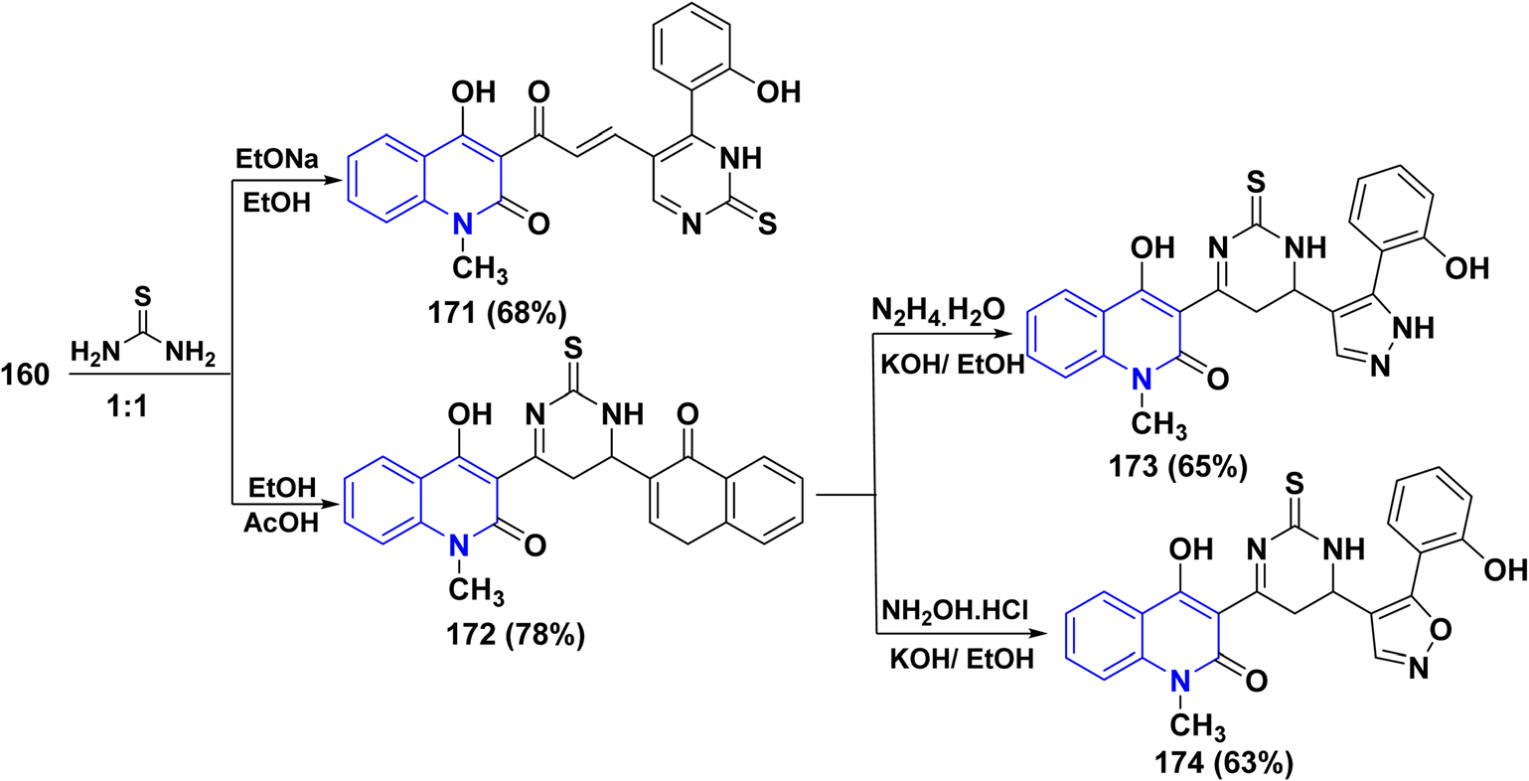
Synthetic pathway to generate pyrimidine, pyrazole and oxazole scaffolds.

Equimolar ratio of 160 and thiourea was refluxed in the presence of sodium ethoxide, affording thioxopyrimidinquinolin-2-one 171 under various reaction conditions. In refluxing EtOH containing a catalytic amount of HCl or in refluxing AcOH, the same reaction resulted in the formation of chromenthioxopyrimidinquinolinone 172. The reaction of compound 172 with both hydrazine hydrate and NH_2_OH·HCl proceeded smoothly using ethanolic potassium hydroxide to afford pyrazolyl 173 and its isoxazolyl analogue 174, respectively (Scheme 64).^188^

**Scheme 65:**
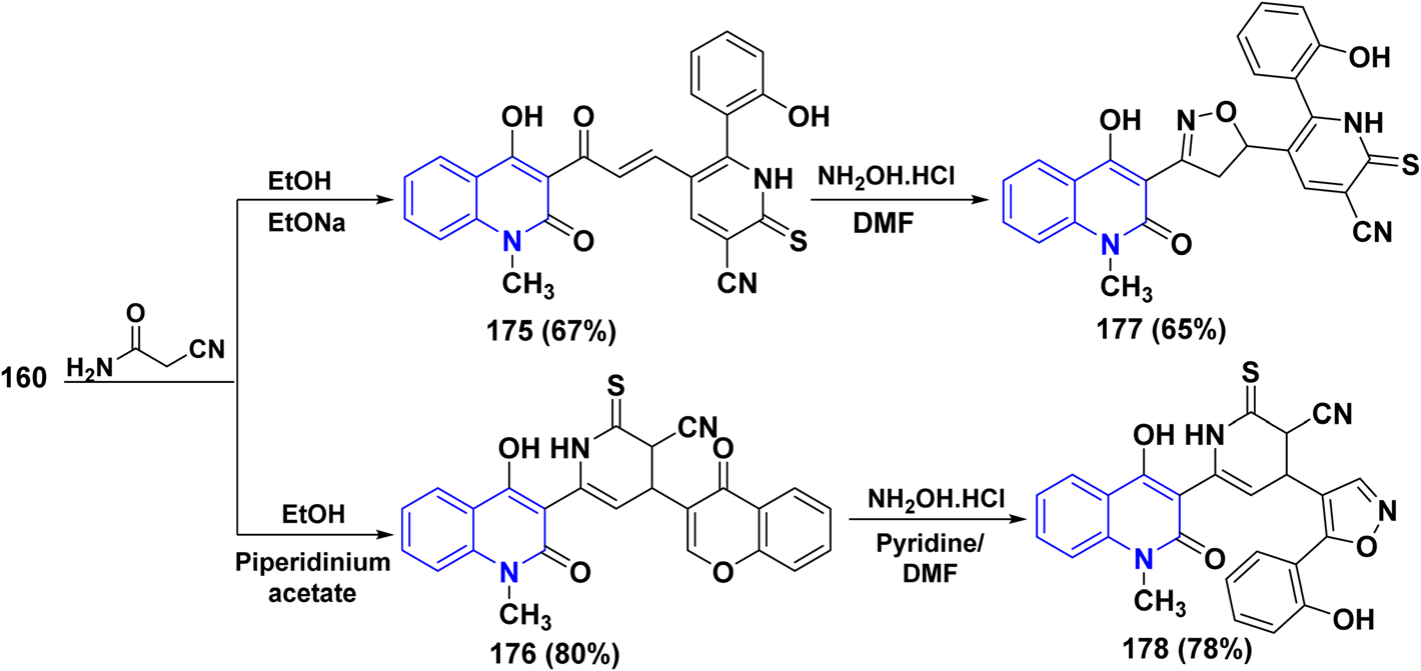
Reaction of 160 with cyano thioacetamide in different reaction conditions.

2-Thioxopyridine-3-carbonitriles have great interest due to their usage as intermediates for the synthesis of various medicines. Thus, synthesis of 4-hydroxy-1-methyl-2-oxoquinolin thioxo-3-carbonitrile 175 was achieved by reaction of an equimolar amount of cyano thioacetamide with compound 160, using sodium ethoxide as a catalyst. Whereas, in a relatively moderate basic medium using piperidinium acetate, the reaction approaching another possible product accessible by a Michael route led to the formation of 6-(4-hydroxy-2-oxo-1,2-dihydroquinolinyl)-4-(4-oxo-4*H*-chromen-3-yl)-2-thioxopyridine-3-carbonitrile 176. The heterocyclization reaction of both compounds 175 and 176 with NH_2_OH·HCl, in boiling pyridine and/or DMF, yielded two important triheterocyclic systems: 5-[3-(4-hydroxy-2-oxo-quinolin-3-yl)-4,5-dihydro-isoxzolyl]-6-(2-hydroxyphenyl)-2-thioxopyridine-3-carbonitrile 177 and 6-(4-hydroxyl-2-oxoquinolin-3-yl)-4-[5-(2-hydroxyphenyl)-isoxazolyl]-2-thioxopyridine-3-carbonitnile 178 (Scheme 65).^188^

2-Thiobarbituric acid is recognized as a reactive cyclic methylene compound of the 1,3-dione type, which is utilized to synthesize pyranopyrimidines when cyclized with enone systems or 1,3-dicarbonyl compounds, and more recently with 3-substituted chromones.^189^ When compound 160 was treated with 2-thiobarbituric acid in an equimolar ratio in the presence of sodium ethoxide, it yielded (4-hydroxy-2-oxoquinolin-3-yl)-3-oxopropenyl]thioxopyrano[2,3-*d*]pyrimidinone 179.

**Scheme 66:**
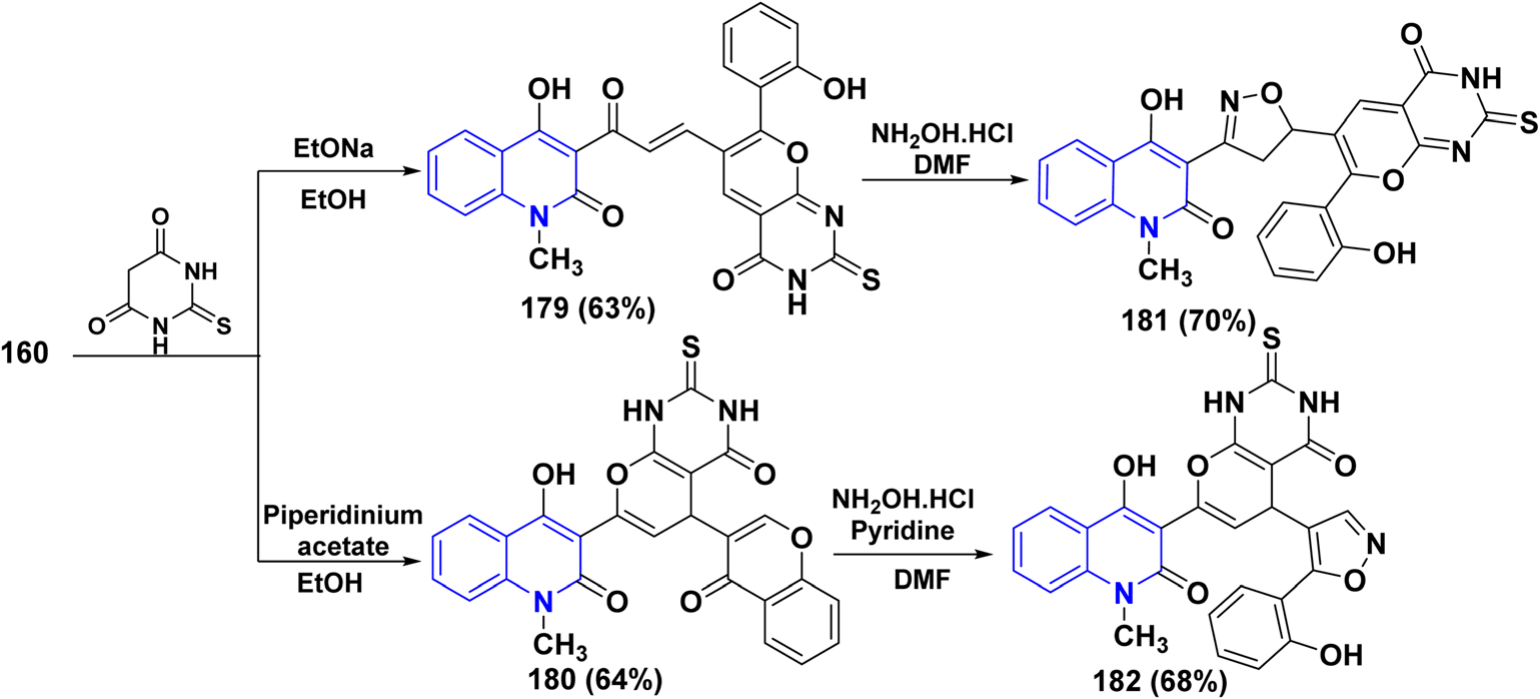
Reaction of 160 with thiobarbituric acid in different reaction conditions.

While by, the same reaction occurred in the presence of piperidinium acetate, a polycyclic molecule, thioxopyrano[2,3-*d*]pyrimidinone derivative 180 was obtained. Furthermore, both 179 and 180 were reacted with NH_2_OH·HCl in DMF affording the [3-(4-hydroxy-2-oxoquinolin-3-yl)isoxazol-5-yl]-7-(2-hydroxyphenyl)-2-thioxopyrano[2,3-*d*]-pyrimidinone 181 and (4-hydroxy-2-oxo-quinolin-3-yl)-5-[5-(2-hydroxyphenyl)isoxazol-4-yl]-2-thioxopyrano[2,3-d]pyrimidinone 182, respectively (Scheme 66).^188^

#### 4.3.2. With DMF/DMA

*N*,*N*-Dimethylformamide dimethyl acetal (DMFDMA), also known as 1,1-dimethoxy-*N*,*N*-dimethylmethanamine, The chemical structure of DMF/DMA has two significant sites: a partially positive carbon that functions as an electrophile in condensation processes and a partially negative nitrogen that acts as a nucleophile. Owing to its structure, DMF/DMA reacts with a wide range of organic groups and can be a useful reagent as a C1 synthon, particularly in the production of heterocycles. Also, it is used to formylate aromatic compounds. The process involves initial conversion of DMF to a chloroiminium ion, [(CH_3_)_2_N=CH(Cl)]^+^, known as a Vilsmeier reagent, which attacks arenes. Thermal condensation of 12b,d with DMF/DMA, afforded the corresponding dimethylaminoacryloylquinolinone derivatives as (enaminone compound) 183.^190^ Reaction of enaminone 183 with 4-aminoantipyrine, furnished 3-(3-((1,5-dimethyl-3-oxo-2-phenyl-2,3-dihydro-1*H*-pyrazol-4-yl)amino)acryloyl)-4-hydroxyquinolinone 184. Whereas, the reaction of the enaminone 183 with morpholine and piperidine as cycloaliphatic secondary amines smoothly proceeded, leading to the corresponding 4-hydroxy-1-alkyl-3-(3-morpholino/piperidinoacryloyl)quinolinone 185.

While by, treating enaminone with hippuric acid (*N*-benzoylglycine) or aceturic acid (*N*-acetylglycine) 186 in Ac_2_O resulted in the production of 187. It is suggested that this process begins with the cyclization of 186 into oxazolone, which then adds to the activated double bond system of enaminone 183, followed by further spontaneous rearrangement yielding 187.^177,191^

**Scheme 67:**
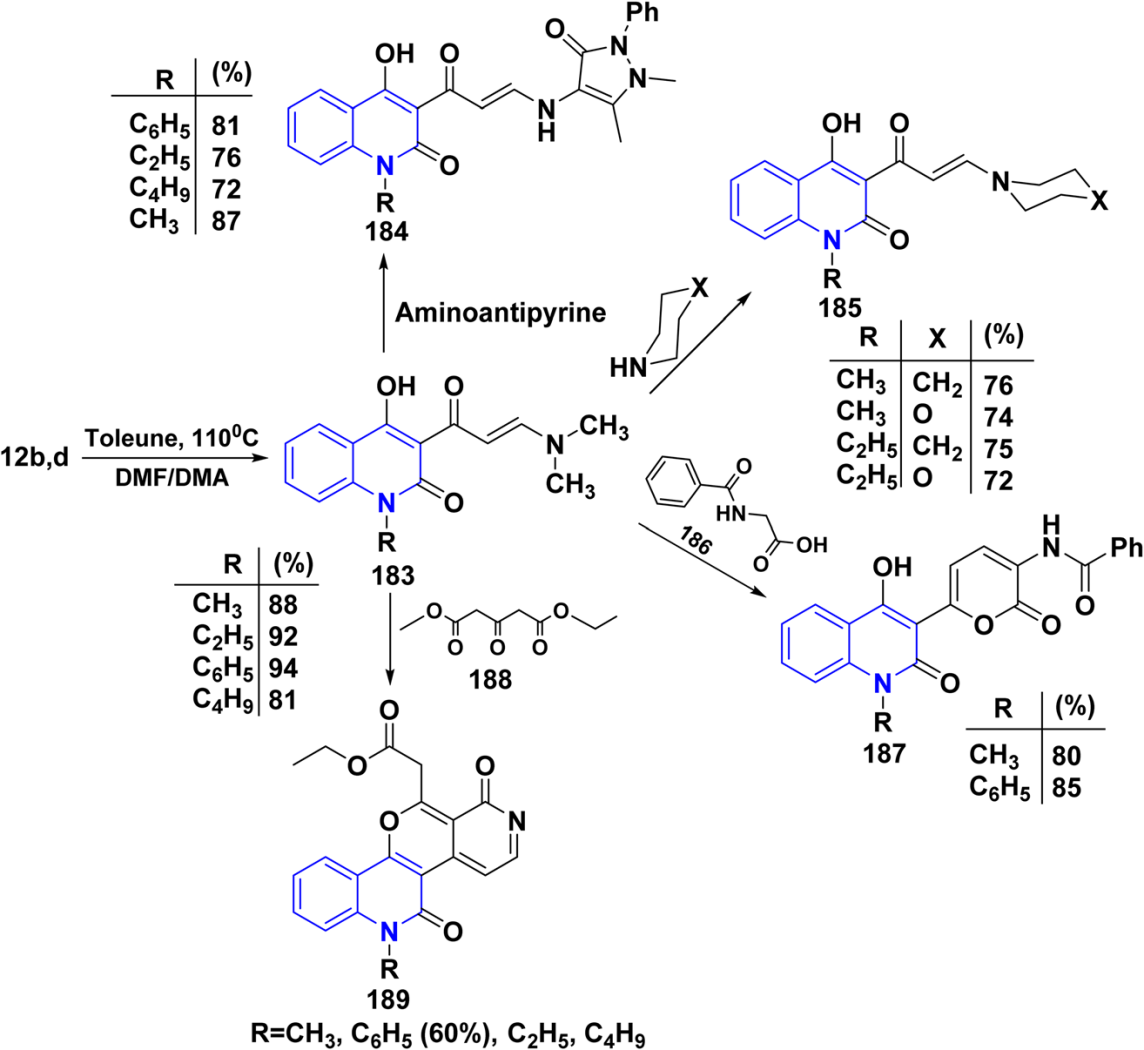
Reaction series of enaminone 183 with different heterocyclic reagents.

Furthermore, compound 189 was synthesized through condensing the active methylene group in diethyl acetonedicarboxylate 188 with the carbonyl group in compound 183, *via* water elimination and the formation of an intermediate 189B. This intermediate is then cyclized in the presence of AcONH_4_, resulting in the stripping of a water molecule yielding angular tetracyclic compound 188 (Scheme 67).^190^

**Scheme 68:**
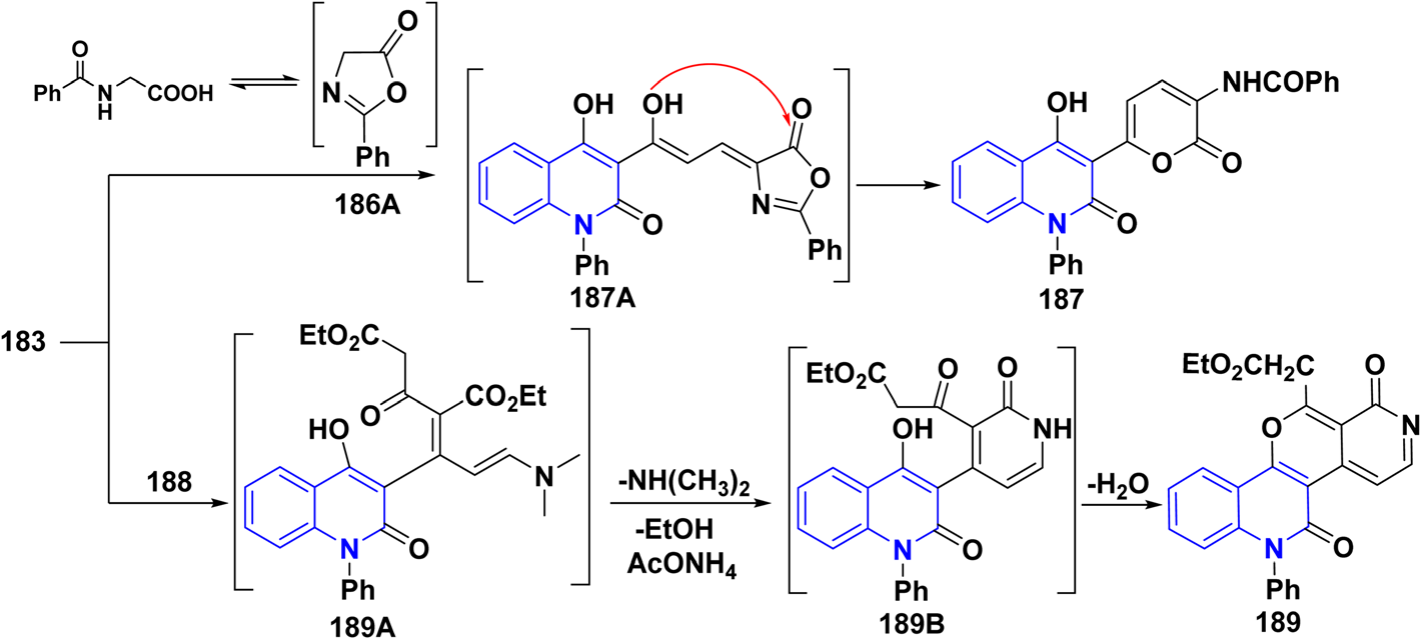
Plausible mechanism for synthesis of compond 187 and 189.

A probable mechanism for the synthesis of 187 started with the cyclization of hippuric acid into oxazolone 186A, which then adds to the activated double bond system of the enaminone 183, yielding 187A, followed by further rearrangement of this intermediate to give 187. Whereas, compound 189 is thought to be formed *via* condensation of the active methylene in 188 with the carbonyl function in 183 with water elimination, forming the intermediate 189A that was cyclized in the presence of ammonium acetate *via* elimination of a molecule of EtOH and water to afford the final product 189 (Scheme 68).

**Scheme 69:**
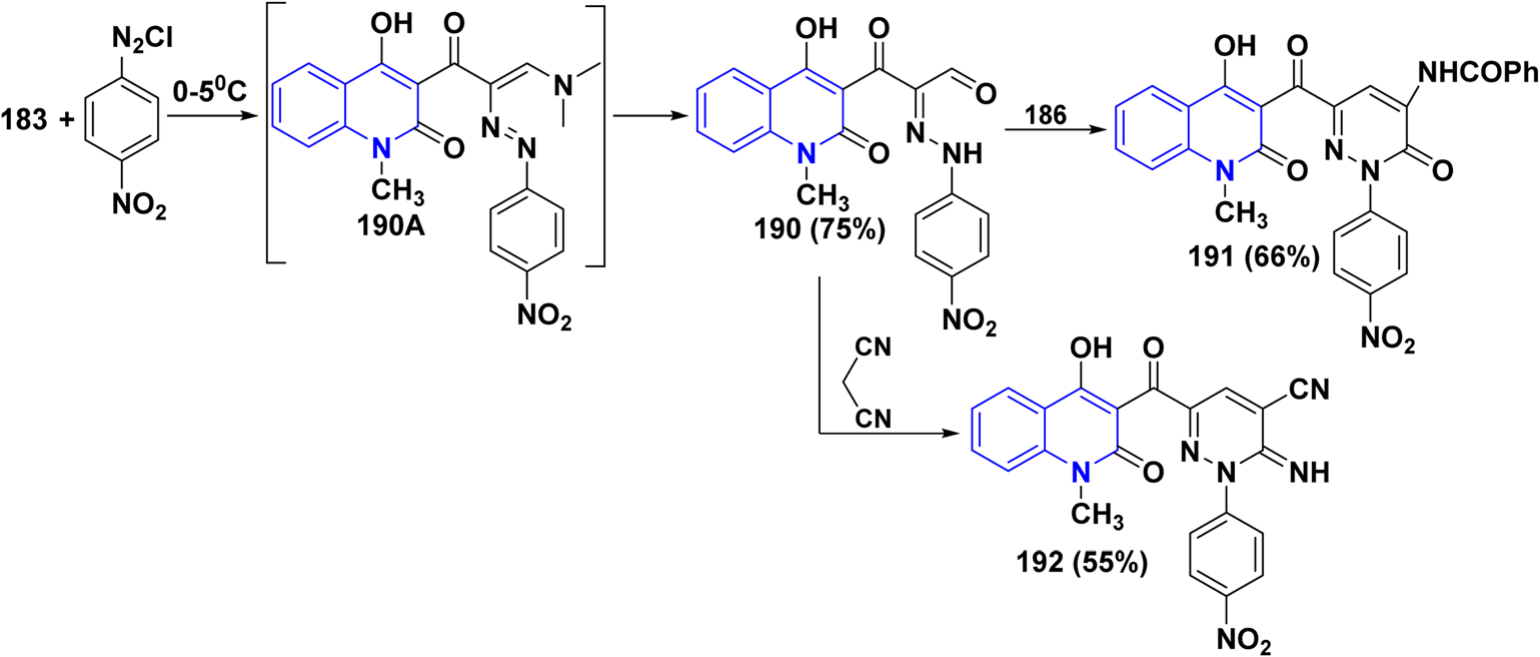
Coupling of enaminone 183 with 4-nitrobenzenediazonium chloride followed by cyclization.

Biological activities of pyridazinone ring systems represent one of the most active classes of heterocycles. Consequently, enaminone 183 was employed as a promising building block for synthesizing pyridazinone 191. Compound 183 coupled with 4-nitrobenzenediazonium chloride, leading to the formation of the arylazo intermediate 190A, which undergoes hydrolysis during the reaction to produce *α*-hydrazono-*β*-oxopropanal 190. Cyclization of the hydrazone derivative 190 with hippuric acid 186 in Ac_2_O produced the 4-benzoylamino-6-[(quinolin-3-yl)carbonyl]-pyridazinone 191 in 66% yield. Whereas, the reaction of compound 183 with malononitrile in absolute ethanol in the presence of piperidine yielded carbonitrile derivative 192 in 55% yield (Scheme 69).^178^

**Scheme 70:**
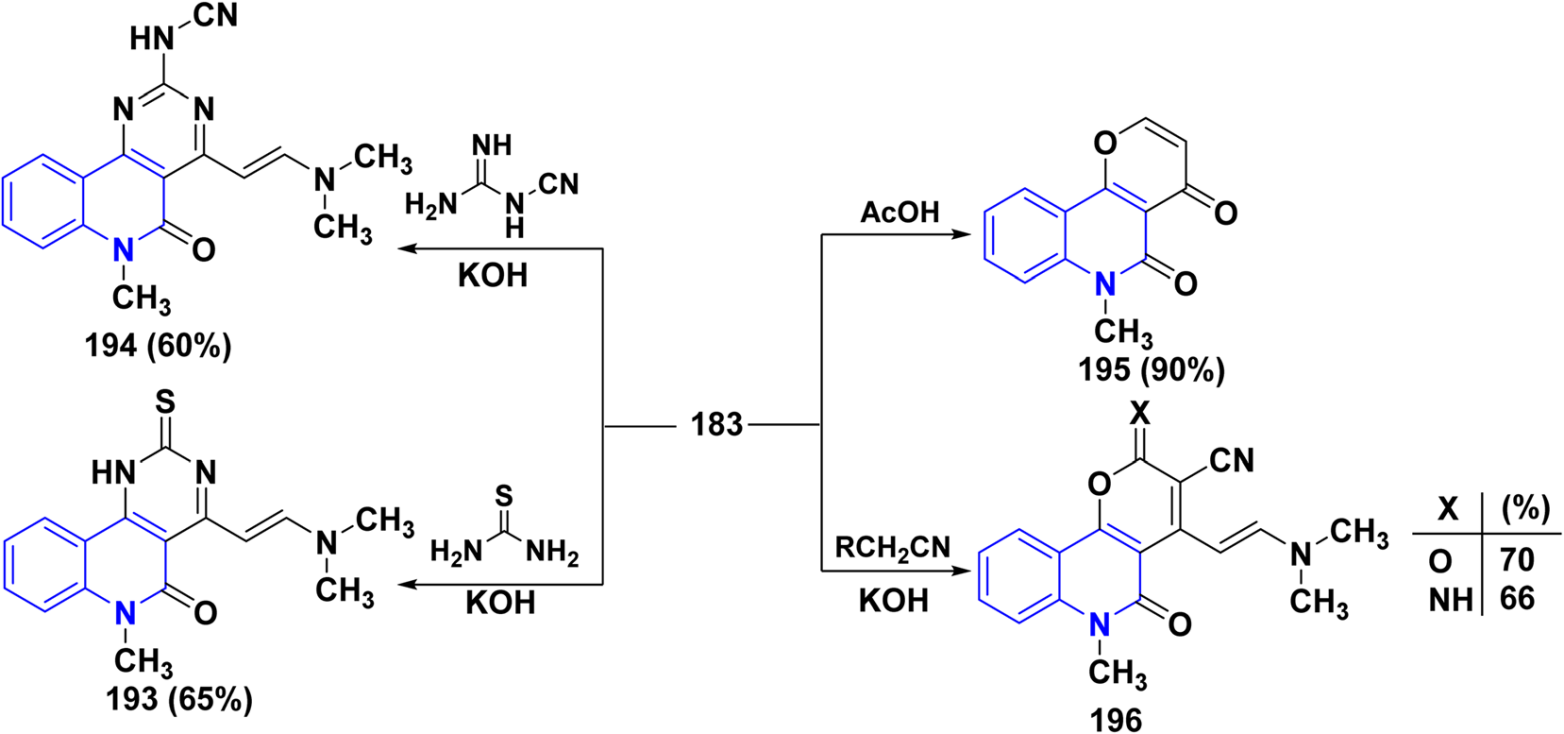
Reaction series of enaminone 183 analogues synthesis.

Additionally, treatment of the enaminone 183 with thiourea in ethanolic potassium hydroxide solution did not give the expected pyrimidinylquinolinone 193. Similarly, reaction of enaminone 183 with cyanoguanidine as amidine reagent gave another pyrimido[5,4-*c*]quinolinone derivative 194. Moreover, when enaminone 183 was heated in AcOH, it went through intramolecular cyclocondensation, resulting in the formation of 6-methylpyrano[3,2-*c*]quinolindione 195. Finally, when enaminone 183 was reacted with compounds containing active methylene like malononitrile and ethyl cyanoacetate in the presence of KOH, it yielded the pyranoquinolinone derivatives 196 (Scheme 70).^178^

**Scheme 71:**
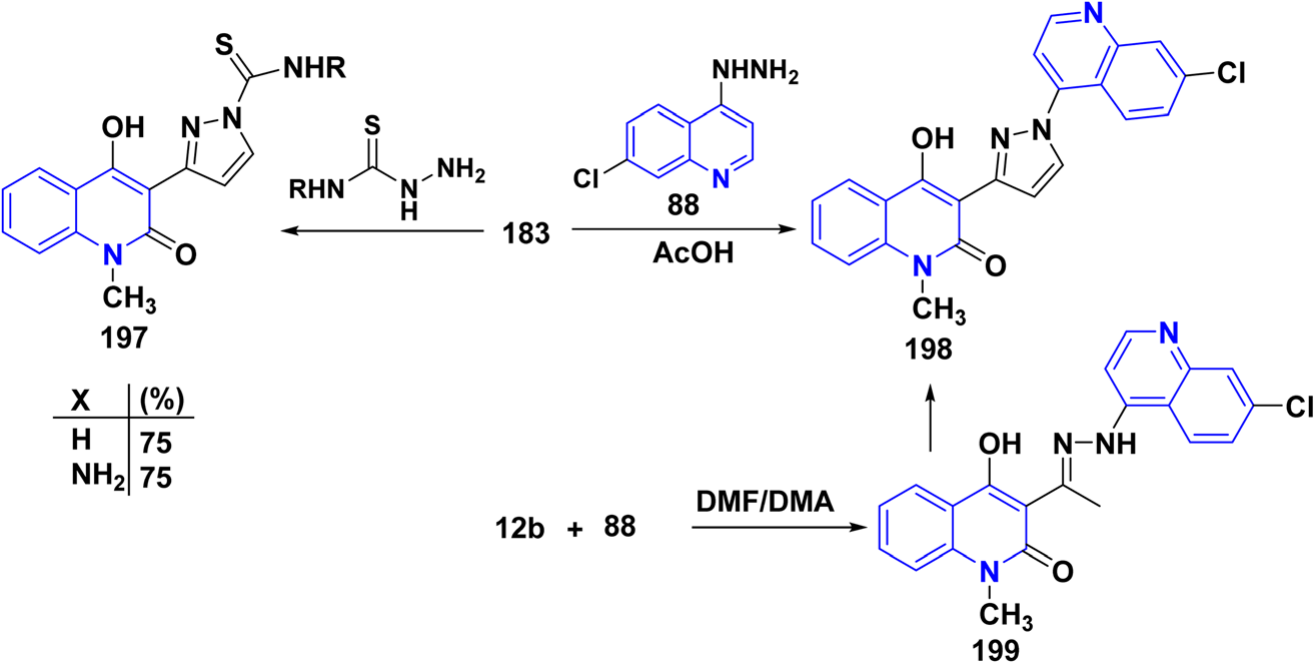
Reaction of enaminone 183 with binucleophilic reagents.

Moreover, treatment of the enaminone 183 with thiosemicarbazide and thiocarbodihydrazide yielded 197. The cyclocondensation of 7-chloro-4-hydrazinoquinoline 88 with enaminone 183 in acetic acid resulted in the formation of 4-hydroxy-1-methyl-3-[1-(7-chloroquinolin-4-yl)-1*H*-pyrazol-3-yl]quinolinone 198. The proposed structure for compound 198 was further validated through an alternative synthesis starting from acetylquinolinone 12b. Specifically, treating compound 12b with compound 88 yielded hydrazone 199. Subsequently, the *in situ* thermal condensation of hydrazone 199 with DMF/DMA smoothly produced compound 198 (Scheme 71).^178^

**Scheme 72:**
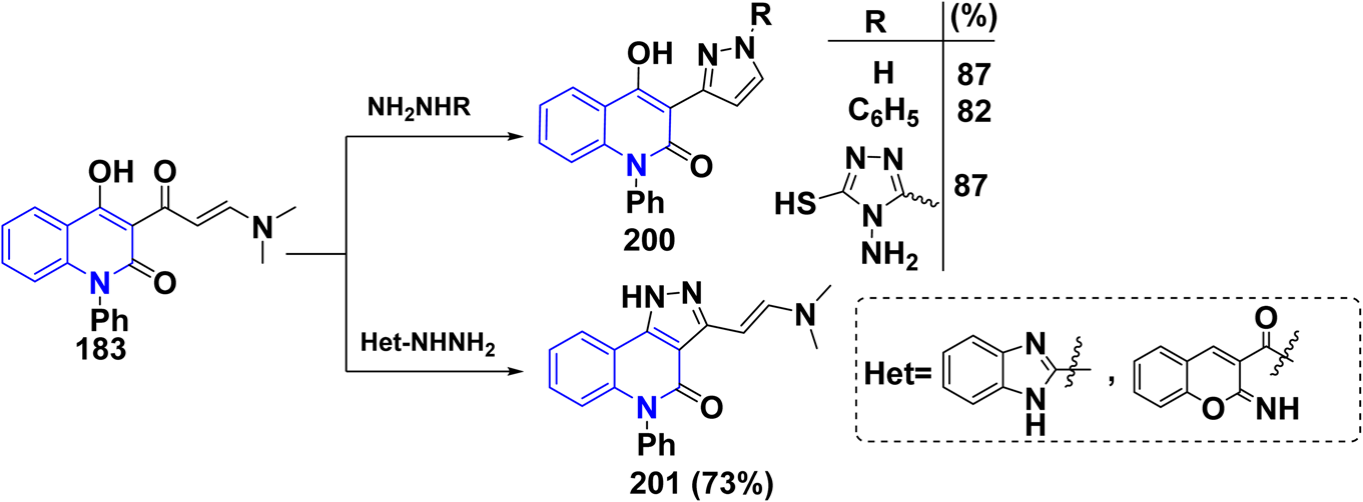
Reaction of enaminone 183 with different hydrazine derivatives.

Reaction of enaminone 183 with different hydrazines and hydrazides, such as hydrazine hydrate, phenylhydrazine, and 4-amino-5-hydrazineyl-4*H*-1,2,4-triazole-3-thiol afforded binary pyrazole 200 in good yield. Whereas, treatment of 183 with heterocyclic hydrazine as 2-hydrazineyl-1*H*-benzo[*d*]imidazole or 2-imino-2*H*-chromene-3-carbohydrazide yielded chemoselective fused pyrazole (*E*)-3-(2-(dimethylamino)-5-phenyl-4*H*-pyrazolo[4,3-*c*]quinolin-4-one (201) (Scheme 72).^192^

**Scheme 73:**
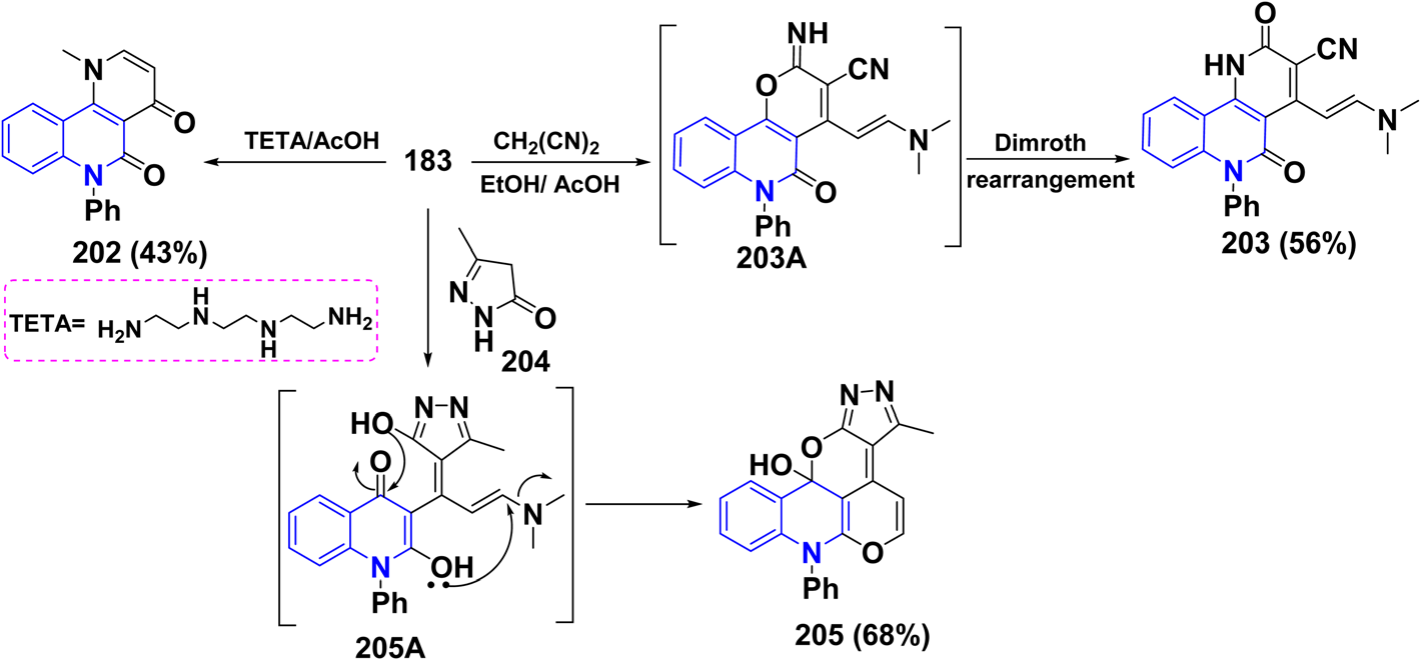
Synthesis of fused heterocyclic scaffolds 202, 203, and 205.

Reaction of enaminone 183 with triethylenetetramine (TETA) as highly active linear polyamines in CH_2_Cl_2_ led to the formation of benzo[*h*][1,6]naphthyridine-4,5-dione derivative 202 instead of the anticipated transamination products. Furthermore, refluxing of 183 with malononitrile led to the formation of tetrahydrobenzo-[*h*][1,6]naphthyridine-3-carbonitrile 203 through 1,2-addition mechanism followed by the nucleophilic attack of OH group to the cyano function. After that, the Dimroth rearrangement was achieved instead of an alternative path involving the replacement of the active methylene with the dimethylamino group (Scheme 73). Refluxing of enaminone 183 with pyrazolone 204 in AcOH gave fused triazaindenoanthracenol 205 (Scheme 73). The mechanistic pathway for the formation of 205 was recognized through a condensation reaction afforded 1,5-dicarbonyl intermediate 205A, followed by cyclization reactions of intermediate 205A through double nucleophilic attacks of the both two OH groups to the endo-ketonic carbonyl group of quinolinone and *β*-position of enaminone led to affording the fused polycyclic system 205 in 68% yield (Scheme 73).^192^

#### 4.3.3. With esters

**Scheme 74:**
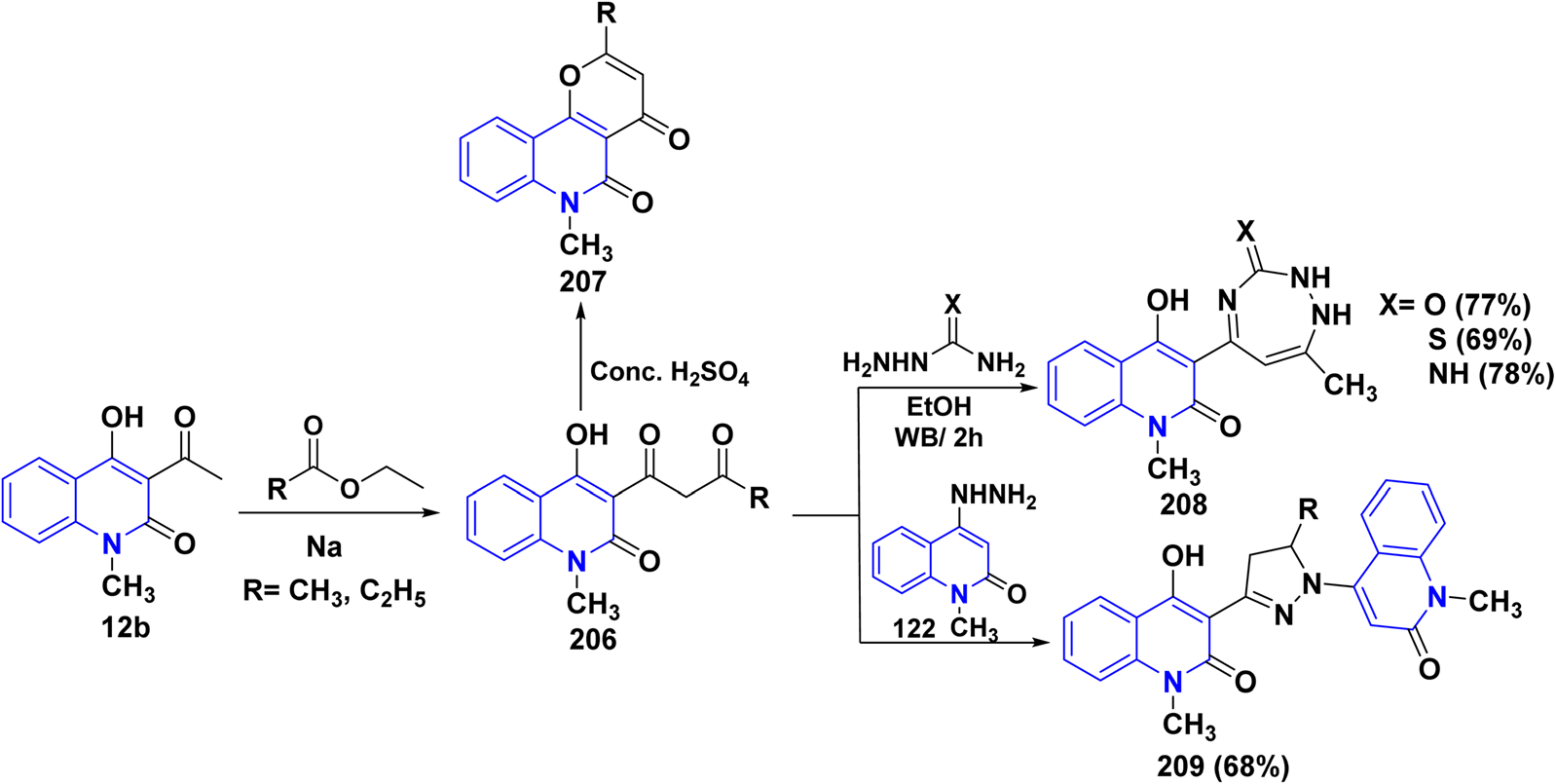
Synthesis and cyclization reactions of compound 206.

Compound 206 was synthesized through the condensation of quinolinone derivative 12b with methyl/ethyl propionate in the presence of Na metal. This reaction occurred through the nucleophilic attack of the active methylene group on the carbonyl group of the ester reagent. Cyclization of compound 206 with sulfuric acid gave 2-ethyl-6-methylpyrano[3,2-*c*]quinolindione 207 (Scheme 74).^151^ The bifunctional ammonia derivatives such as semicarbazide, thiosemicarbazide or aminoguanidine reacted with 206 in refluxing ethanol to furnish a binary triazepinone, triazepinthione and iminotriazepine 208, respectively. Furthermore, reaction of compound 206 with hydrazinoquinolinone 122 afforded the intended dihydromethylquinolinyl)-3-pyrazolyl)-4-hydroxyquinolinones 209 (Scheme 74).^176^

**Scheme 75:**
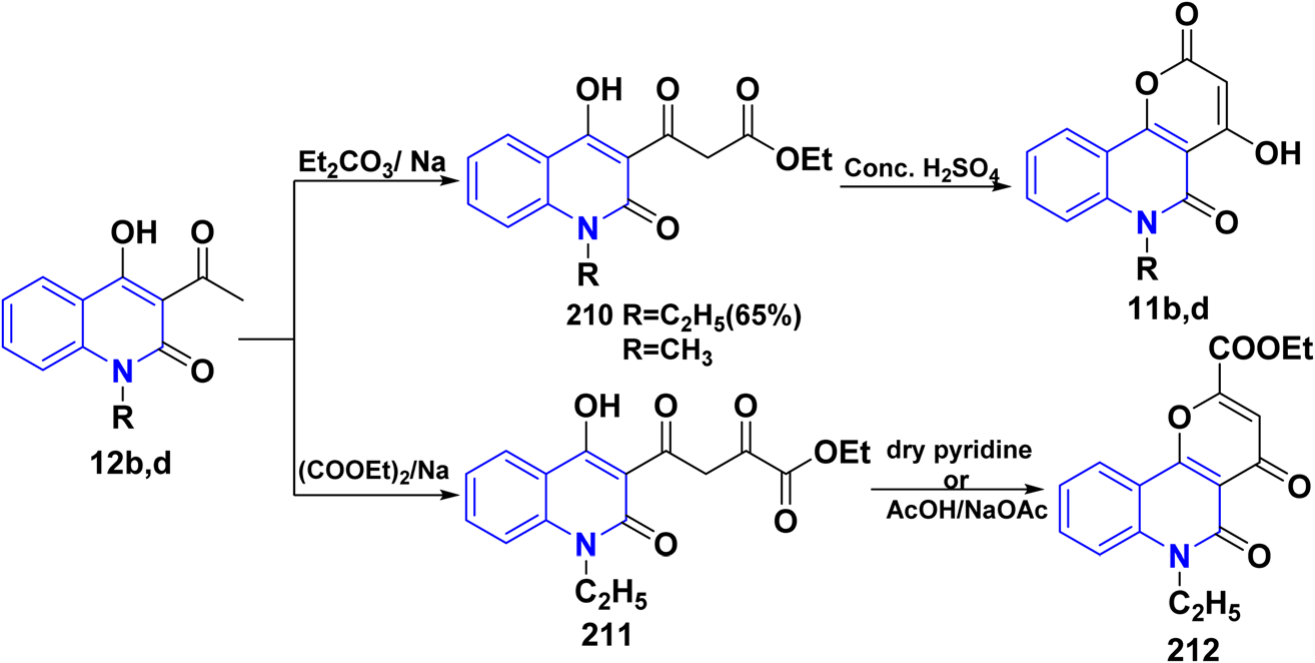
Claisen condensation of compound 12b,d with different esters.

Additionally, Claisen condensation reaction of 12b,d with diethyl carbonate led to 1,3-diketone; 3-ethoxycarbonylacetyl-4-hydroxyquinolin-2-one 210 in the presence of sodium metal. Cyclization of compound 210 with sulfuric acid afforded 4-hydroxypyrano[3,2-*c*]quinolindione 11b,d. In a similar process, Claisen condensation of compound 12d with diethyl oxalate in the presence of Na metal produced the diketo-ester; (ethylhydroxy-1,2-dihydroquinolinyl)dioxobutyrate 211. Cyclization of 211, in dry pyridine, or AcOH/AcONa (freshly fused) yielded ethyl 6-ethyldihydropyrano[3,2-*c*]quinoline-2-carboxylate 212 (Scheme 75).^151^

**Scheme 76:**
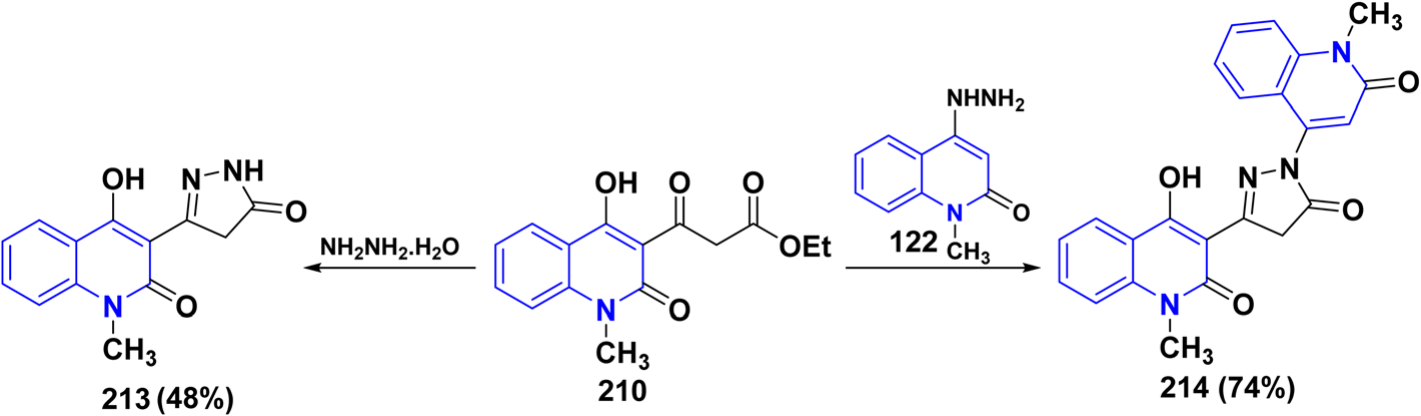
Synthesis of pyrazolonyl moiety anchored on quinolone 213 and 214.

The synthesis of binary pyrazolylquinolinone 213 was achieved by reacting the *β*-ketoester 210 with hydrazine hydrate to give the target compound 213 in 48% yield.^193*a*^ While, direct treatment of pyranoquinolindione 11 with hydrazine hydrate gave the desired pyrazolylquinoline 213 in 92% yield. The possibility of forming the isomeric quinolinylpyrazolin-3-yl)quinolinone 214 was ruled out by independently synthesizing this derivative through the cyclization of hydrazinoquinolinone 122 with 3-ethoxycarbonylacetyl-4-hydroxy-1-methylquinolinone 210 in 74% yield (Scheme 76).^176*b*^

**Scheme 77:**
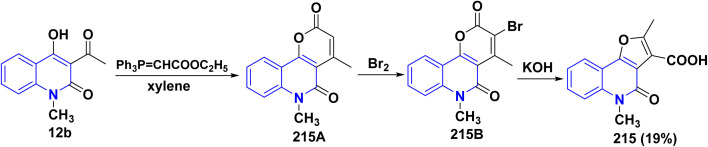
Synthesis of furo[3,2-*c*]quinolone 215.

Whereas, the reaction of acetylquinolinone 12b with ethyl (triphenylphosphoranylidene)acetate in refluxing xylene afforded 215A followed by bromination to give compound 215B that underwent hydrolysis gave furo[3,2-*c*]quinolinone 215 (Scheme 77).^193*b*^

**Scheme 78:**
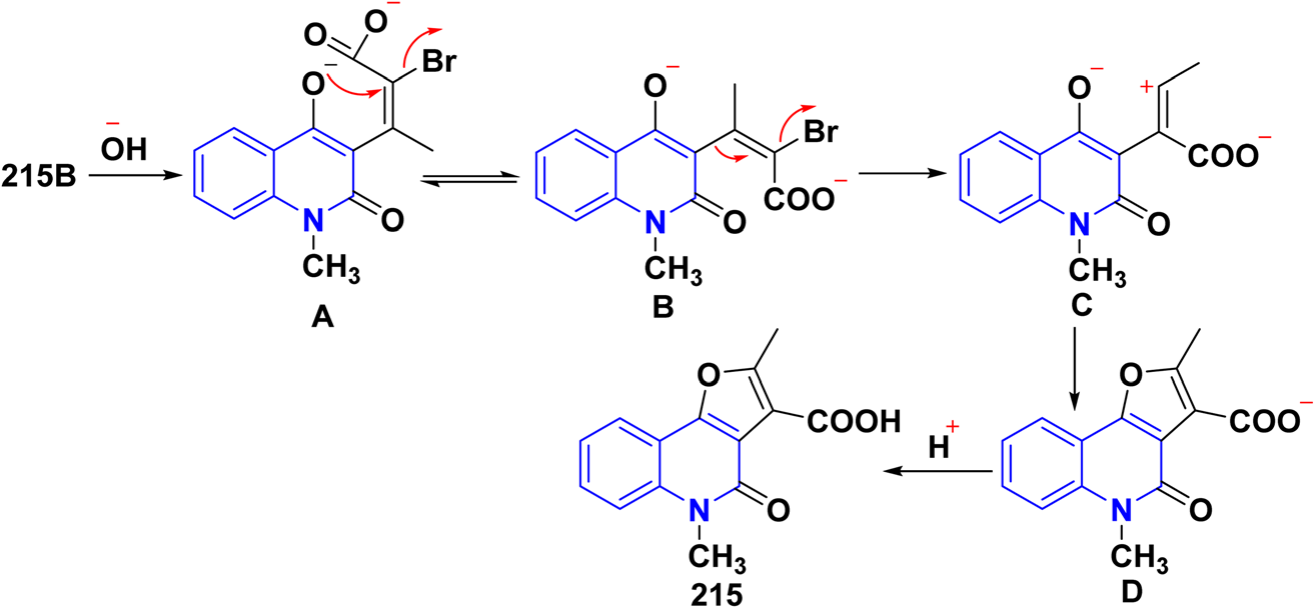
Plausible mechanism for the synthesis of 215.

The proposed reaction mechanism for the molecular rearrangement of 215B to acids 215 begins with the opening of the pyrane ring in a basic medium, resulting in the formation of dianion A. Tautomer B is considered more likely than A due to the greater distance between the two negatively charged functional groups. Following this, the elimination of a halogen atom from the sp^2^ hybridized carbon atom occurs alongside the migration of the substituent in a *trans* position relative to the departing halogen. This migrating group exhibits partial carbanionic character due to its potential tautomerism. This rearrangement, which can be classified as a Wagner–Meerwein type, leads to the formation of intermediate C, which subsequently yields compound D and ultimately product 215 after acidification (Scheme 78).

**Scheme 79:**
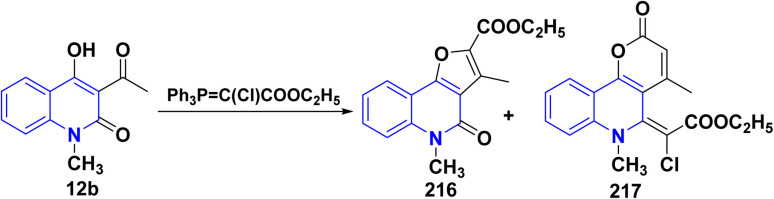
Reaction of quinolone 12b with ethyl (triphenylphosphoranylidene)chloroacetate.

Treatment of 12b with ethyl (triphenylphosphoranylidene)chloroacetate in refluxing xylene afforded a complex mixture of compounds 216 and 217. Compound 216 was isolated by repeated column chromatography and identified as an ethyl ester. The second reaction product 217 contained an unsaturated lactone moiety (Scheme 79).^193,194^

#### 4.3.4. With activated olefins as synthetic routes to pyrano skeletons and their benefits (ylidene nitriles)

**Scheme 80:**
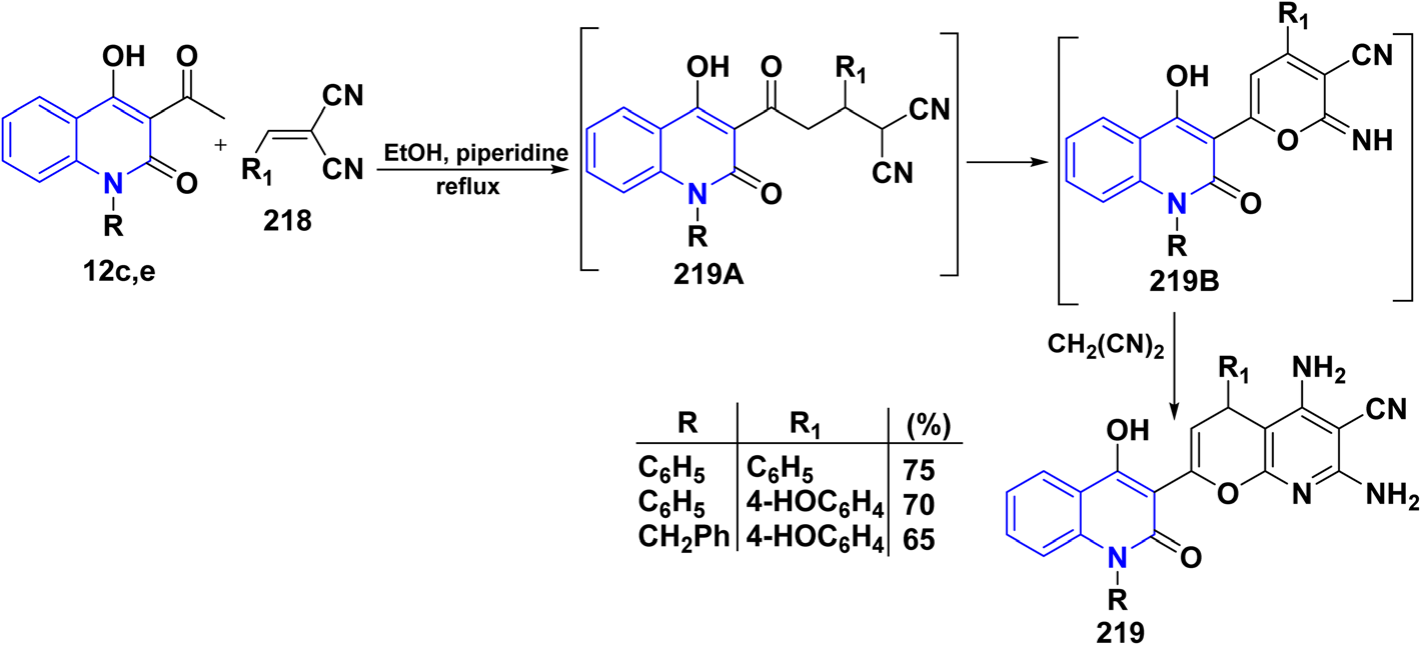
Michael addition of compounds 12c,e.

A Michael reaction refers to a type of organic chemistry reaction involving the addition of a nucleophile (Michael donor) to an activated olefin or alkyne (Michael acceptor), such as an acrylate. This reaction is known for its rapid occurrence, tolerance of various functional groups, and its utility in synthesizing novel step-growth compounds with tailored macromolecular architectures. Derivatives 219 are similarly synthesized *via* Michael type addition of the anion ion of the acetyl group in 12c,e to the activated double bond in ylidenenitriles 218 in (1 : 1) or (1 : 2) molar ratios in ethanol/piperidine to yield the acyclic intermediate 219A, which dehydrogenated and undergo cyclization into the adducts 219B which afford compounds 219 under basic conditions by adding one molecule of CH_2_(CN)_2_, which exists in equilibrium with two moles (Scheme 80).^191^

**Scheme 81:**
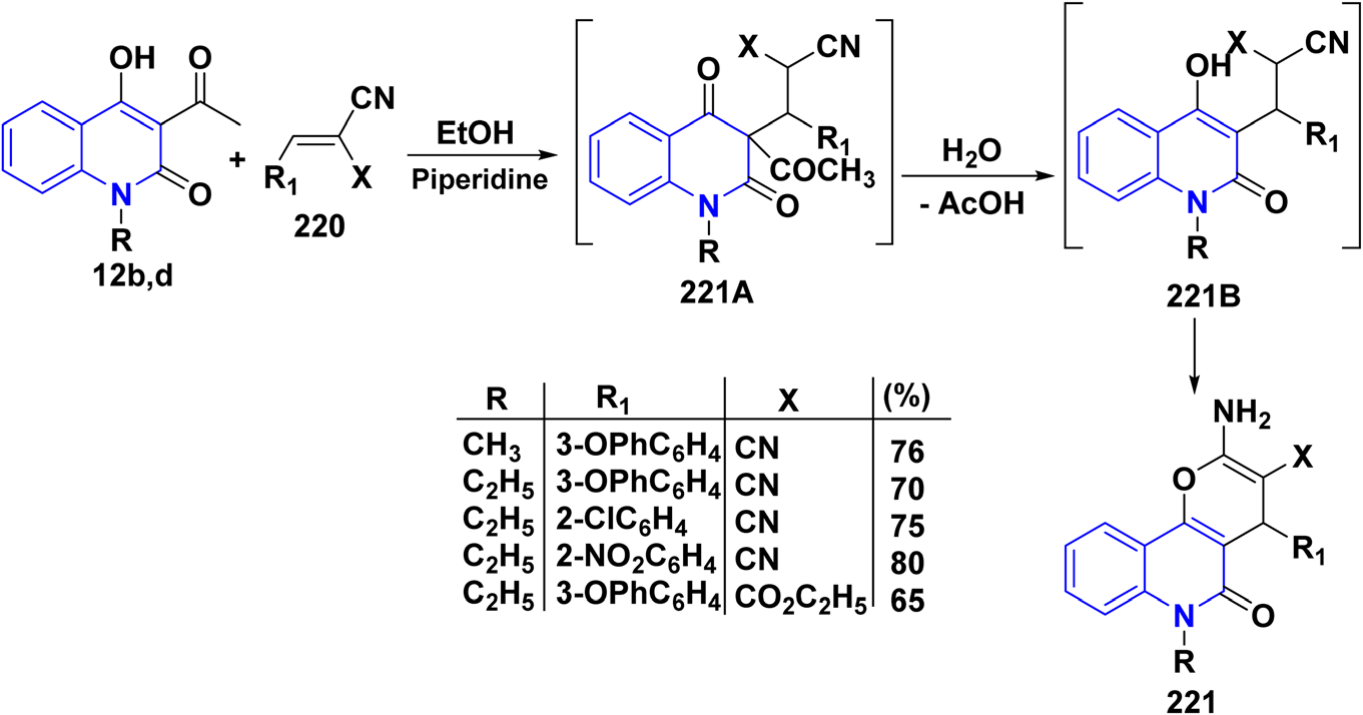
Catalyzed nucleophilic addition of 3-acetylquinolinones 12b,d.

On the other hand, the nucleophilic addition of 3-acetylquinolinones 12b,d to 220 in EtOH, using piperidine as a catalyst, produced intermediate 221A. This intermediate underwent hydrolysis, losing the acetyl group, to form quinolone intermediate 221. Subsequently, cyclization of this intermediate yielded 2-amino-5-oxopyrano[3,2-*c*]quinoline derivatives 221 (Scheme 81).^115,134,195^

**Scheme 82:**
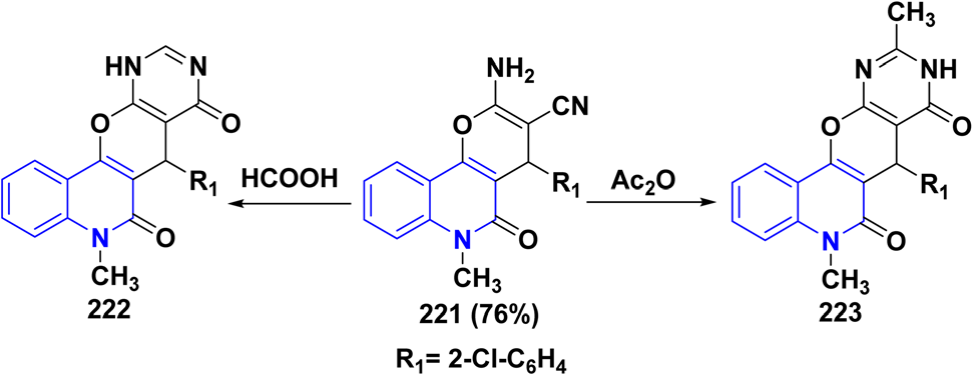
Synthetic pathway to access tetracyclic systems 222 and 223.

2-Amino-4-(2-chlorophenyl)oxopyrano[3,2-*c*]quinolinecarbonitrile 221, as a typical enaminonitrile derivative, reacted with HCOOH to afford tetracyclic 5-methylpyrimidopyrano[3,2-*c*]quinolindione 222. Compound 221 also reacted with Ac_2_O to yield 5,10-dimethylpyrimidopyrano[3,2-*c*]quinolindione 223 (Scheme 82).^195^

**Scheme 83:**
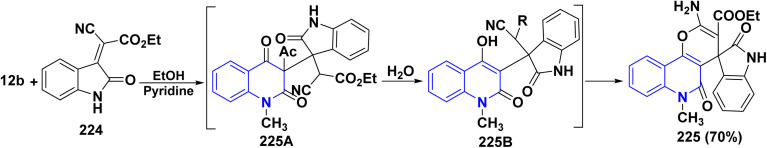
Synthetic approach of spiro compound 225.

6-Methyldioxodihydrospiro[indoline-3,4′-pyrano[3,2-*c*]-quinoline]-3′-carboxylate 225 was synthesized through the reaction of compound 12b with 224 in EtOH, pyridine (Scheme 83).^115^

### 4.4. Miscellaneous reactions

#### 4.4.1. Deacetylated quinolinone (HQ) and its utility

**Scheme 84:**
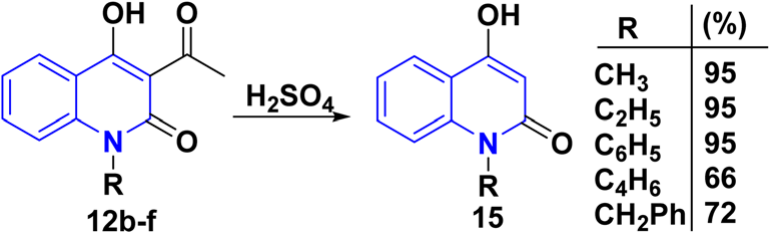
Deacetylation of 3-acetyl-4-hydroxyquinolone derivatives 12b–f.

Hydrolysis reaction of acetylquinolinone 12b–f with sulfuric acid, accompanied by deacetylation produced 4-hydroxyquinoline derivatives (HQ) 15 (Scheme 84).^115,130,132,141,145,155^

**Scheme 85:**
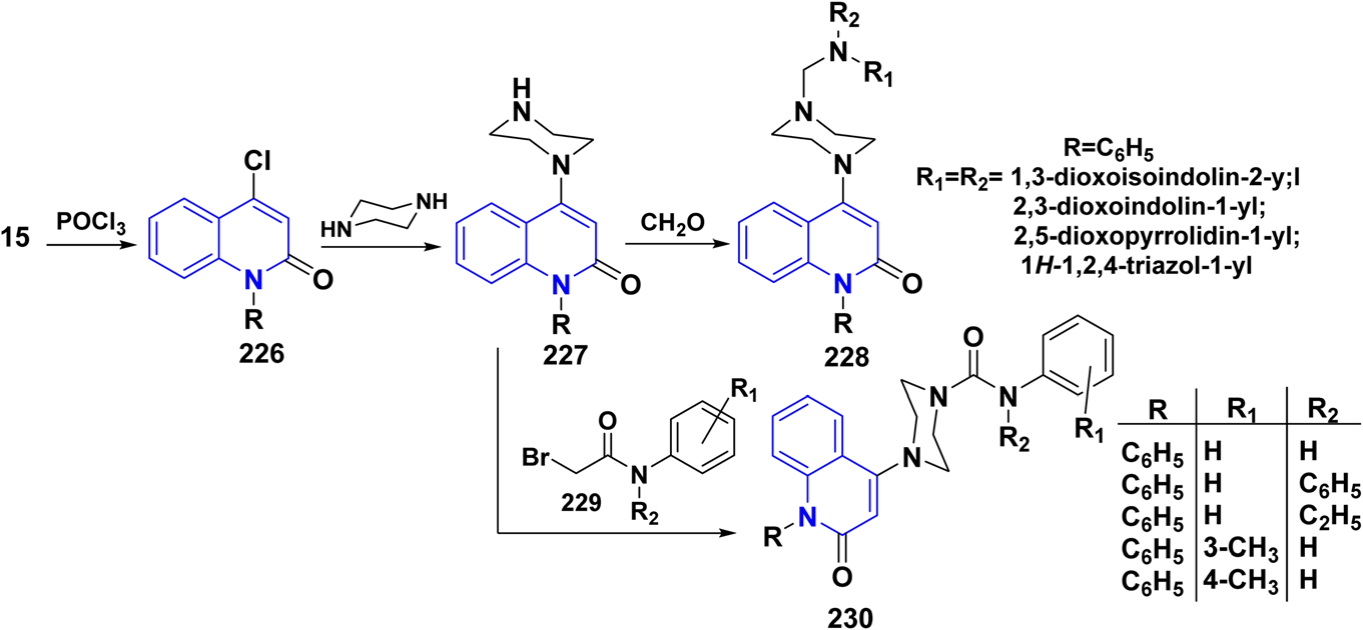
Chloronation of 15 followed by synthesis of *N*-Mannich base.

Refluxing of 4-hydroxyphenylquinolinone 15 with POCl_3_ for 2 h yielded the corresponding 4-chloroquinolinone derivative 226, reaction of 226 with three equivalent piperazine in DMF in the presence of TEA was heated at 80 °C for 6 h yielded 4-piperazinylquinolinone 227, heating 227 with formaldehyde and the appropriate amine in EtOH at reflux then leave overnight afforded the target *N*-Mannich bases 228. Alkylation of the piperazinyl nitrogen of compounds 227 with 2-bromo-*N*-phenylacetamide derivatives 229 was achieved by stirring overnight in DMF using K_2_CO_3_ as a base to yield the target compounds 230 (Scheme 85).^194^

**Scheme 86:**
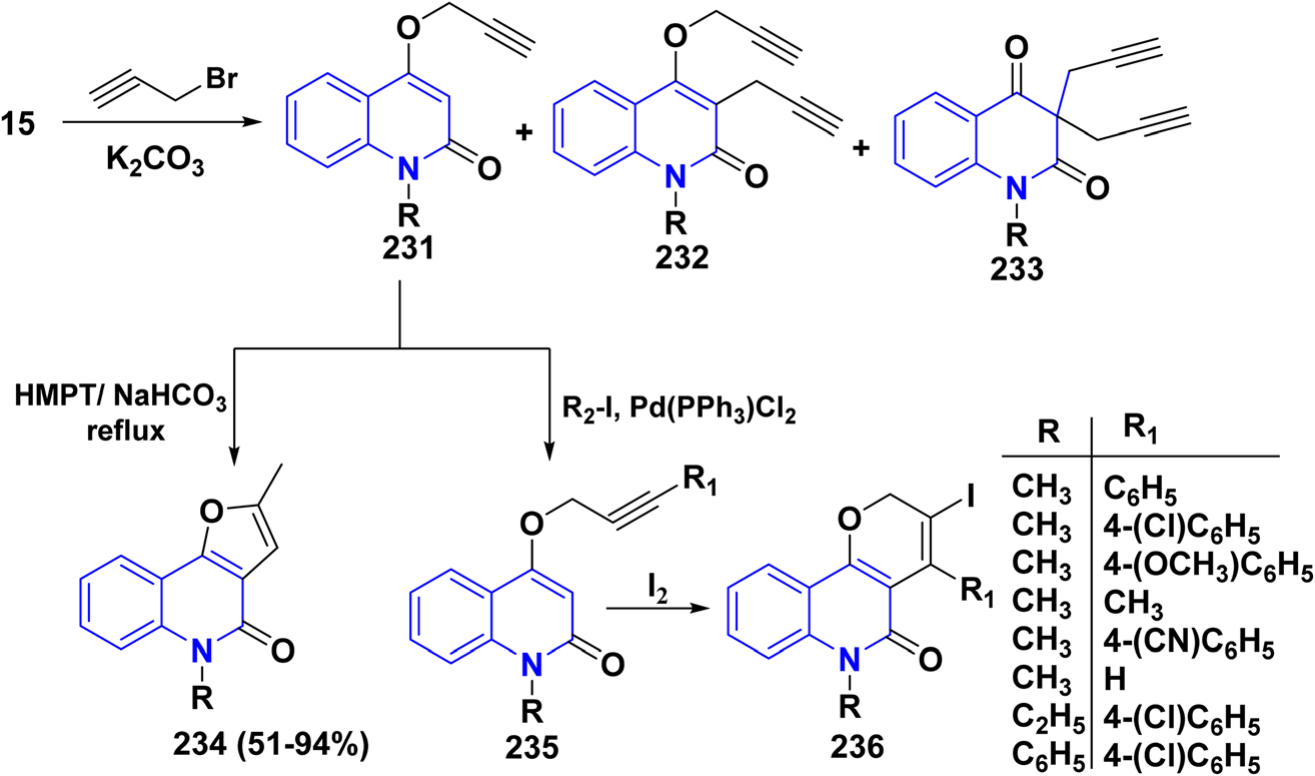
*O*-alkylation followed by Suzuki coupling reaction.

**Scheme 87:**
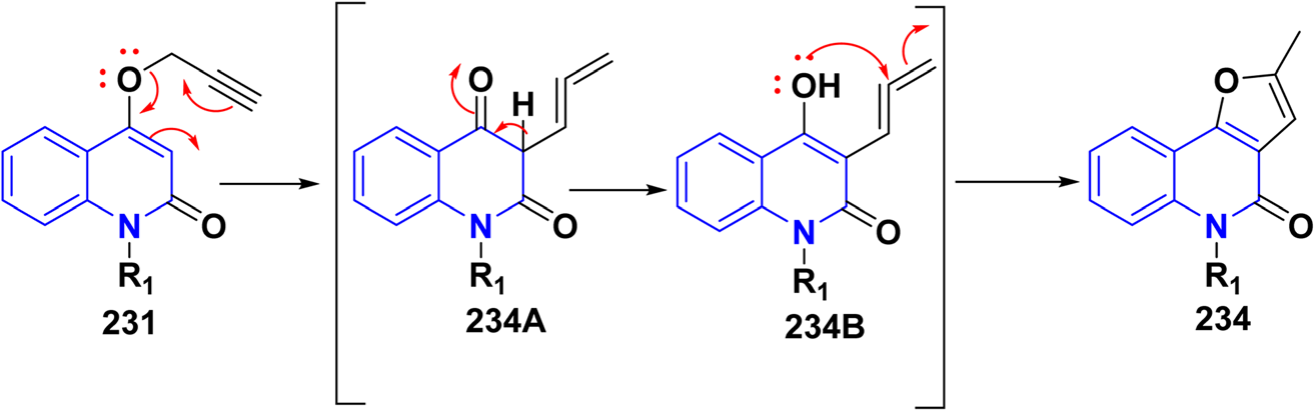
Mechanistic pathway for the synthesis of 234.

Refluxing of 15 with propargyl bromide in acetone in the presence of anhydrous K_2_CO_3_ for 14–16 h afforded a mixture of 231, 232, and 233, which were separated by HPLC.^196^ Base-catalysed Claisen rearrangement of propynyloxyquinolone 231 in hexamethylphosphoric triamide (HMPT) in the presence of NaHCO_3_ (2 equiv), refluxed for 10–18 h afforded angular furo[3,2-*c*]quinolinone 234 according to the shown mechanism (Schemes 86 and 87). Whereas, 231 products followed by Sonogashira coupling yielded 4-((3-arylprop2-yn-1-yl)oxy)quinolin-2-onederivatives 235 in good yield. Then, electrophilic cyclization between 235 and iodine to obtain 236, the reaction was initially performed in various solvents (DMF, CH_3_CN, CH_3_OH, THF, DCM, and CH_3_NO_2_) at 25 °C, using sodium bicarbonate as a base (Scheme 86).^197^

**Scheme 88:**
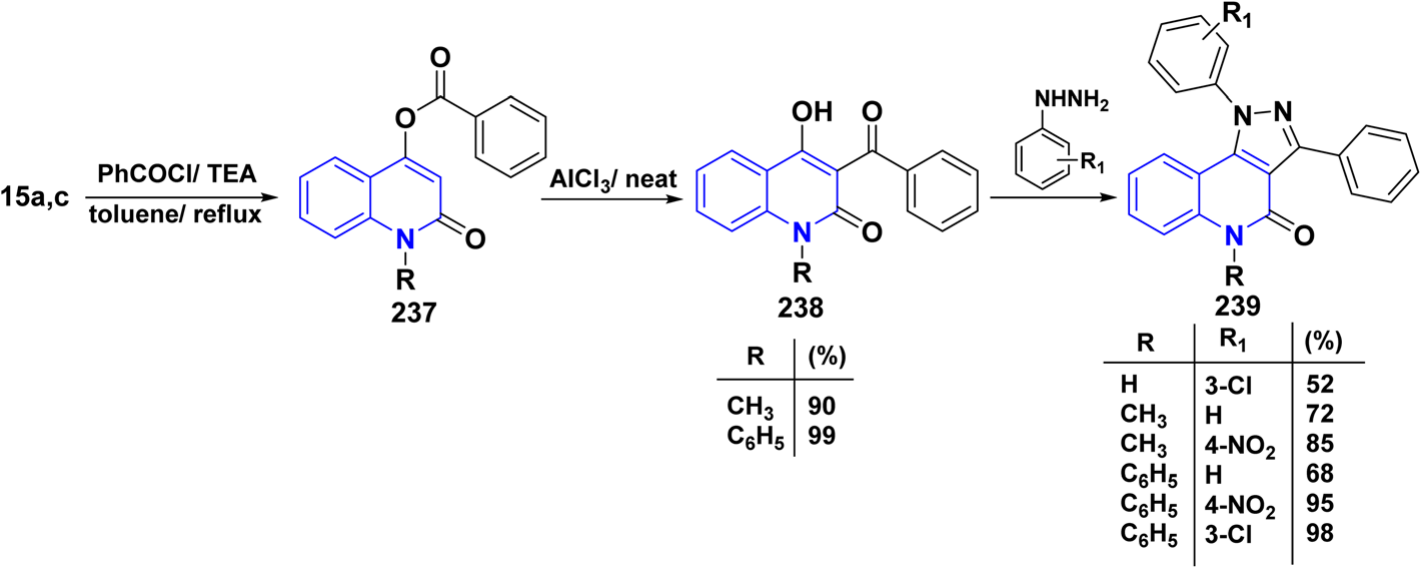
Synthesis of pyrazolo[4,3-*c*]quinolinones 239 through *o*-benzoylation.

Benzoylation reaction of 15a–c with benzoyl chloride and a catalytic amount of TEA afforded 4-benzoyloxy-2-quinolinones 237.^198^ Compound 237 underwent Fries rearrangement with AlCl_3_, yielding 3-benzoyl-4-hydroxy-2-quinolinones 238 in excellent yields. Reaction of *β*-hydroxyketone compound 238 with phenylhydrazines in dimethylformamide (DMF) at room temperature and also at 0 °C yielded mixtures of compounds that can be separated, which contained cyclized pyrazolo[4,3-*c*]quinolinones 239. To obtain pyrazolo[4,3-*c*]quinolinones 239 directly from 3-benzoyl-4-hydroxy-2-quinolinones 240 pure and in good yield (87–99%). A suspension of 3-benzoyl compounds 238 was treated in AcOH with a few drops of conc. H_2_SO_4_ to enhance the reaction rate and obtain good to excellent yields. Heating for 2 h yielded pyrazolo[4,3-*c*]quinolones 239 (Scheme 88).^135^

**Scheme 89:**
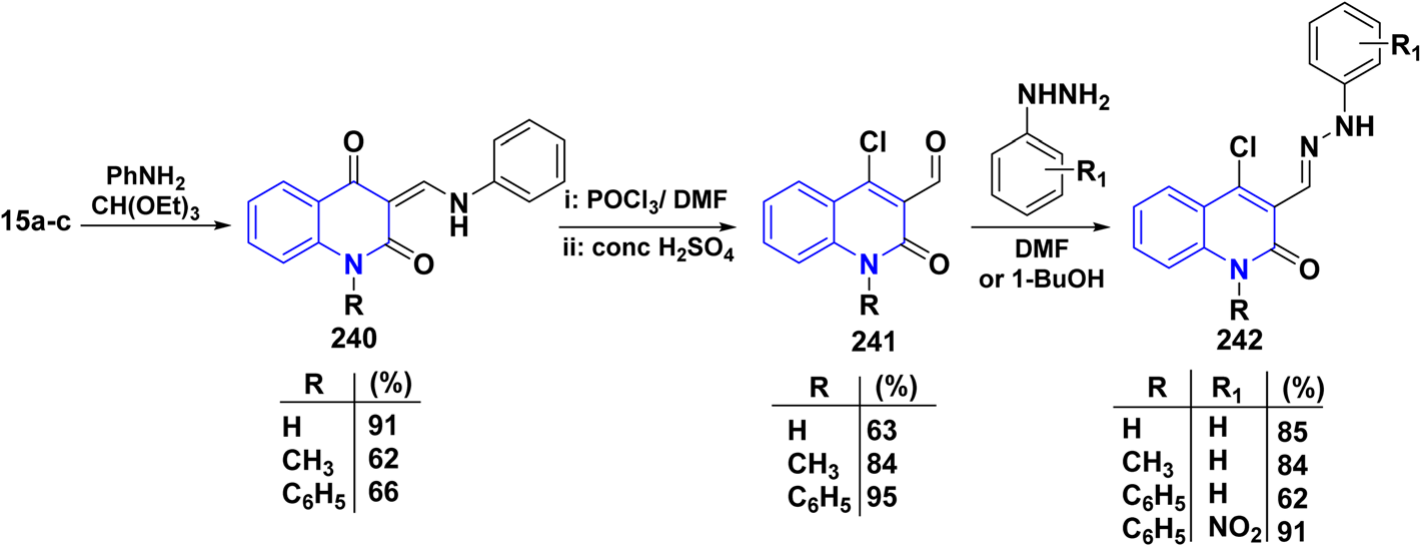
Synthesis of chlorohydrazonoquinolinones 242.

The synthetic pathway for compound 242 begins from 3-phenylaminomethylene quinolindiones 240, which were synthesized from 4-hydroxy 2-quinolones, aniline and triethyl orthoformate (CH(OEt)_3_), after that converted to 4-chloro-3-formylquinolinones 241.^135,199^ The reaction of carboxaldehydes 241 with a specific amount of phenylhydrazines in a solution of DMF at room temperature or in refluxing BuOH afforded 4-chloro-3-arylhydrazinonoquinolinones 242 in good to excellent yields (Scheme 89).^135^

**Scheme 90:**
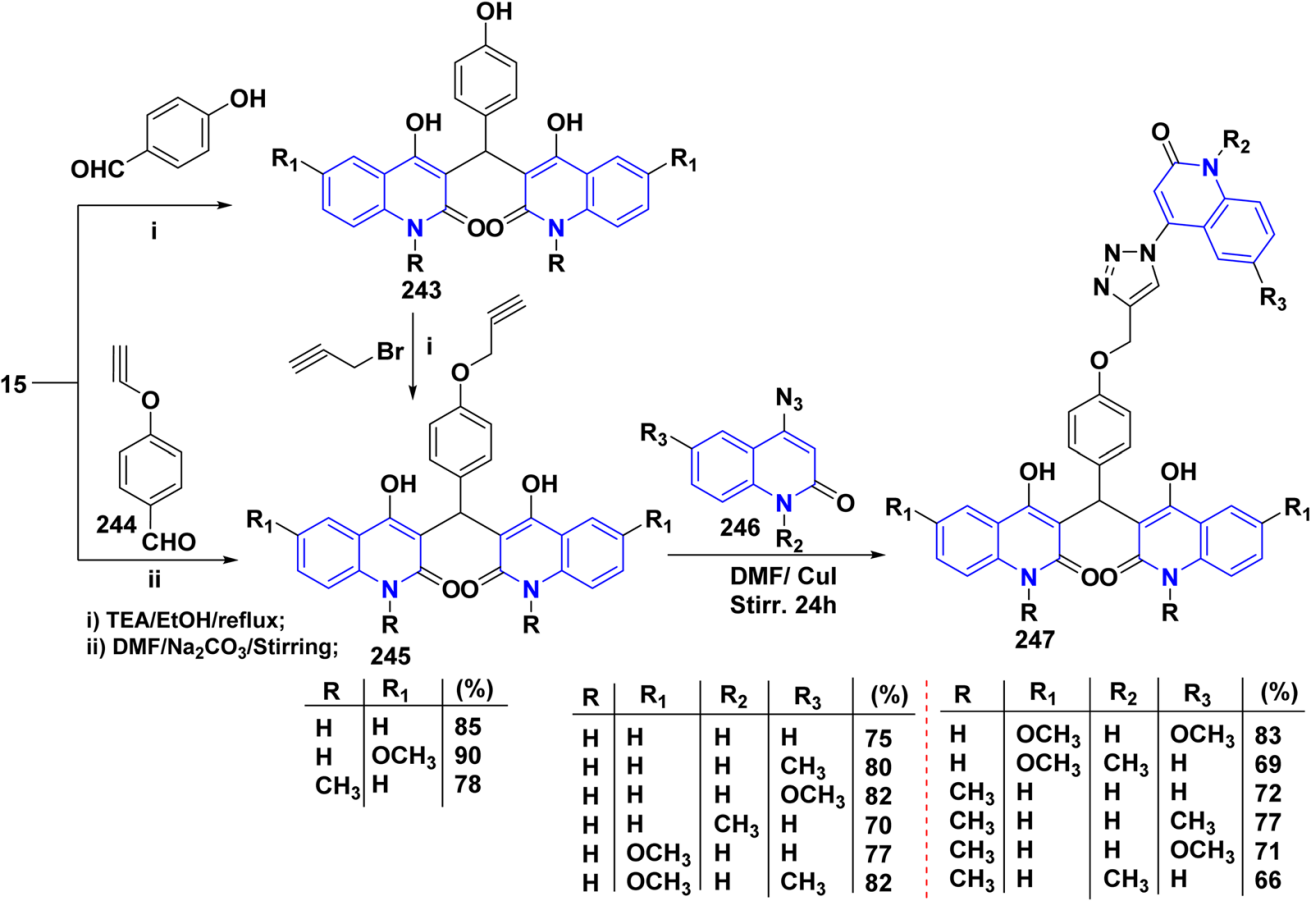
Synthesis of triazolyl-*bis*(4-hydroxy-quinolin-2(1*H*)-ones) 247.

The synthesis of a new series of triazolylquinolinones, specifically 3,3′–(oxoquinolin-4-yl)-1,2,3-triazol-4-yl)methoxy)phenyl)methylene)bis(4-hydroxyquinolinones) 247, was achieved by reacting 4-azido-2-quinolinones 246 with 3,3′–(prop-2-yn-1-yloxy)phenyl)methylene)bis(4-hydroxyquinolinones) 245 through a Cu-catalyzed [3 + 2] cycloaddition (Huisgen–Meldal–Sharpless reaction). Initially, the synthesis of terminal alkynes 245 was performed *via* interaction between 4-hydroxy-quinoline-2-ones 15 and *p*-hydroxybenzaldehyde in different molar ratios under reflux to afford 3,3′–(hydroxy-phenyl)methylene)*bis*(4-hydroxyquinolin-2-ones) 243. Phenol compound 243 reacts with propargyl bromide in DMF to produce alkynes 245. On the other hand, 4-hydroxy-2-quinolinones 15, interacted with aldehyde 244 in different molar ratios (2 : 1) to produce the desired terminal alkynes 245 (Scheme 90).^200^

**Scheme 91:**
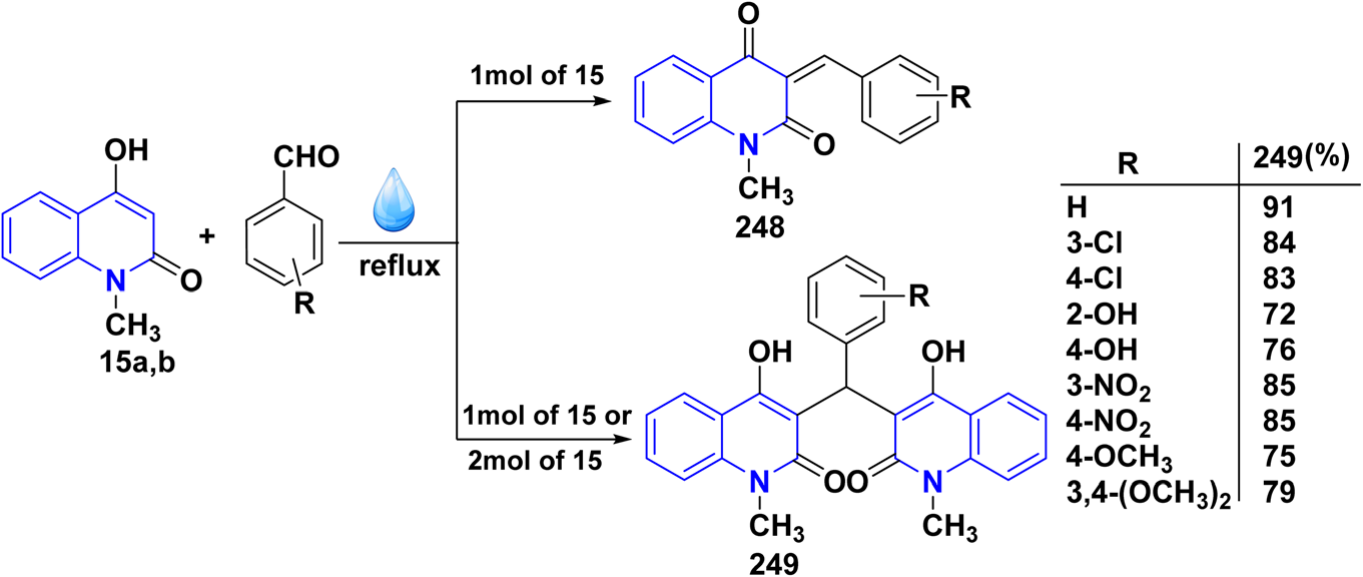
Reaction of 4-hydroxyquinoline derivatives 15 with various aromatic aldehydes.

Reaction of compound 15 with several aromatic and heterocyclic aldehydes, which contain both electron-withdrawing and electron-donating groups, was investigated under catalyst-free conditions using water as the eco-medium to produce compound 249, through Knoevenagel condensation followed by a Michael-type addition with compound 15, leading to the smooth formation of a wide range of substituted bisquinolinones. In these cases, aldehydes with electron-withdrawing groups produced higher product yields, ranging from 85% to 90%. The study revealed that compound 249 was the primary product, rather than compound 248, when using different moles of compound 15 (Scheme 91).^201–204^

**Scheme 92:**
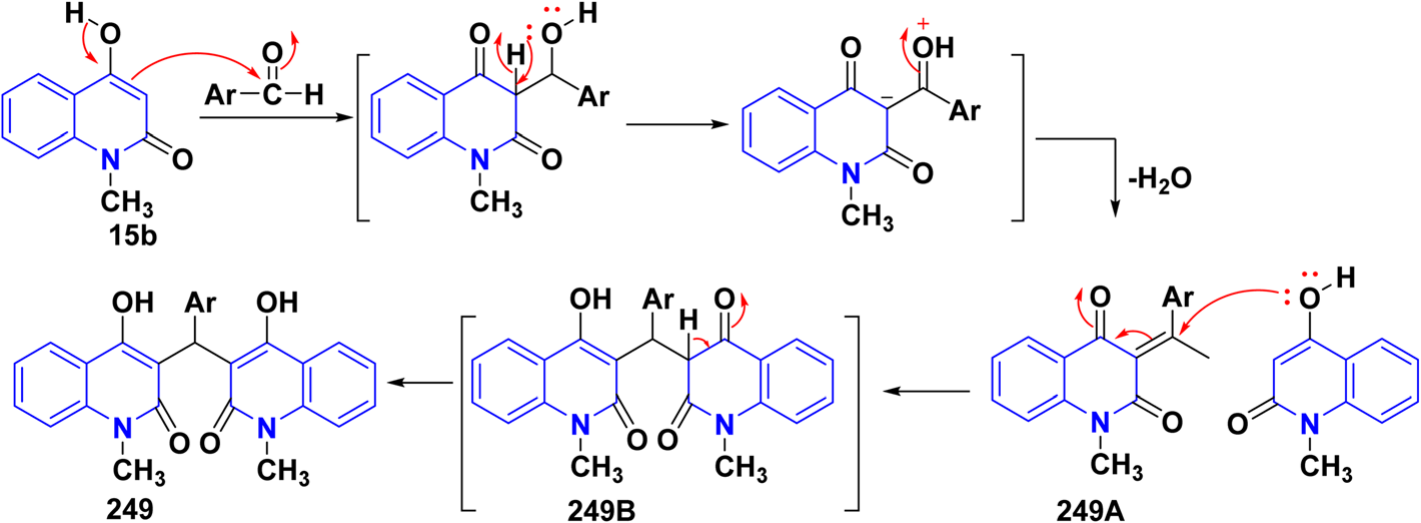
Mechanistic pathway for the synthesis of 249.

A believable mechanism for the formation of compound 15 has been suggested. Initially, one equivalent of HQ 15b undergoes a straightforward Knoevenagel condensation with aromatic and heterocyclic aldehydes to produce carbonyl intermediate 249A. This intermediate serves as a strong Michael acceptor and reacts with another equivalent of 15b through Michael addition, resulting in the keto–enol intermediate 249B, followed by isomerization to yield the final product (Scheme 92).^201^

**Scheme 93:**
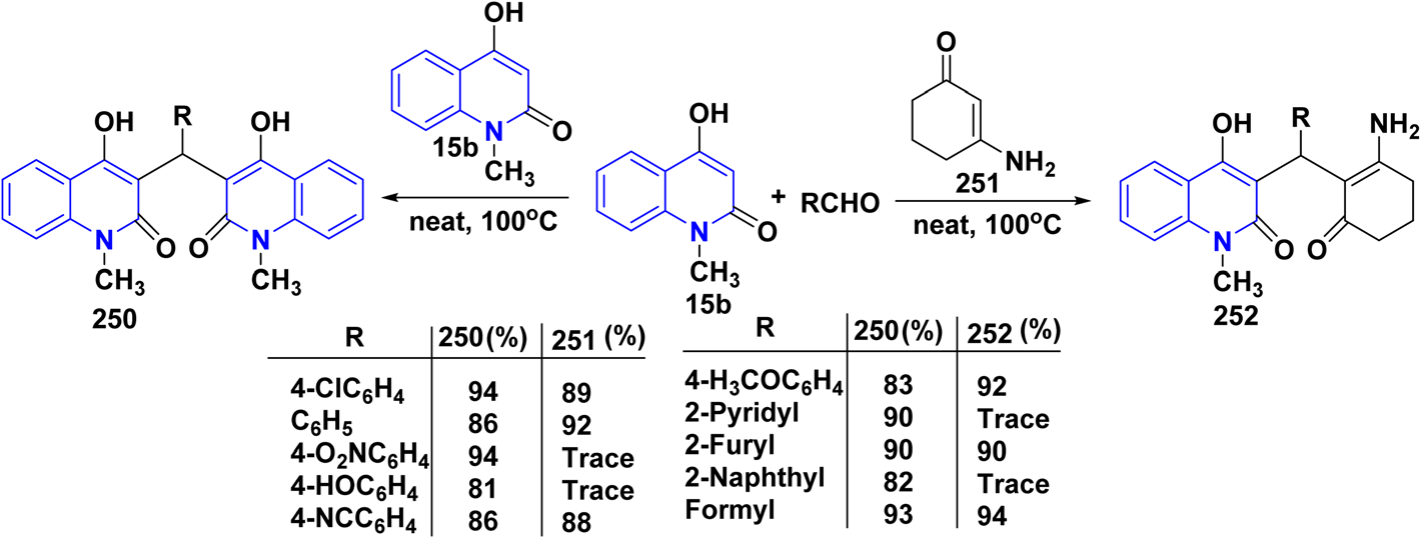
Synthesis of 250 and 252*via* Knoevenagel–Michael reaction.

Furthermore, treatment of 15b with the aromatic and heterocyclic aldehydes in different molar ratios (2 : 1) led to the formation of 250. Whereas, the three-component Knoevenagel–Michael reaction of 15b, aldehyde, and aminocyclohex-2-enone 251 produced skeleton 252. But, when the reactants were added sequentially, only a small amount of the desired product 252 was obtained, with compound 250 being the major product (Scheme 93).^203^

**Scheme 94:**
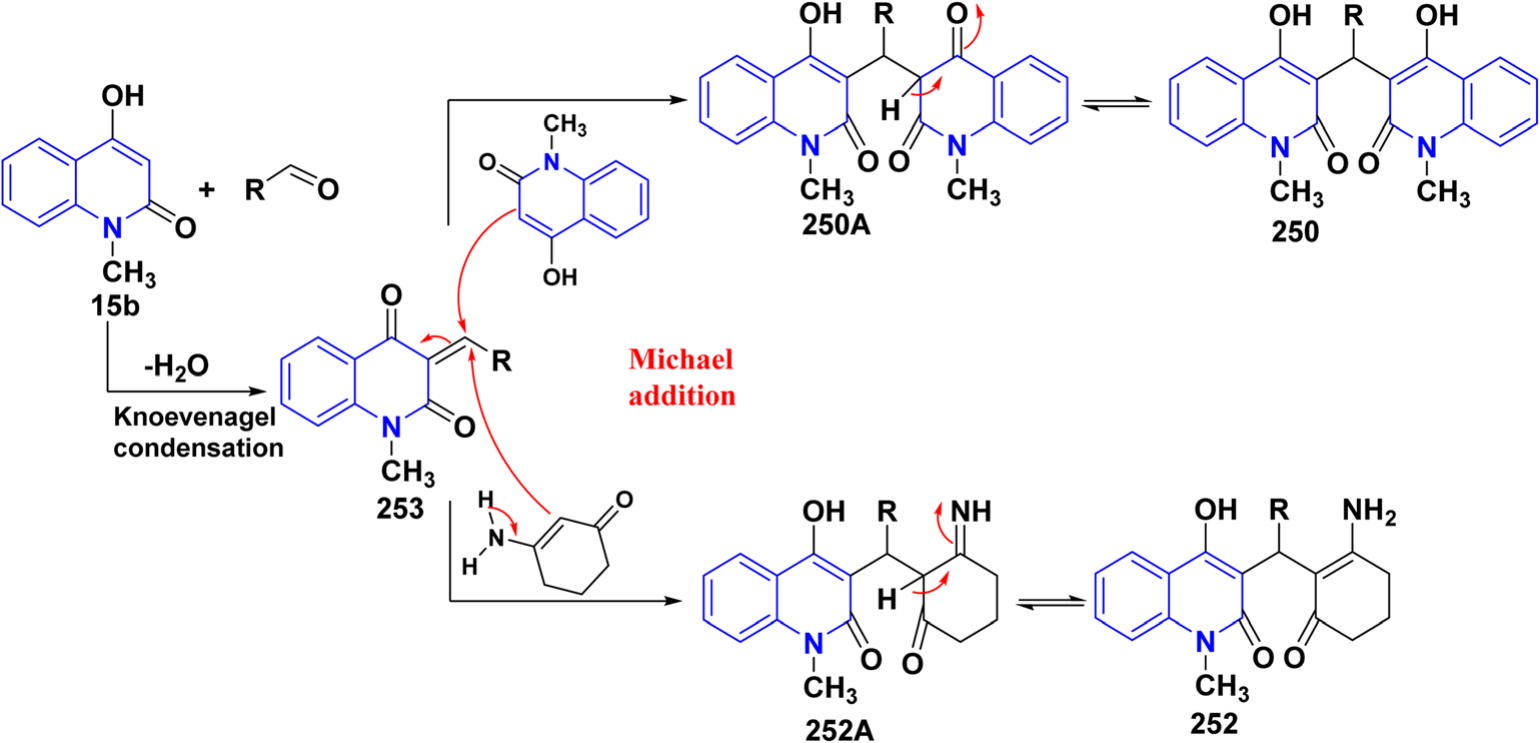
Suggested mechanism for the synthesis of 250 and 252.

A plausible mechanism for the formation of compounds 250 and 252 started with simple Knoevenagel condensation of 15 with the aromatic or heterocyclic aldehydes (known to occur under solvent and catalyst-free conditions) to generate an adduct 253, which acts as a strong Michael acceptor. After that, another molecule of HQ 15 (two-component reaction) or 3-aminocyclohex-2-enone (three-component reaction) attacks the electron-deficient *β* position of 253 in a Michael addition fashion to afford 250 and 252 (Scheme 94).^203^

**Scheme 95:**
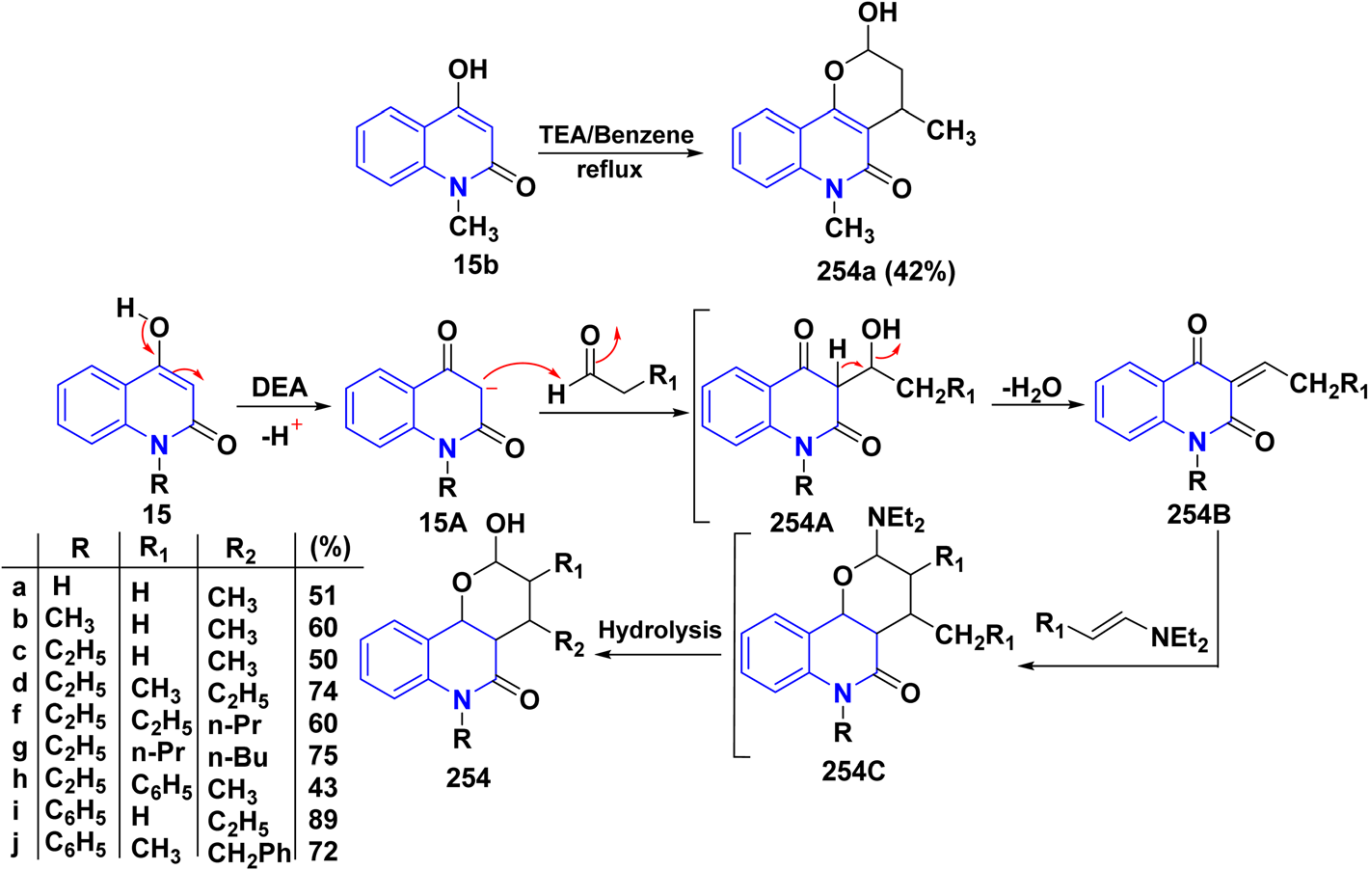
Photoinduced reaction of compound 15b with TEA producing 254a and the Plausible mechanism.

Pyrano[3,2-*c*]quinolinone derivatives represent a significant category of naturally occurring alkaloids. As their synthetic counterparts are currently the focuses of research due to their diverse biological activities with potential medicinal applications. Additionally, these compounds are frequently employed as synthetic precursors in producing dimeric quinoline and polycyclic heterocycles.^205^ While by, 2-hydroxydimethylpyrano[3,2-*c*]quinolin-5-one (254a) was synthesized by a photoinduced reaction of 15b in the presence of TEA, which generated DEA and acetaldehyde derivatives *in situ*, whereas, photoinduced electron–transfer reactions with TEA as an monodentate electron donating through a redox reactions were achieved according sketeched mechanism in Scheme 93.^206^ Reaction of 4-hydroxyquinolinone 15 with aliphatic aldehyde gave the corresponding quinolone methide, base-catalyzed condensation of a quinolinone 15 with an aldehyde yields the corresponding 4-hydroxy-3-(1-hydroxyethyl)quinolin-2-one 254A which underwent dehydration in basic medium to furnish the highly electrophilic quinone methide intermediate 254B. After that, Michael's addition reaction at the exocyclic methylene carbon of quinone methide 254B by the generated enamine (from DEA and the aldehyde) and the carbanion 15A derived from the deprotonation of 15. The Michael-type addition of the enamine proceeds in a 1,4-fashion and results in an intramolecular cyclization to yield 2-(diethylamino)pyrano[3,2-*c*]quinolin-5-one 254C which hydrolyzed to afford 254 (Scheme 95).^206^

#### 4.4.2. Oxidation

**Scheme 96:**
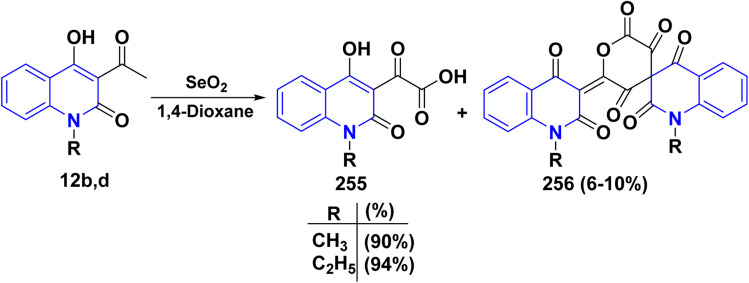
Oxidation of quinolone derivatives 12b,d.

Oxidation of quinolone derivatives 12b,d under the classic Riley conditions afforded a mixture of two products: *α*-keto acid 255 and its dehydrated dimer derivatives 256. The major product is soluble in aq. Na_2_CO_3_; besides, it can be isolated after neutralization to yield the *α*-keto acid 255, whereby the minor product is insoluble in aq. Na_2_CO_3_ solution, but soluble in boiling NaOH and crystallizable from AcOH and identified as spiropolyketonic scaffold 256 (Scheme 96).^207^

**Scheme 97:**
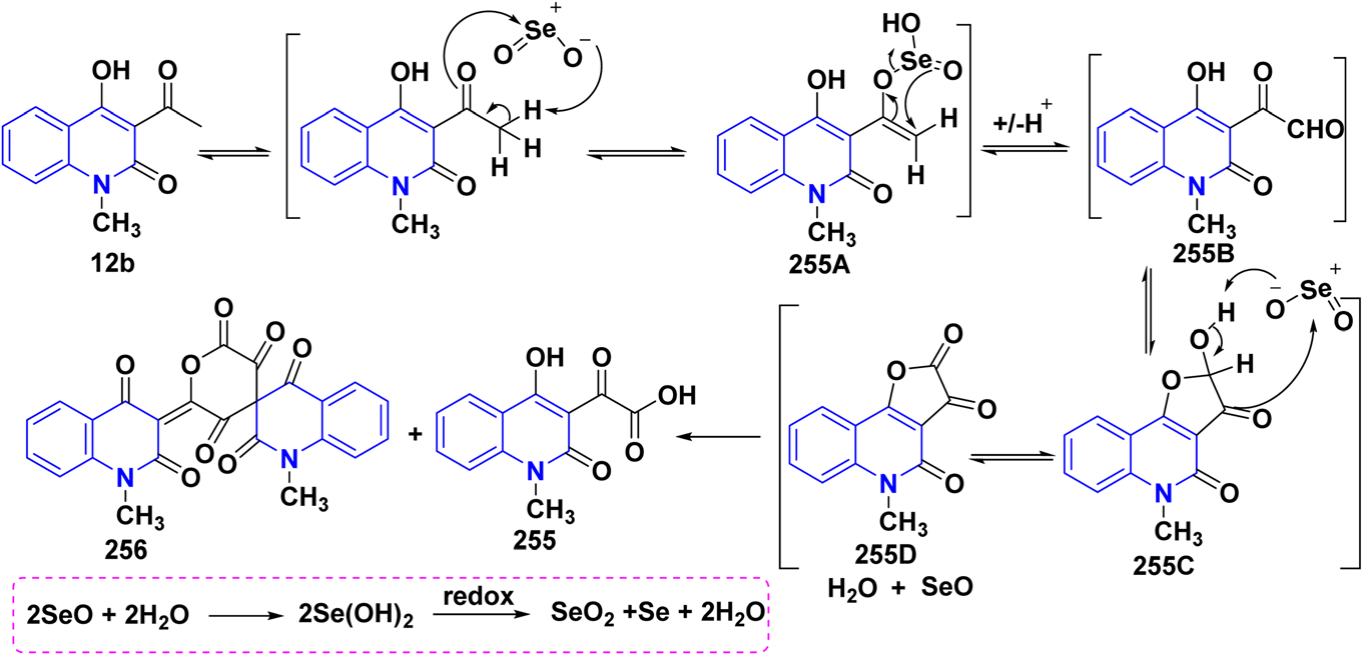
Proposed mechanism for the synthesis of 255 and 256.

A probable mechanism for the synthesis of the *α*-keto acids 255 can be simply explained by the fast oxidation of quinolone derivatives 12b,d to *α*-keto aldehyde intermediates 255A, which cyclize into the hemiacetal intermediates 255B, which can afford the corresponding 5-alkylfuro[3,2-*c*]quinolinetriones 255C. These furoquinolinetriones 255C and 255D are easily hydrolyzed, furnishing the formation of 255 as a major product and 256 as a minor product (Scheme 97).

**Scheme 98:**
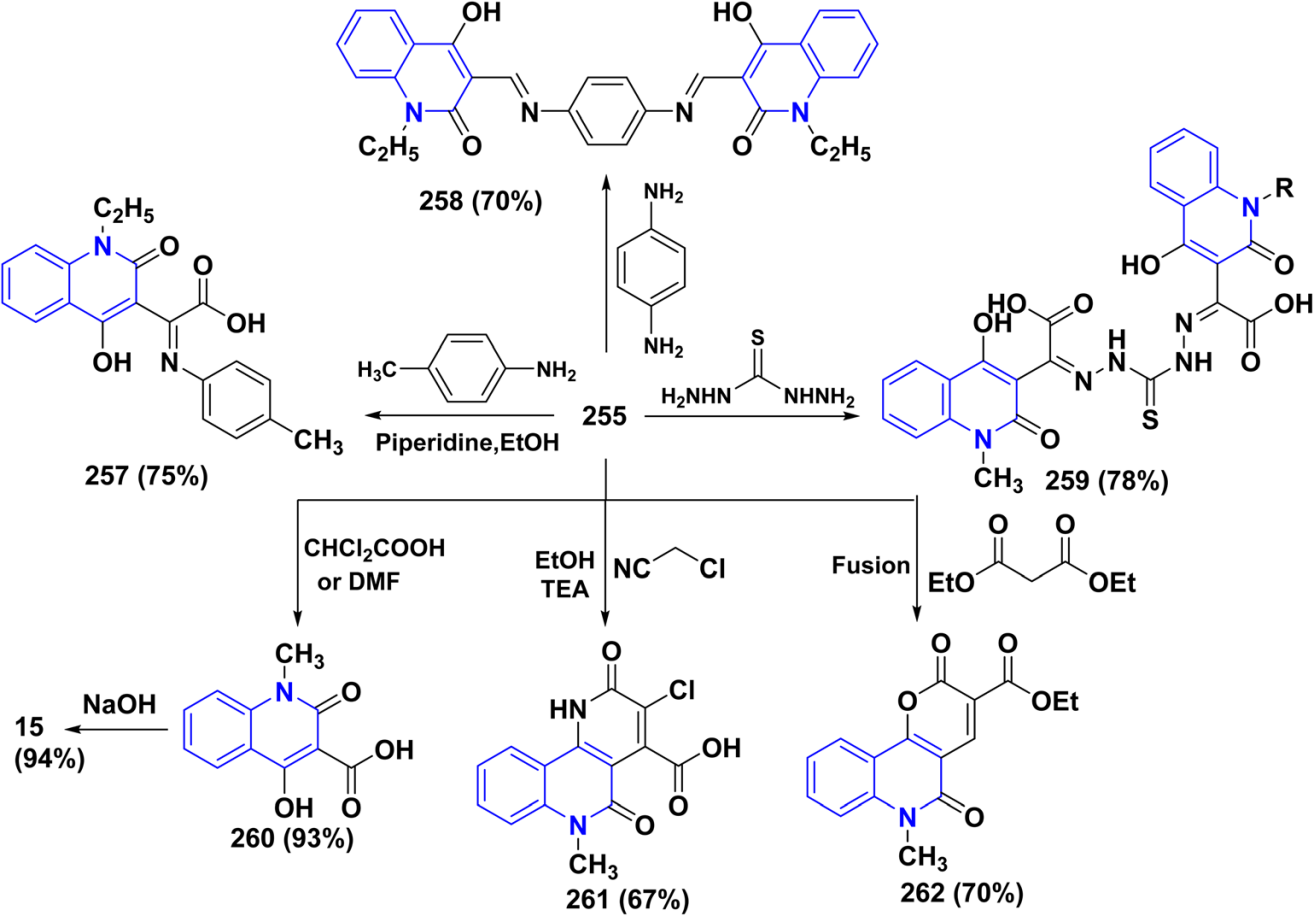
Reaction of *α*-keto acids 255 with different reagents.

Specifically, the reactivity of 255 with various nucleophilic reagents was investigated, as the reaction of 255 with *p*-toluidine in refluxing EtOH with a few drops of piperidine produced (4-hydroxy-2-oxoquinolin-3-yl)-*p*-tolyliminoacetic acid 257. Similarly, reaction of 255 with *p*-phenylenediamine in DMF yielded *bis*[(hydroxy-1,2-dihydroquinolinyl)methylidene]-1,4-phenylenediamine 258. While condensing *α*-ketoacid 255 with thiocarbohydrazide in different molar ratios in boiling EtOH containing TEA afforded *bis*[2-(4-hydroxy-2-oxoquinolin-3-yl)-2-oxoacetic acid]thiocarbohydrazone 259. Additionally, the reaction of *α*-ketoacid 255 with various carbon nucleophiles was explored through its reaction with active methylene such as dichloroacetic acid, chloroacetonitrile, and diethyl malonate, which led to the synthesis of quinoline-3-carboxylic acids 260 and fused tricyclic scaffolds (3-chloro-2-hydroxymethylbenzo) carboxylic acid 261 and 2,5-dioxopyrano[3,2-*c*]quinoline 262, respectively. Whereas, the hydrolysis process of quinoline-3-carboxylic acids 260 by NaOH was continued for a day, yielding 4-hydroxy-2-oxoquinoline 15 (Scheme 98).^208^

#### 4.4.3. Reduction of AHQ

**Scheme 99:**
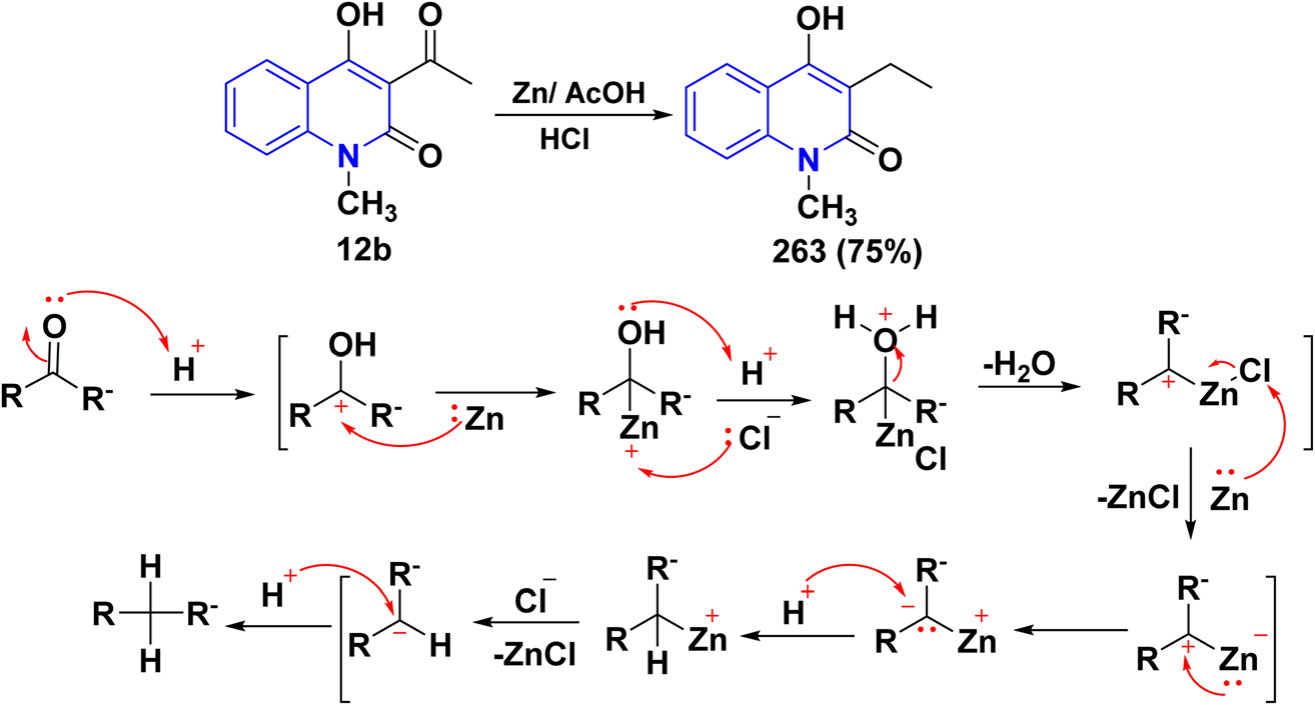
Reduction of the acetyl group of 12b to form compound 263, in addition to the plausible mechanism of the Clemmensen reduction.

Whereas, the reduction is one of the most effective chemical transformation processes in organic chemistry^209^ as the development in this field has been tremendous, progressing from the use of stoichiometric reagents to get novel organic systems, whereas, Clemmensen-type reduction of acetylquinolinone 12b in the presence of zinc dust (particle size <45 μm) afforded 3-ethyl-4-hydroxyquinolinone 263 in acceptable yield 75% (Scheme 99).^210^ The Clemmensen reduction is a chemical reaction used to convert a carbonyl group (C=O) into a methylene group directly. Firstly, the carbonyl group is protonated by acidic hydrogen to be more electrophilic and easily attacked. Then, nucleophilic Zn attacks the protonated carbocation, leading to the formation of a tetrahedral carbinol containing the carbon–zinc bond, which is protonated by HCl through a carbenoid mechanism. After that, the reduction process happens on the surface of the zinc metal, leading to the formation of the corresponding alkane through undergoing two sequential 2e^−^ reduction steps involving a dehydration step and anionic intermediates (Scheme 99).^211^

**Scheme 100:**
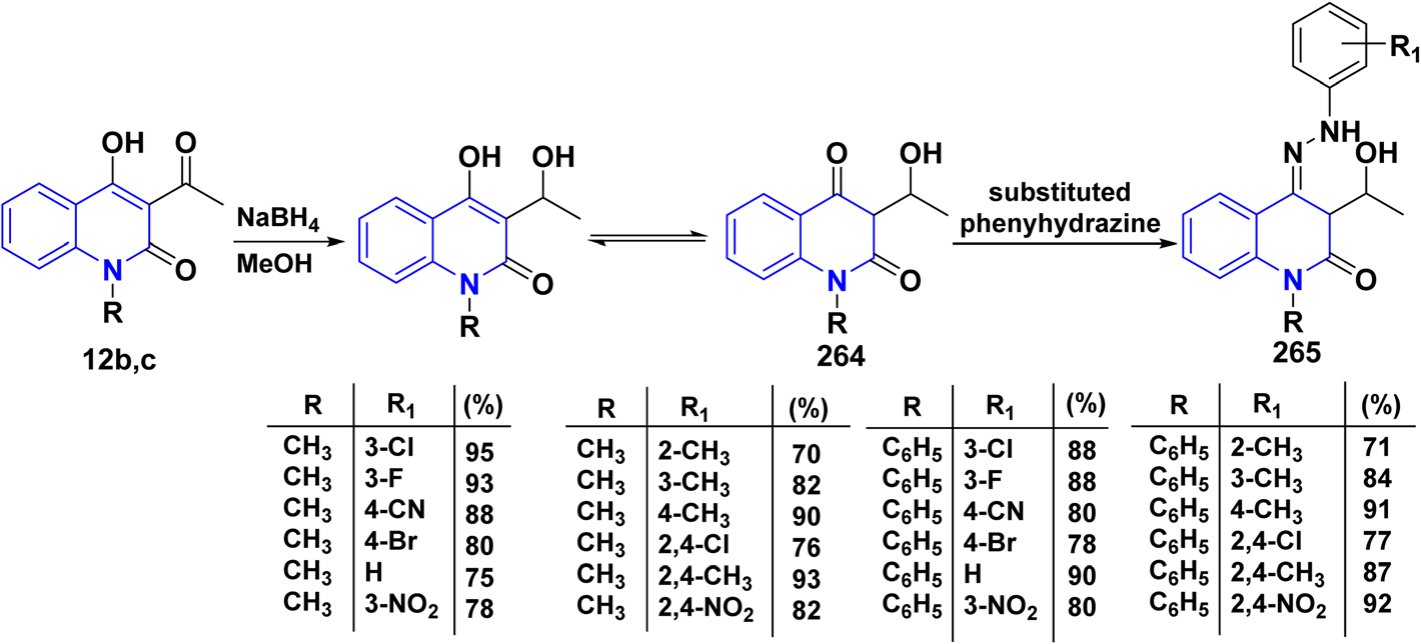
Reduction of compounds 12b,c using NaBH_4_.

Reduction of derivatives 12b,c*via* using sodium borohydrate (NaBH_4_) as hydrogen source and electron donor afforded 4-hydroxy-3-(hydroxyethyl)quinolinones 264. Whereby, a chemoselective reduction of 264 was achieved by the treatment of 264 with substituted phenylhydrazines, yielding hydrazono-3-(1-hydroxyethyl)quinolin-2-one derivatives 265 (71–92%) (Scheme 100).^212^

## 5. Applications

### 5.1. Medicinal perspective of quinolinones

**Fig. 6:**
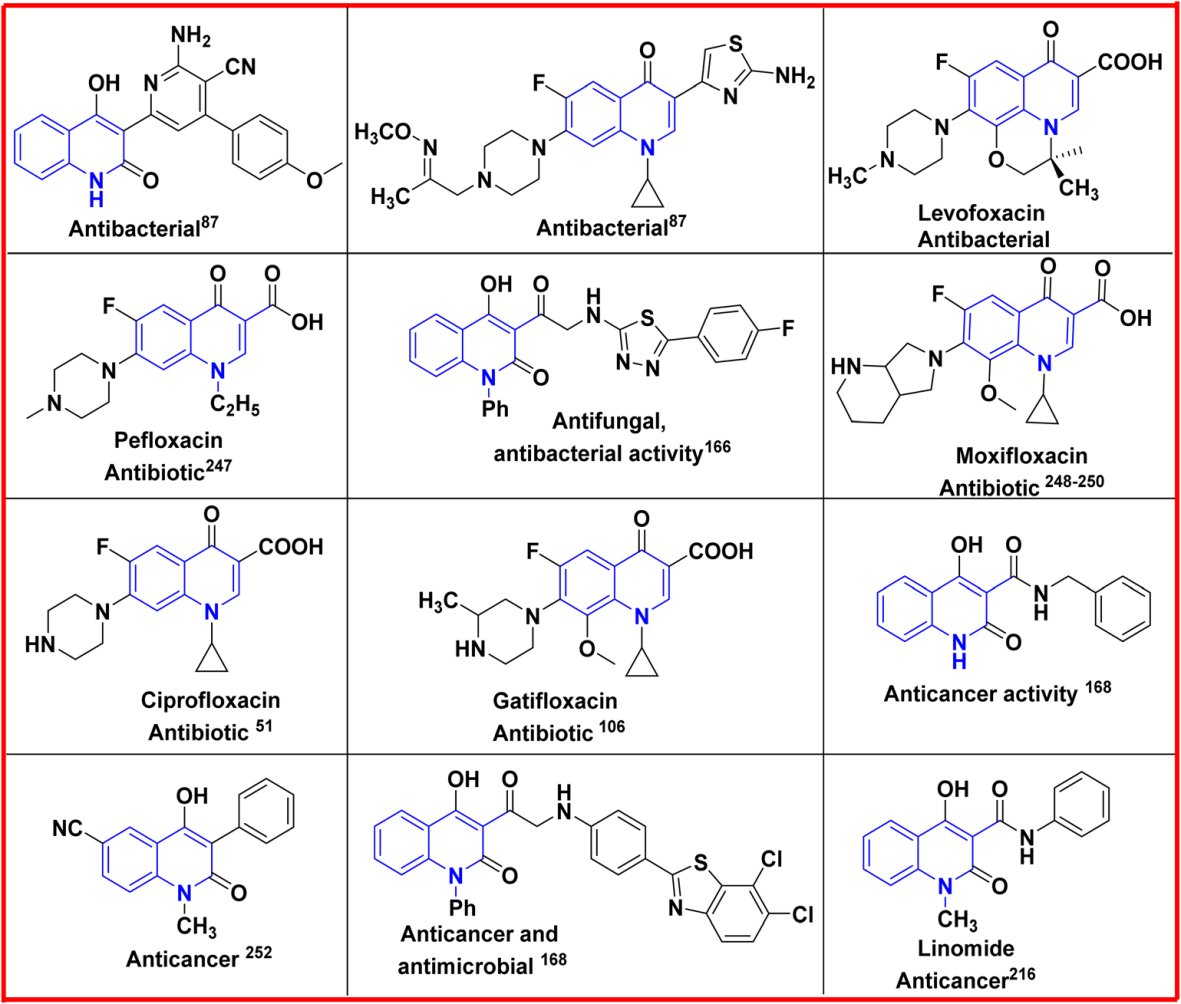
Biologically active compounds containing quinolinone scaffolds.

Quinolinone derivatives are significant heterocyclic systems with multiple medicinal applications.^84^ As these compounds possess various pharmacological properties,^213,214^ including analgesic effects,^106,215^ anti-inflammatory,^108,216–219^ antiallergenic,^107,220,221^ diuretic,^222,223^ cardiovascular agents,^47,224^ orally active antagonists,^225–228^ antimicrobial,^161,229–232^ anticonvulsant,^110,233–235^ acetylcholinesterase reactivators,^236–239^ antitumor, anticancer,^136,240^ Farnesyl transferase inhibitor,^241^ antioxidant,^107,192,216,242^ anti-tubercular activity^106,243–246^ and other potential biological applications ^247–252^ as shown in Fig. 6.

**Fig. 7:**
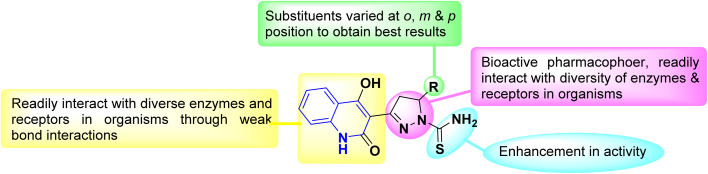
Design an approach to increase anti-tuberculosis activity.

Globally, tuberculosis (TB) is a highly lethal infectious disease caused by the bacterium *Mycobacterium tuberculosis*, which is responsible for approximately three million deaths. The recent report from the World Health Organization (WHO) identifies TB as the second leading cause of death from infectious diseases worldwide. About one-third of the global population is at risk due to latent infections with *Mycobacterium tuberculosis*, according to the WHO.^106,253,254^ On the other hand, quinolines are a crucial active pharmaceutical ingredient that plays a vital role in discovering new drug candidates. Many quinoline-based compounds are currently in clinical and preclinical development for tuberculosis treatment. Additionally, several quinoline-derived medications, such as moxifloxacin, gatifloxacin, and TMC207, are utilized to treat tuberculosis.^255–257^ Interestingly the incorporation of pyrazole derivatives into the quinolinone scaffold enhances its biological efficacy, as it alters modes of action, improves selectivity profiles, and reduces unwanted side effects. Those compounds may be further changed to have better pharmacokinetics and oral bioavailability (Fig. 7).^106,258^

**Fig. 8:**
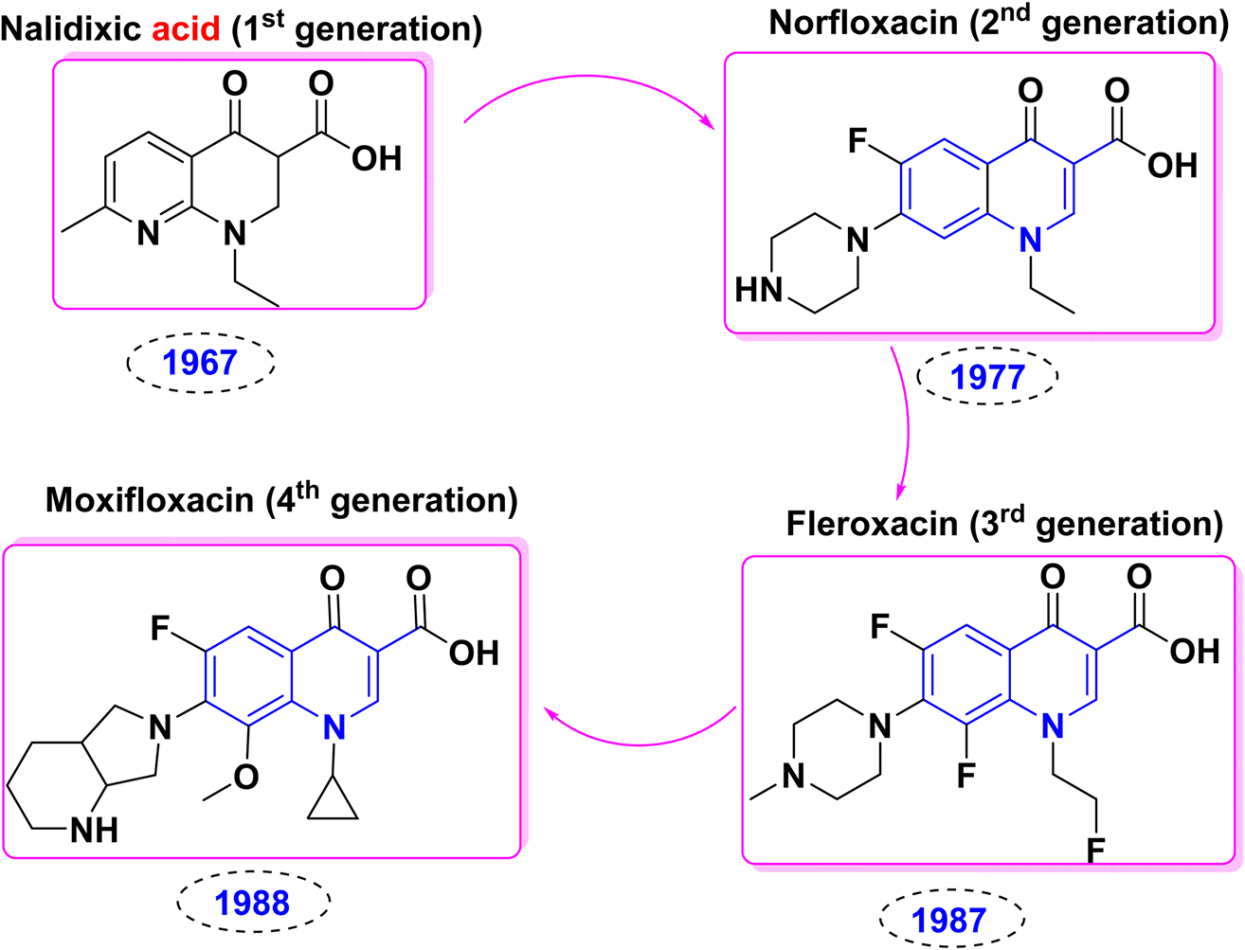
The history of quinolinones and modifications to improve their pharmacokinetics.

The accidental discovery of nalidixic acid during chloroquine synthesis and its role in the development of numerous quinolinone analogues, including flumequine, rosoxacin, ofloxacin, ciprofloxacin, moxifloxacin, levofloxacin, trovafloxacin, and marbofloxacin. All of the aforementioned pharmaceutical candidates demonstrated significant antibiotic activities.^259–261^ Over the years, the history of quinolones is characterized by numerous iterations, innovations, and expansions, as evidenced by the many potent drugs available today. In 1962, nalidixic acid (1st generation) was discovered and then approved in 1967 for treating uncomplicated urinary tract infections (UTIs), but resistance quickly developed among various species.^262^ Due to adverse bioeffects as low serum concentrations and high minimum inhibitory concentrations, nalidixic acid was largely abandoned till the emergence of fluoroquinolones in the 1970s and 1980s.^263,264^ Modifying quinolones to fluoroquinolone scaffolds improved their pharmacokinetics and expanded their antimicrobial spectrum.^263,264^ Interestingly, certain second-generation marketed antibiotics, such as ofloxacin, ciprofloxacin, and norfloxacin, are still in use today (Fig. 8).^250^

Fleroxacin signifies the start of the “3rd generation” of quinolinones, which demonstrated meaningfully enhanced antimicrobial activity due to various modifications in its chemical structure, as shown to be photocarcinogenic and photomutagenic.^265^ Finally, Moxifloxacin, the “4th generation” of quinolones, is characterized by robust antimicrobial activity, including high efficacy against pathogens due to improved anaerobic coverage.^250,263,266,267^

**Fig. 9:**
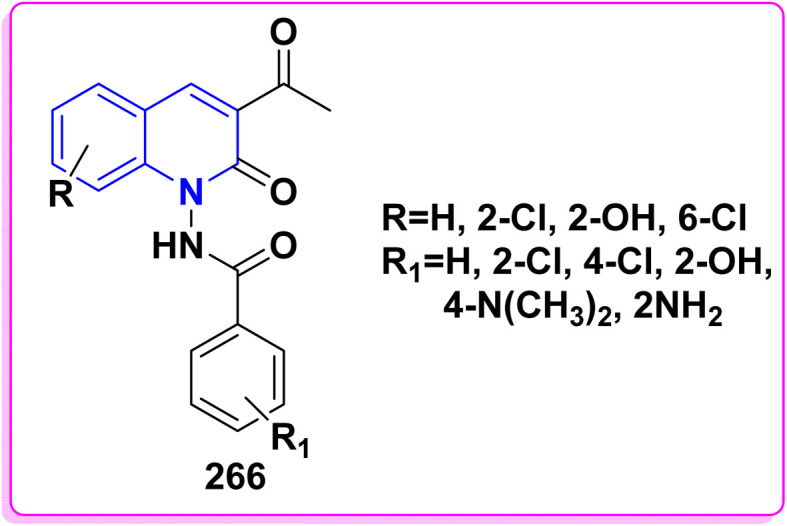
Quinolinone scaffolds with biological efficacy.

Also synthesized *N*-(3-acetyl-2-oxoquinolin-1(2*H*)-yl)benzamide derivatives 266 were tested for their antitubercular and antimicrobial activities. The majority of the tested derivatives exhibited encouraging antitubercular efficacy in comparison to the standard drugs (isoniazid and streptomycin). The inclusion of electron-donating groups such as methyl, amino, hydroxy, and dimethylamino has enhanced its antitubercular activity. Notably, most of the investigated derivatives of 266 displayed considerable antibacterial efficacy against both Gram-positive and Gram-negative microorganisms. They also showed marked antifungal efficacy against *C. albicans* and *A. niger* (Fig. 9).^268^

**Fig. 10:**
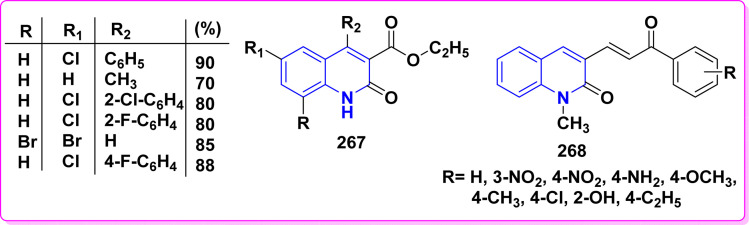
Quinolinone skeletons with antibacterial activity.

Krishnakumar *et al.*^269^ reported the evaluation of *in vitro* antibacterial properties for ethyl-2-oxoquinoline-3-carboxylate 267, showing moderate activity against the *Vibrio cholerae* and *Bacillus subtilis*. Likewise, 1-methyl-3-(3-oxo-3-phenylprop-1-enyl) quinoline-2-(1*H*)-ones 268 were examined *in vitro* as antimicrobial agents exhibiting significant antibacterial activity against *S. Typhi*,*S. aureus*, *P. aeruginosa*, and *B. subtilis* (Fig. 10).^270^

**Fig. 11:**
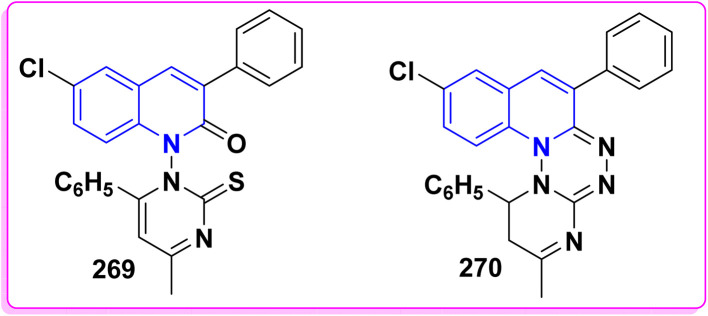
Quinolone-pyrimidine based molecular hybrids as potential anticancer agents.

Whereas, the next generation of anticancer, antimicrobial, and anti-HIV-1 drugs is expected to be characterized by quinolinone-pyrimidine-based. Anticancer evaluation of pyrimidoquinolinone 269 and pyrimidotetrazinoquinoline 270 was examined *in vitro* against the human liver cancer cell line (HEPG2). The anticancer activity results indicated that these compounds showed inhibitory activity against the tested cell line with IC_50_ values of 38.30 μM for 269 skeleton and 39.8 μM for 270 (Fig. 11).^271^

**Fig. 12:**
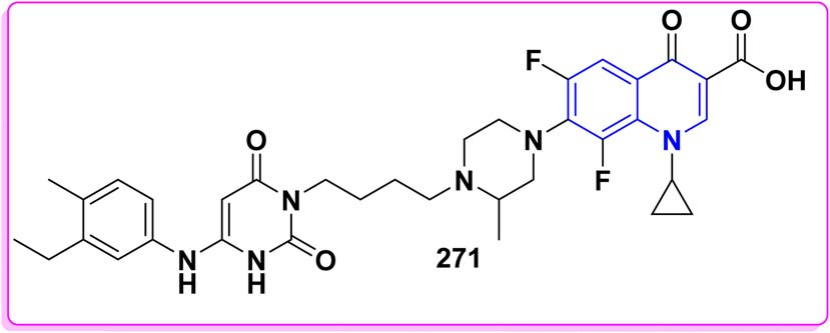
Quinolone-pyrimidine based molecular hybrids as potential antibacterial agents.

Compound anilinouracil-fluoroquinolone 271 demonstrated Gram-positive antibacterial potency at least 15 times that of the corresponding [3-(4-hydroxybutyl)-6-(3-ethyl-4-methylanilino)uracil], effectively inhibited pol IIIC and topoisomerase/gyrase, inhibited gyrase and *B. subtilis* topoisomerase IV with IC_50_ of 31 and 43.6 μM, respectively, and, as anticipated, had a selective effect on bacterial DNA. Additionally, this compound showed the ability to attack both of its possible targets in the bacterium by being active against a wide range of Gram-positive pathogens that were resistant to antibiotics, as well as a number of Gram-negative organisms. It was also active against Gram-positive organisms that were resistant to fluoroquinolones and anilinouracils. Moreover, it lacked toxicity *in vitro* and was bactericidal for Gram-positive bacteria. With a unique dual mechanism of action and strong activity against Gram-positive bacteria that are both susceptible to and resistant to antibiotics, this class of anilinouracil–fluoroquinolone hybrids offers a promising new pharmacophore (Fig. 12).^272^

**Fig. 13:**
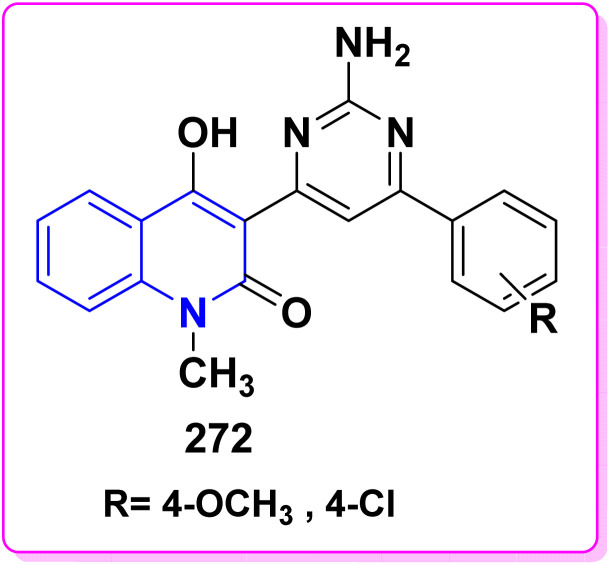
Pyrimidinylquinolinones based molecular hybrids as potential anticancer agents.

Furthermore, the synthesized derivatives of 3-(2-amino-6-arylpyrimidin-4-yl)-4-hydroxy-1-methylquinolin-2(1*H*)-ones 272 had shown the best activity in the series, with anticancer efficacy against HepG2 cell lines (IC_50_ values of 1.32 and 1.33 μM, respectively) (Fig. 13).^131^

### 5.2. Agricultural applications

In recent years, the discovery of agrochemicals based on the quinoline scaffold structure has led to great progress. Some new quinoline pesticides that have been commercialized or are under development not only inject new vitality into the market but also can replace unfriendly old pesticides.^51^ For example, ethyl-4-hydroxy-1-(4-methoxyphenyl)quinolinone-3-carboxylate 273, has been investigated for the first time as a sensitization chromophore for Tb(iii) to improve selectivity and sensitivity for organophosphorus pesticide detection.^273^

**Fig. 14:**
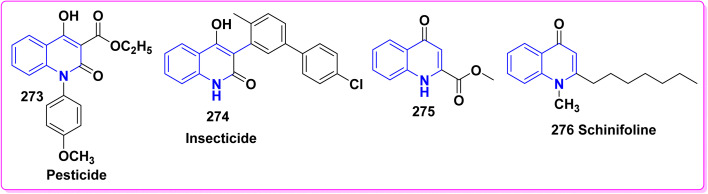
Agrochemical compounds with quinolinone scaffolds.

The insecticidal activity of pyrazoloquinolone compounds was investigated, both *in vitro* and *in vivo*, against the cotton leafworm, *S. littoralis*, and cotton aphids. The most effective compounds were (*E*)-4-(2-(dimethylamino)phenyl)-2,5-dioxo-6-phenyl-1,2,5,6-tetrahydrobenzo[*h*][1,6] naphthyridin-3-carbonitrile 203 and 3-(1-(4-amino-5-mercapto-4*H*-1,2,4-trizol-3-yl)-1*H*-pyrazol-3-yl)-4-hydroxy-1-phenylquinoline-2(1*H*)-one 200, with LC_50s_ of 119.79 and 164.63 mg L^−1^ against *S. littoralis*.^192^ Additionally, 1,4-dihydro-quinolinecarboxylate 275 is isolated from *Beauveria* sp. for the first time and has insecticidal activity against *Bemisia tabaci* with remarkable toxicity in contact and feeding assays, as its LC_50_ values were 10.59 μg mL^−1^ (contact) and 5.66 μg mL^−1^ for feeding. Whereas, no adverse effect on plant height/growth or phytotoxicity was detected on pepper, tomato, cotton, and cucumber during the treatment.^274^ Also, Liu *et al.*^275^ reported that the utilization of Schinifoline 276, the fruit pericarp of *Zanthoxylum schinifolium* possessed remarkable feeding toxicity against *Sitophilus zeamais* and *Tribolium castaneum* through reducing their food consumption and growth rate, leading to weakness of their reproductive ability and low resistance (Fig. 14).^275^

**Fig. 15:**
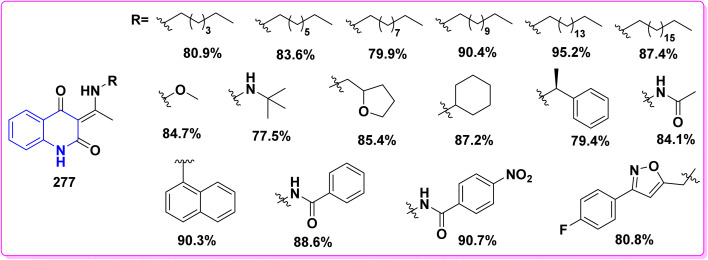
Quinolinone scaffolds with agrochemical efficacy.

Additionally, a series of (*E*)-3-acyl-quinoline-2,4-(1*H*,3*H*)-dione imine derivatives 277 displayed remarkable herbicidal activity against monocot and dicot species by affecting PSII electron transport inhibitors and inhibiting the electron transport chain, leading to the prevention of the production of NADPH and ATP. As the majority of the compounds showed good to excellent herbicidal activities against a number of dicot or monocot species, with an inhibition percentage of over 50% at a dosage of 94 g per ha or even lower. Notably, the compounds featuring short straight-chain alkyl groups display the highest activity, comparable with the longer alkyl chains; insecticidal activity diminishes with increasing chain length (Fig. 15).^276^

**Fig. 16:**
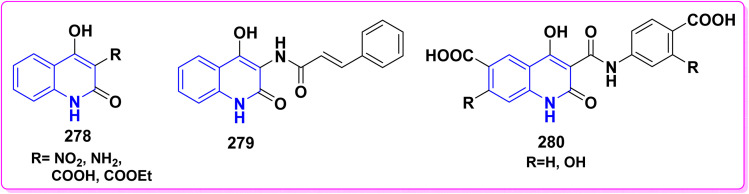
Quinolinone scaffolds with photosynthesis-inhibiting activity.

A series of substituted 4-hydroxy-1*H*-quinolin-2-one derivatives 278–280 were analyzed using RP-HPLC to determine lipophilicity and photosynthesis-inhibiting activity (the inhibition of photosynthesis in *Spinacia oleracea* L. As the synthesized quinolinones displayed high to moderate inhibitory effects on the photosynthesis activity process. In addition, *in vitro* antifungal screening of these scaffolds was evaluated against various fungal strains, displaying moderate efficacy (Fig. 16).^277^

**Fig. 17:**
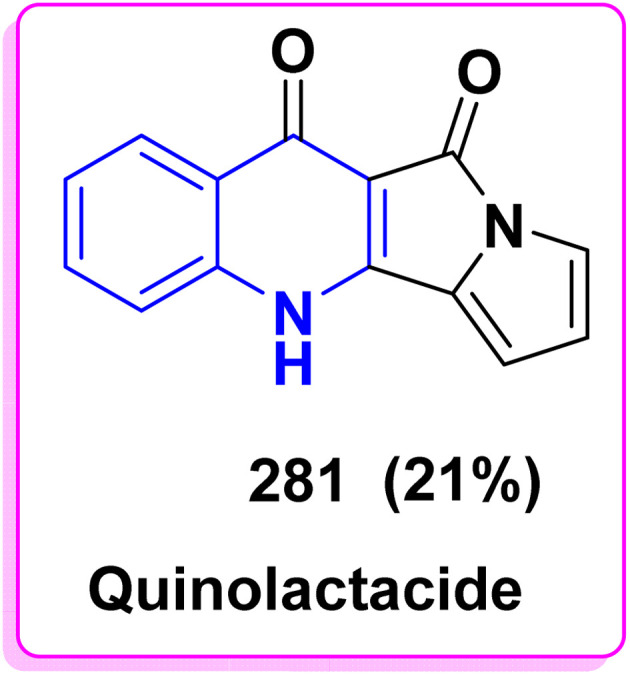
Quinolinone scaffolds with insecticidal activity.

Quinolactacide 281 is structurally analogous to quinolactacins derived from *Penicillium* species, which are recognized for exhibiting various biological activities, particularly insecticidal properties. This compound exhibited 88% mortality against the green peach aphid (*Myzus persicae*) at a concentration of 250 ppm. The insecticidal and miticidal efficacy of quinolactacide was assessed against five distinct insects and one mite at a concentration of 500 ppm. The insects utilized for the experiments included the green peach aphid, silverleaf whitefly (*Bemisia argentifolii*), diamondback moth (*Plutella xylostella*), common cutworm (*Spodoptera litura*), western flower thrips (*Frankliniella occidentalis*), and two-spotted spider mite (*Tetranychus urticae*). The synthetic quinolactacide exhibited no activity against the other insects, but it demonstrated 100% and 42% mortality against the green peach aphid and diamondback moth, respectively (Fig. 17).^278^

### 5.3. Complex formation

**Fig. 18:**
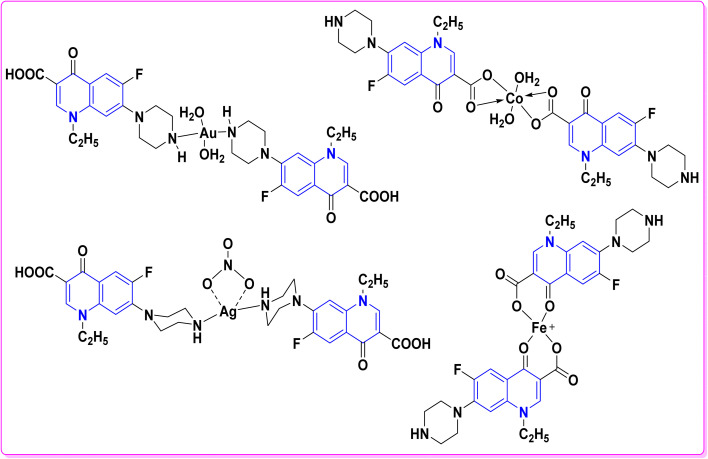
Coordination modes of quinolinone complexes with high biological activity.

Quinolinones bind metal ions to create complexes where they can function as bidentate, unidentate, or bridging ligands. Quinolinone molecules have a basic side nucleus that becomes protonated and shows up as cations in the ionic complexes under highly acidic conditions. Quinolone solubility, pharmacokinetics, and bioavailability are all significantly impacted by interaction with metal ions, which also plays a role in the bactericidal agents' mode of action. Many metal complexes have shown various biological activities, including antifungal, anticancer, antiparasitic, and antimicrobial (Fig. 18).^279^

**Fig. 19:**
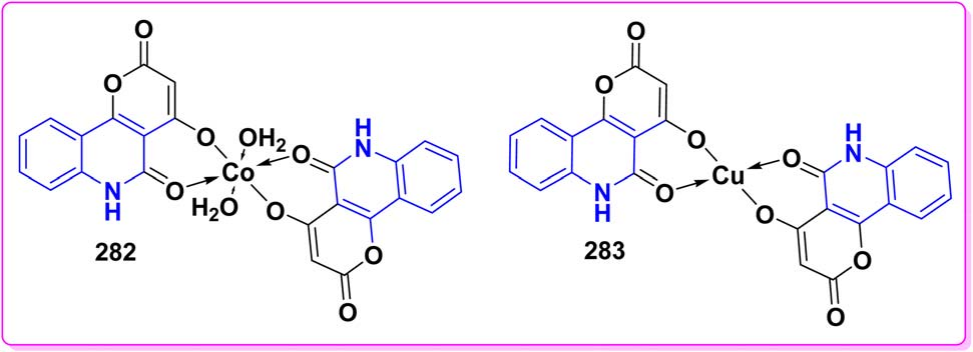
Bioactive complexes with quinolinone scaffolds.

A series of Cu(ii) 282 and Co(ii) 283 complexes were designed and synthesized by Khalaf *et al.*^280^ as 4-hydroxy-2*H*-pyrano[3,2-*c*]quinolin-2,5(6*H*)-dione acts as monobasic didentate ligand and form tetrahedral and octahedral complexes at molar ratio of 1 : 2. Interestingly, the synthesized complexes displayed greater efficacy as antibacterial, antifungal and antioxidant candidates in comparison with the free pyrano[3,2-*c*]quinolin-2,5(6*H*)-dione as ligand (Fig. 19).^280^

**Fig. 20:**
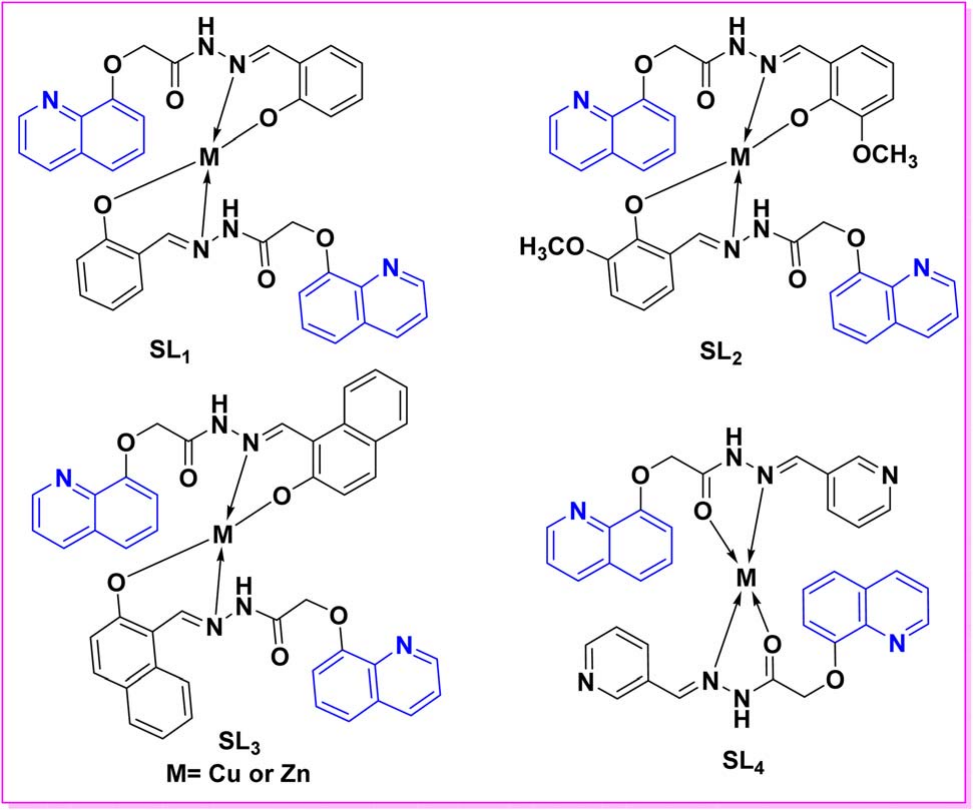
Hydroxyquinoline functionalized Schiff base copper and zinc complexes SL_1–4_.

The potential antibacterial and antifungal properties of the quinoline ligands (SL_1_–SL_4_) and their Cu(ii) and Zn(ii) complexes (Fig. 20) were evaluated *in vitro*. The ligands (SL_1_–SL_4_) demonstrated moderate antibacterial activity against Gram-positive bacteria but exhibited no effectiveness against Gram-negative bacteria or fungal strains. Among them, the SL_3_ ligand displayed the highest activity, achieving a zone of inhibition of 24 mm against *S. aureus.* However, the antibacterial activity of these ligands was significantly lower than that of the standard drugs amoxicillin and fluconazole. These metal complexes generally enhanced antibacterial activity against both Gram-positive *S. aureus* and *E. faecalis* strains. On comparing the metal complexes, it was found that [Cu(SL_1_)_2_] was the most harmful substance to Gram-positive bacteria and had moderate antifungal properties.^281^

**Fig. 21:**
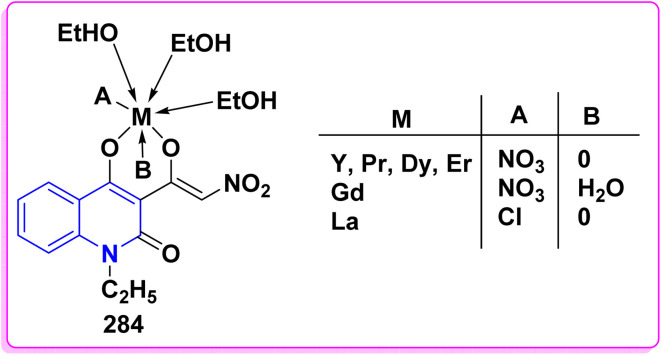
The chemical structures of lanthanide complexes.

Fouad *et al.*^282^ studied lanthanide nano-complexes (Gd^3+^, Er^3+^, Pr^3+^, Y^3+^, Dy^3+^, and La^3+^) containing quinolinone. The obtained results reveal that the quinolinone 284 acts as a bidentate *via* OO donor sites, creating octahedral complexes. The complexes are nanoscale, having crystalline or amorphous structures. The synthesized nano-complexes have acceptable anticancer efficacy against the hepatocellular carcinoma cell line. Whereby, the maximum anticancer activity of Pr^3+^ nano-complex (Fig. 21).^282^

**Scheme 101:**
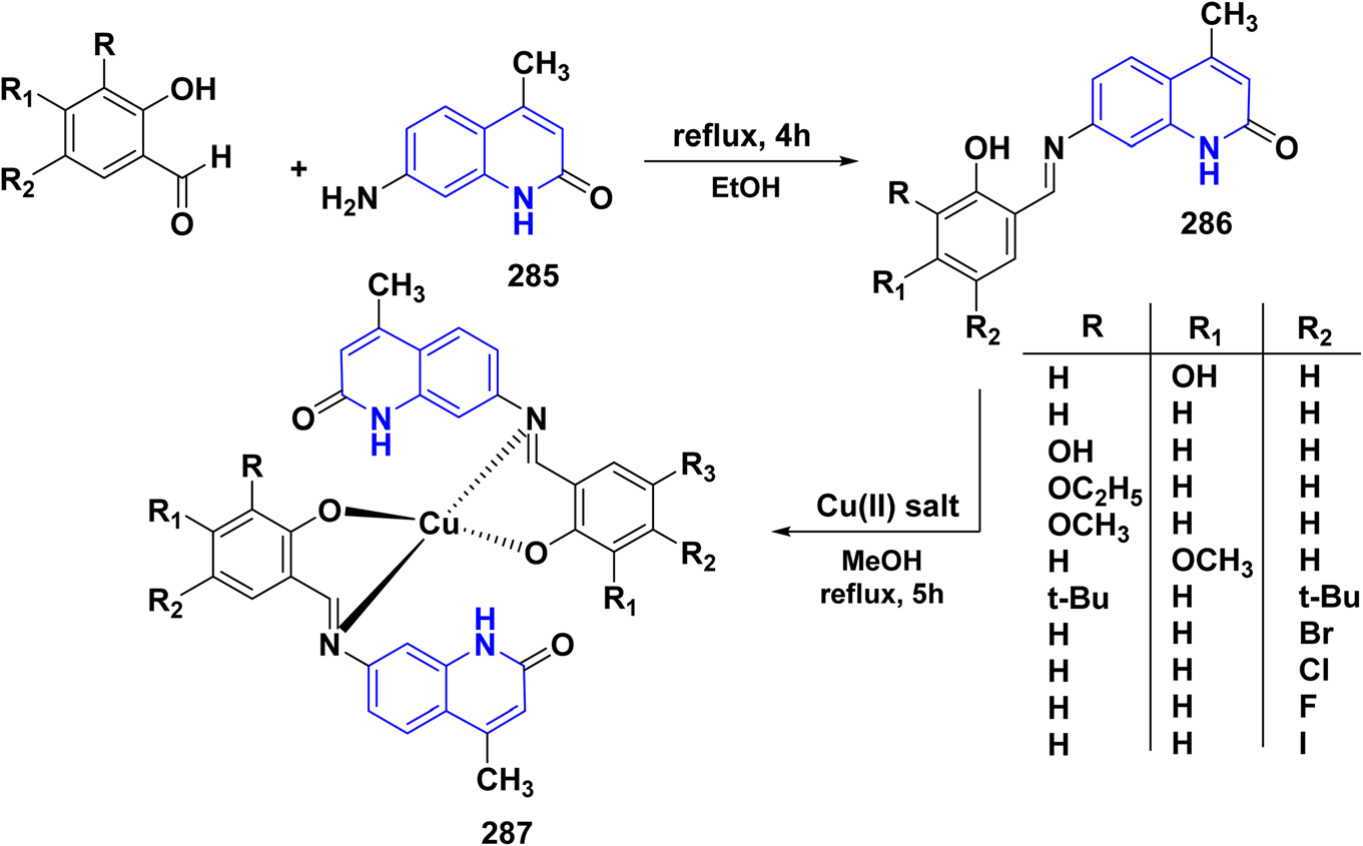
Complexation of ligand 286 with Cu(ii) salts.

A series of quinolinone-derived Schiff bases 286 were synthesized *via* the condensation of 7-amino-4-methylquinolinone 285 with different aromatic aldehydes. Then, complexation of Schiff ligands with cupric salts involving Cu(ii) acetate or Cu(ii) perchlorate hexahydrate was achieved, affording Cu(ii) complexes of the corresponding ligands 287 (Scheme 101). These complexes showed significant antifungal effects, suppressing *C. albicans* growth by 50% at a dosage of 4 μM.^283^

### 5.4. Corrosion inhibitors

**Fig. 22:**
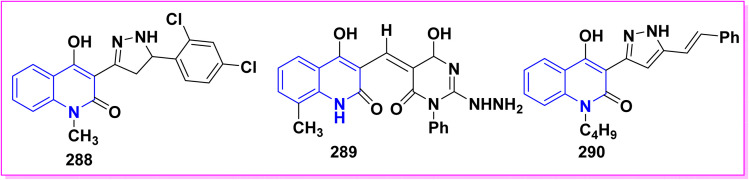
Quinolinone scaffolds with antioxidant effect in lubricating oils.

Hussein *et al.*^185^ reported the utilization of 4-hydroxyquinolinone derivatives as antioxidant agents for various lubricating oils through using the ASTM D-942 and ASTM D-664 tests. The compositions that they synthesized are: dichlorophenyl-pyrazol-4-hydroxy-1-methylquinolinone 288, 4-hydroxy-8-methyl-2-oxoquinolin-1-phenyl-2-hydroxyhydropyrimidin-4,6-dione 289, and 1-butyl-4-hydroxy-3-1*H*-pyrazol-3-yl)quinolinone 290 (Fig. 22). The obtained results indicate that the hydroxyquinolinone scaffolds reduce both the total acid number and the oxygen pressure drop in lubricating oils. On the other hand, it has been discovered that the antioxidant activity is most efficient when quinolinones have both butyl and hydroxyl groups. These quinoline scaffolds (288–290) inhibit radical processes during oil oxidation, as well as their continuation and production. A comparison of the antioxidant activity of compounds 288, 289, and 290 reveals that the efficacy and antioxidant benefits of the third compound.^184,284^

**Fig. 23:**
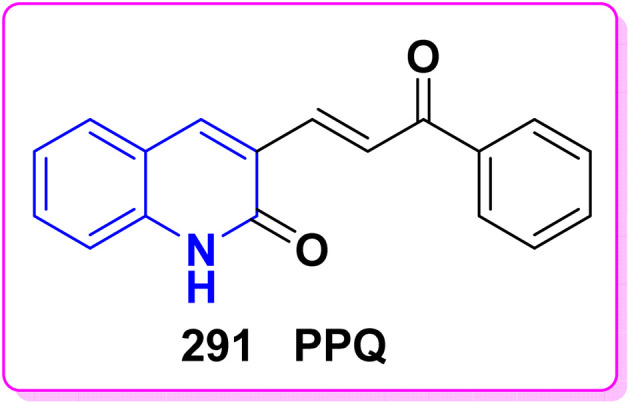
Quinolinone scaffolds as corrosion inhibitors.

Quinolinone chalcone (PPQ) 291 showed effective corrosion inhibition for high carbon steel (HCS) in 1.0 M HCl, and the inhibition efficiency improved as the inhibitor concentration was raised. The compound's efficiency is attributed to the presence of heteroatoms and phenyl rings with delocalized π electrons that can act as adsorption centers. These factors support both physical and chemical interactions and adsorption of inhibitor scaffolds with the metal surface according to the Langmuir adsorption isotherm (Fig. 23).^285,286^

**Fig. 24:**
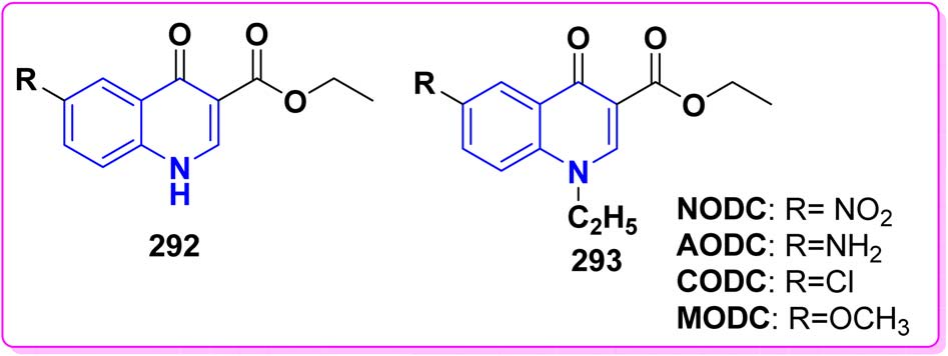
Quinolinones scaffolds as potential corrosion inhibitors.

The dihydroquinoline-3-carboxylate derivatives 292 and 293 (NODC, AODC, CODC, and MODC) display potential corrosion inhibition for carbon steel in 1 M HCl, due to the presence of a substituent attached to the benzene portion of the 4-quinolinone (Fig. 24).^287^

**Fig. 25:**
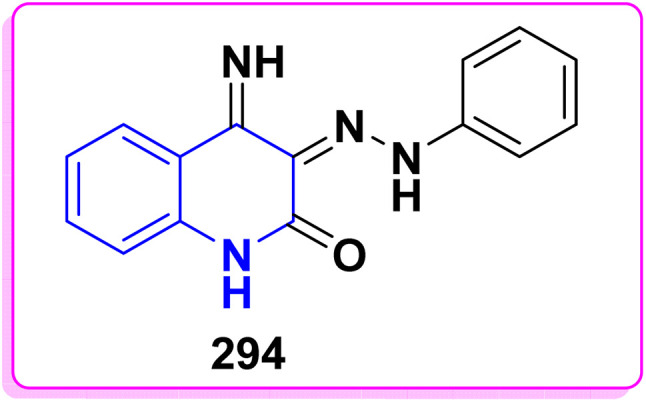
Imino quinolinone IQ 294.

Imino quinolinone IQ 294 acts as a corrosion inhibitor on low carbon steel (LCS) in 0.5 M HCl solution with inhibition efficiency 93.2% at the optimal concentration of IQ (30 × 10^−3^ Mm), adhered to the Langmuir adsorption model, signifying a monolayer adsorption mechanism. The characterization of IQ as a mixed-type inhibitor was evidenced due to its capacity to impede both cathodic and anodic processes, predominantly functioning as an anodic inhibitor (Fig. 25).^288^

### 5.5. Sensors

#### 5.5.1. Chemosensors

**Fig. 26:**
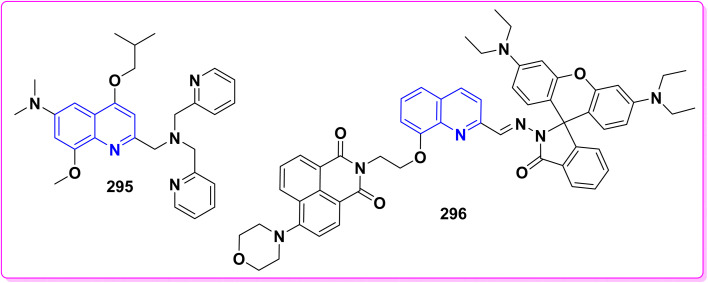
Quinolinone/hydroxyquinoline scaffolds as chemosensors.

Quinolinone derivative-based chemosensors designed to detect various metal ions were discussed. There is considerable interest in improving these chemosensors due to their ease of synthesis, high sensitivity, and stability. Nonetheless, there remains substantial potential for enhancement in their *in vivo* applications, particularly regarding water solubility, selectivity, and fluorescent bio-imaging capabilities. Consequently, the development of receptors tailored for different ions is essential. For instance, skeleton 295 employed an alternative bonding model to identify Zn^2+^ and Cd^2+^. Also, 296 act as a Cr^3+^ chemosensor. Additionally, two-photon laser sources can be used to excite an extended conjugated quinoline system (Fig. 26).^289^

#### 5.5.2. Fluorescence sensors

**Scheme 102:**
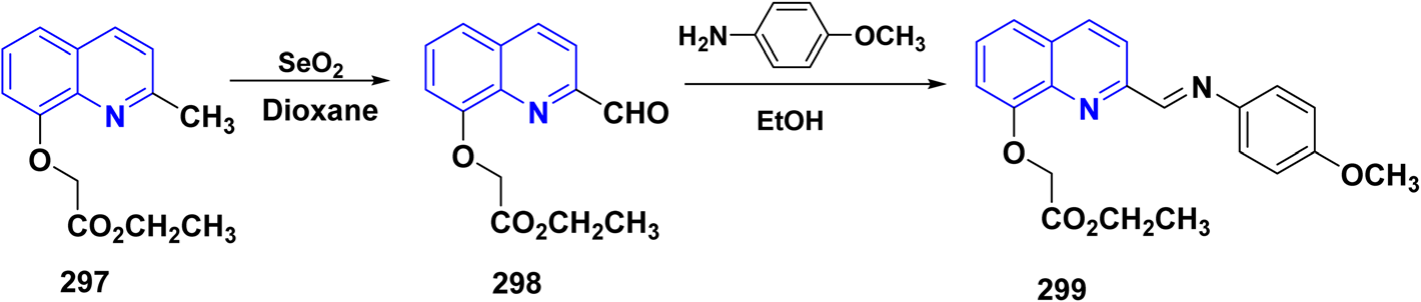
Hydroxyquinoline scaffolds as fluorescence sensors.

**Scheme 103:**
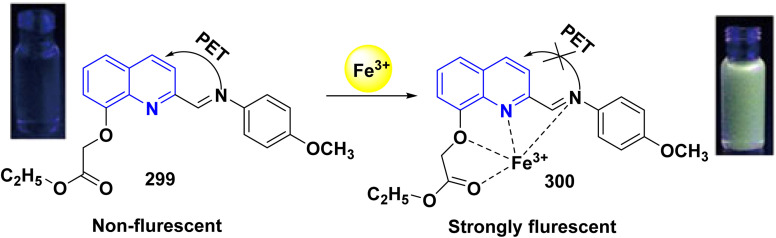
Complexation mechanism of sensor 299 and Fe^3+^ ions.

Fluorescence-based techniques have become essential tools in metalworking because of their sensitivity and selectivity benefits.^290–292^ A lot of work has gone into creating fluorescent Fe^3+^ indicators that are highly sensitive and selective. The majority of documented fluorescent sensors for Fe^3+^ are based on a fluorescence quenching mechanism due to the paramagnetic nature of Fe^3+^,^293,294^ and the majority of these have interference issues brought on by transition metal cations like Cu^2+^, Co^2+^, or Hg^2+^. Thus, looking for highly selective chemosensors for Fe^3+^ based on the fluorescence enhancement is crucial. While using the ether derivative 297 as a starting material, the synthesis of Schiff base 299 was utilized. Whereby, quinoline 299 is a chemosensor that made up of the quinoline fluorophore 298 and *p*-anisidine binding site led to formation of bearing polarized C–C, C–N and C–O bonds conjugated to the quinoline moiety, it displays a strong fluorescence increase of Fe^3+^ ions, demonstrating excellent sensitivity and good selectivity for Fe^3+^ over a broad spectrum of other ecologically and physiologically significant metal ions (Schemes 102 and 103).^295^

Sensor 299 is an excellent sensitive chemosensor for the Fe^3+^ ion as it exhibits no fluorescence in MeOH, but when Fe^3+^ is added, it affords a highly effective fluorescence sensor 300, and there is a noticeable increase in fluorescence of about 44-fold (Scheme 103). As a result, the fluorescence response is explained by Fe^3+^ blockage of electron transfer to the quinoline group, which encouraged sensor 299 to produce high fluorescence. A color shift from colorless to brown that is readily apparent to the unaided eye resulted from the identification of Fe^3+^. Consequently, the Fe^3+^ ion can be readily distinguished in visible light from all other metal ions. Additionally, under light from a 365 nm UV lamp, Fe^3+^ shows an increase in the intensity of fluorescence, vivid green, while other metal ions show no change under the same circumstances.^295^

## 6. Conclusion

With the ever-increasing importance of 3-acetyl-4-hydroxyquinolinone and its *N*-substituted derivatives as structurally decisive scaffolds in bioactive natural products and pharmaceutical drugs, so in this review enormous efforts have been made to summarize the synthesis, structural features and chemical reactivity of 3-acetyl-4-hydroxy-2-quinolinone entities and its *N*-substituted derivatives as structurally decisive scaffolds in bioactive natural products and pharmaceutical drugs, accredited by reaction mechanisms. Also, we highlight the most important breakthroughs of 3-acetyl-4-hydroxy-2-quinolinone derivatives as an auspicious class displaying a wide range of potential pharmacological activities and their developments in the various clinical stages. Generally, this review supplies an overview of the chemistry for 3-acetyl-4-hydroxy-2-quinolinone derivatives and documents more than two hundred references over the last decade of research, covering mainly the period from 2020 to the beginning of 2025.

## Abbreviations

AcOH: Acetic acid
AHQ: 3-Acetyl-4-hydroxyquinolinone
BQB: 1,4-*Bis*-(quinolin-6-ylimino methyl)benzene
BF_3_·(OC_2_H_5_)_2_: Boron trifluoride etherate
CS_2_: Carbon disulfide
CCl_4_: Carbon tetrachloride
DMF: Dimethylformamide
DMA: Dimethylamine
DMSO: Dimethyl sulfoxide
EAA: Ethyl acetoacetate
HCS: High carbon steel
NH_2_OH·HCl: Hydroxylamine hydrochloride
HMPT: Hexamethylphosphoric triamide
HQ: 4-Hydroxyquinoline
LCS: Low-carbon steel
LR: Lawesson's reagent
MCR: Multicomponent reaction
DMF/DMA: *N*,*N*-Dimethylformamide dimethyl acetal
DTA: *N*,*N*-Dimethyl trifluoroacetamide
NBS: *N*-Bromosuccinimide
POCl_3_: Phosphorous oxychloride
P_2_S_5_: Phosphorus pentasulfide
PBr_3_: Phosphorus tribromide
PhPOCl_2_: Phosphonic dichloride
PPA: Polyphosphoric acid
RORC: Ring opening and ring closure
RT: Room temperature
AcONa: Sodium acetate
SO_2_Cl_2_: Sulfuryl chloride
NaBH_4_: Sodium borohydrate
CH(OEt)_3_: Triethyl orthoformate
TEA: Triethylamine
P(OCH_2_CH_2_Cl)_3_: Tris(2-chloroethyl)phosphite
TB: Tuberculosis
TC: *β*,*β*-Tricarbonyl
TBAB: Tetrabutylammonium bromide
TsCl: Toluenesulfonyl chloride
WHO: World Health Organization
ZnCl_2_: Zinc chloride

## Data availability

No primary research results, software or code have been included, and no new data were generated or analyzed as part of this review.

## Conflicts of interest

The authors declare that they have no known competing financial interests or personal relationships that could have appeared to influence the work reported in this review.

## References

[cit1] Gulraiz A., Sohail M., Bilal M., Rasool N., Usman Qamar M., Ciurea C., Marceanu L. G., Misarca C. (2024). Molecules.

[cit2] Zhang X., Liu M., Qiu W., Zhang W. (2024). Molecules.

[cit3] Hamama W. S., Ibrahim M. E., Gooda A. A., Zoorob H. H. (2018). RSC Adv..

[cit4] Borah P. , Hazarika S., Chettri A., Sharma D., Deka S., Venugopala K. N., Shinu P., Al-Shar’i N. A., Bardaweel S. K. and Deb P. K., In Viral, Parasitic, Bacterial, and Fungal Infections, 2023, pp. 781–804

[cit5] Sabbah D. A., Samarat H. H., Al-Shalabi E., Bardaweel S. K., Hajjo R., Sweidan K., Abu Khalaf R., Al-Zuheiri A. M., Abushaikha G. (2022). ChemistrySelect.

[cit6] Rana M., Ranjan R., Ghosh N. S., Kumar D., Singh R. (2024). Curr. Cancer Ther. Rev..

[cit7] Ramanathan M., Moussa Z. (2025). Org. Chem. Front..

[cit8] Hamama W. S., Hassanien A. E., El-Fedawy M. G., Zoorob H. H. (2016). J. Heterocycl. Chem..

[cit9] Bhusare N., Kumar M. (2024). Oncol. Res..

[cit10] Kajal K., Shakya R., Rashid M., Nigam V., Kurmi B. D., Gupta G. D., Patel P. (2024). Sustain. Chem. Pharm..

[cit11] Bakr R. B., El Azab I. H., Elkanzi N. A. A. (2024). Chem. Biodivers..

[cit12] Darque A., Dumètre A., Hutter S., Casano G., Robin M., Pannecouque C., Azas N. (2009). Bioorg. Med. Chem. Lett..

[cit13] Dize D., Tali M. B. T., Ngansop C. A. N., Keumoe R., Kemgne E. A. M., Yamthe L. R. T., Fokou P. V. T., Kamdem B. P., Hata K., Boyom F. F. (2024). Future Pharmacol..

[cit14] Sun J., Kessl J. J. (2024). Viruses.

[cit15] Shi D., Xu S., Ding D., Tang K., Zhou Y., Jiang X., Wang S., Liu X., Zhan P. (2024). Expert Opin. Drug Discovery.

[cit16] Hamama W. S., Hassanien A. E., El-Fedawy M. G., Zoorob H. H. (2015). J. Heterocycl. Chem..

[cit17] Mısır B. A., Derin Y., Ökten S., Aydın A., Koçyiğit Ü. M., Şahin H., Tutar A. (2024). J. Biochem. Mol. Toxicol..

[cit18] Hammouda M. M., Rashed M. M., Abo El-Yazeed W. S., Elattar K. M. (2024). ChemistrySelect.

[cit19] Siddique S., Ahmad K. R., Nawaz S. K., Raza A. R., Ahmad S. N., Ali R., Inayat I., Suleman S., Kanwal M. A., Usman M. (2023). Sci. Rep..

[cit20] Kołodziej P., Wujec M., Doligalska M., Makuch-Kocka A., Khylyuk D., Bogucki J., Demkowska-Kutrzepa M., Roczeń-Karczmarz M., Studzinska M., Tomczuk K., Kocki M., Reszka-Kocka P., Granica S., Typek R., Dawidowicz A. L., Kocki J., Bogucka-Kocka A. (2024). J. Adv. Res..

[cit21] Das R., Mehta D. K., Gupta S., Mujwar S., Sharma V., Goyal A., Patel S., Patel A. (2023). Lett. Org. Chem..

[cit22] Abdel Aziz Y. M., Nafie M. S., Hanna P. A., Ramadan S., Barakat A., Elewa M. (2024). Pharmaceuticals.

[cit23] Costas-Lago M. C., Besada P., Mosquera R., Cano E., Terań C. (2024). Bioorg. Chem..

[cit24] Ramsis T. M., Ebrahim M. A., Fayed E. A. (2023). Med. Chem. Res..

[cit25] Siddique S., Hussain K., Shehzadi N., Arshad M., Ashad M. N., Iftikhar S., Saghir F., Shaukat A., Sarfraz M., Ahmed N. (2024). Org. Biomol. Chem..

[cit26] Hashem H. E., Ahmad S., Kumer A., El Bakri Y. (2024). Sci. Rep..

[cit27] Cebeci Y. U., Batur O. O., Boulebd H. (2024). J. Mol. Struct..

[cit28] Verma S. K., Rangappa S., Verma R., Xue F., Verma S., Kumar K. S. S., Rangappa K. S. (2024). Bioorg. Chem..

[cit29] Gangurde K. B., More R. A., Adole V. A., Ghotekar D. S. (2024). J. Mol. Struct..

[cit30] Jia Y., Zhao Y., Niu M., Zhao C., Li X., Chen H. (2024). Anim. Models Exp. Med..

[cit31] El Mesky M., Zgueni H., Rhazi Y., El-Guourrami O., Abchir O., Jabha M., Nakkabi A., Chtita S., Achamlale S., Chalkha M., Chebabe D., Mabrouk El. (2024). J. Mol. Struct..

[cit32] Swati, Raza A., Singh B., Pankaj Wadhwa Dr. (2025). ChemistrySelect.

[cit33] Fawzy M. A., Ibrahim K. H., Aly A. A., H Mohamed A., Abdel Hafez S. M. N., Abdelzaher W. Y., B Elkaeed E., A Alsfouk A., MN Abdelhafez El. (2024). Future Med. Chem..

[cit34] Mohassab A. M., Hassan H. A., Abou-Zied H. A., Fujita M., Otsuka M., Gomaa H. A. M., Youssif B. G. M., Abdel-Aziz M. (2024). J. Mol. Struct..

[cit35] Hamama W. S., Ibrahim M. E., Gooda A. A., Zoorob H. H. (2018). J. Heterocycl. Chem..

[cit36] Mashhadinezhad M., Mamaghani M., Rassa M., Shirini F. (2019). ChemistrySelect.

[cit37] Monica G. L., Bono A., Alamia F., Lauria A., Martorana A. (2024). Bioorg. Med. Chem..

[cit38] Ghaith E. A., Zoorob H. H., Ibrahim M. E., Sawamura M., Hamama W. S. (2020). ChemistrySelect.

[cit39] Nadar S., Khan T. (2022). Chem. Biol. Drug Des..

[cit40] Hamama W. S., Hassanien A. E., El-Fedawy M. G., Zoorob H. H. (2017). J. Heterocycl. Chem..

[cit41] Guo X., Zhou Q., Fan X., Zhu Q., Jin M. (2024). ACS Appl. Polym. Mater..

[cit42] Olesiejuk M., Kudelko A., Świątkowski M. (2023). Dyes Pigm..

[cit43] Hamama W. S., Waly M. A., EL-Hawary I. I., Zoorob H. H. (2016). J. Heterocycl. Chem..

[cit44] Bozorgnia S., Pordel M., Davoodnia A., Beyramabadi S. A. (2024). Opt. Mater..

[cit45] Escolano M., Gaviña D., Alzuet-Piña G., Díaz-Oltra S., Sánchez-Roselló M., del Pozo C. (2024). Chem. Rev..

[cit46] Aly A. A., Abd El-Naby H. A., Ahmed E. Kh., Gedamy S. A., Alshammari M. B., Ahmad A., Bräse S. (2025). Curr. Org. Chem..

[cit47] Ajani O. O., Iyaye K. T., Ademosun O. T. (2022). RSC Adv..

[cit48] Rajendran S., Sivalingam K., Jayarampillai R. P. K., Wang W., Salas C. O. (2022). Chem. Biol. Drug Des..

[cit49] Hernández-Ayala L. F., Guzmán-López E. G., Galano A. (2023). Antioxidants.

[cit50] Kumar R., Thakur, Sachin A., Chandra D., Dhiman A. K., Verma P. K., Sharma U. (2024). Coord. Chem. Rev..

[cit51] Cai Q., Song H., Zhang Y., Zhu Z., Zhang J., Chen J. (2024). J. Agric. Food Chem..

[cit52] Owais M., Kumar A., Hasan S. M., Singh K., Azad I., Hussain A., Suvaiv, Akil M. (2024). Mini-Rev. Med. Chem..

[cit53] Ghosh S., Mallick S., Karolly D., Sarkar S. D. (2024). ACS Org. Inorg. Au.

[cit54] Al Zoubi W., Ko Y. G. (2020). J. Colloid Interface Sci..

[cit55] Abd El-Lateef H. M., Gaafar A. G. A., Alqahtani A. S., Al-Mutairi A. A., Alshaya D. S., Elsaid F. G., Fayadg E., Farouk N. A. (2024). RSC Adv..

[cit56] Jagtap P. A., Sawant V. R., Bhanage B. M. (2024). ChemCatChem.

[cit57] Sharma S., Singh K., Singh S. (2023). Curr. Org. Synth..

[cit58] Mohasin M., Alam M. Z., Ullah Q., Ahmad A., Rahaman P. F., Khan S. A. (2024). Polycycl.

[cit59] Dorababu A. (2021). Arch. Pharm..

[cit60] Mabire D., Coupa S., Adelinet C., Poncelet A., Simonnet Y., Venet M., Wouters R., Lesage A. S. J., Beijsterveldt L. V., Bischoff F. (2005). J. Med. Chem..

[cit61] Viveka T. L., Angajala G., Aruna V., Nakka M., Aparna Y. (2024). J. Mol. Struct..

[cit62] Al-Ostoot F. H., Zabiulla, Salah S., Khanum S. A. (2021). J. Iran. Chem. Soc..

[cit63] Bergwik J., Liu J., Padra M., Bhongir R. K. V., Tanner L., Xiang Y., Lundblad M., Egesten A., Adner M. (2024). Respir. Res..

[cit64] Wang P. Y., Chen H., Wanga Y., Lyu Y. K. (2020). J. Chem. Technol. Biotechnol..

[cit65] Al-Matarneh C. M., Nicolescu A., Marinas I. C., Găboreanu M. D., Shova S., Dascălu A., Silion M., Pinteală M. (2024). Molecules.

[cit66] Shumi G., Demissie T. B., Eswaramoorthy R., Bogale R. F., Kenasa G., Desalegn T. (2024). ACS Omega.

[cit67] Coa J. C., Yepes A., Carda M., Conesa-Milián L., Upegui Y., Robledo, Dr. S. M., Cardona-G W. (2020). ChemistrySelect.

[cit68] Bala I. A., Al Sharif O. F., Asiri A. M., El-Shishtawy R. M. (2024). Results Chem..

[cit69] El-Shershaby M. H., El-Gamal K. M., Bayoumi A. H., El-Adl K., Alswah M., Ahmed H. E. A., Al-Karmalamy A. A., Abulkhair H. S. (2021). New J. Chem..

[cit70] Kondaparla S., Soni A., Manhas A., Srivastava K., Purib S. K., Katti S. B. (2016). RSC Adv..

[cit71] Rezvanian A., Khodadadi B., Tafreshi S., Shiri P. (2024). Mol. Diversity.

[cit72] Sonawane A. D., Garud D. R., Udagawa T., Koketsu M. (2018). Org. Biomol. Chem..

[cit73] Belyaeva K. V., Nikitina L. P., Oparina L. A., Saliy V. S., Tomilin D. N., Kuzmin A. V., Afonin A. V., Trofimov B. A. (2024). New J. Chem..

[cit74] An X., Li N., Zhang L., Xu Z., Zhang S., Zhang Q. (2024). J. Hazard. Mater..

[cit75] http://www.africanplants.senckenberg.de/root/index.php?page_id=78&id=4191#

[cit76] Kaur R., Kumar K. (2021). Eur. J. Med. Chem..

[cit77] Yadav P., Shah K. (2021). Bioinorg. Chem..

[cit78] Talwar D., Gonzalez-de-Castro A., Li H. Y., Xiao J. (2015). Angew. Chem..

[cit79] Matada B. S., Pattanashettar R., Yernale N. G. (2021). Bioorg. Med. Chem..

[cit80] Arboleda A. D., Moreno L. M., Abonia R. (2024). Curr. Org. Chem..

[cit81] Kischkewitz M., Marinic B., Kratena N., Lai Y., Hepburn H. B., Dow M., Christensen K. E., Donohoe T. J. (2022). Angew. Chem., Int. Ed..

[cit82] Proisl k., Kafka S., Kosmrlj J. (2017). Curr. Org. Chem..

[cit83] Nazrullaev S. S., Bessonova I. A., Akhmedkhodzhaeva Kh. S. (2001). Chem. Nat. Compd..

[cit84] Anjanikar S. S., Chandole S. S. (2023). Orient. J. Chem..

[cit85] Barmade M. A., Agrawal P., Rajput S. R., Murumkar P. R., Rana B., Sahal D., Yadav M. R. (2024). RSC Med.Chem..

[cit86] Uddin A., Gupta S., Shoaib R., Aneja B., Irfan I., Gupta K., Rawat N., Combrinck J., Kumar B., Aleem M., Hasan P., Joshi M. C., Chhonker Y. S., Zahid M., Hussain A., Pandey K., Alajmi M. F., Murry D. J., Egan T. J., Singh S., Abid M. (2024). Eur. J. Med. Chem..

[cit87] Gao J., Hou H., Gao F. (2023). Eur. J. Med. Chem..

[cit88] Çi̇ftci̇ B., Ökten S., Koçyi̇ği̇t Ü. M., Atalay V. E., Ataş M., Çakmak O. (2024). Eur. J. Med. Chem. Rep..

[cit89] Guin S., Alden K. M., Krysan D. J., Meyers M. J. (2024). ACS Med. Chem. Lett..

[cit90] Verma S., Lal S., Narang R. (2024). Future Med. Chem..

[cit91] Arasakumar T., Mathusalini S., Gopalan S., Shyamsivappan S., Ata A., Mohan P. S. (2017). Bioorg. Med. Chem. Lett..

[cit92] El-Helw E. A. E., Asran M., Azab M. E., Helal M. H., Alzahrani A. Y. A., Ramadan S. K. (2024). Sci. Rep..

[cit93] Azimi S. G., Bagherzade G., Saberi M. R., Tehranizadeh Z. A. (2023). Bioinorg. Chem. Appl..

[cit94] Beus M., Persoons L., Daelemans D., Schols D., Savijoki K., Varmanen P., Yli-Kauhaluoma J., Pavić K., Zorc B. (2022). Mol. Diversity.

[cit95] Abdelmegeed H., Abdel Ghany L. M. A., Youssef A., El-Etrawycd A. S., Ryadc N. (2024). RSC Adv..

[cit96] He X., Chen J., Kandawa-Shultz M., Shao G., Wang Y. (2023). Dalton Trans..

[cit97] Jina G., Lib Z., Xiaoa F., Qia X., Sun X. (2020). Bioorg. Chem..

[cit98] Shashikumar N. D., Krishnamurthy G., Bhojyanaik H. S., Lokesh M. R., Jithendrakumara K. S. (2014). J. Chem. Sci..

[cit99] Pakhariya R. P., Bhatnagar A., Pemawat G. (2025). RSC Adv..

[cit100] Santos J. C. d., Alves J. E. F., de Azevedo R. D. S., de Lima M. L., de Oliveira Silva M. R., da Silva J. G., da Silva J. M., de Carvalho Correia A. C., do Carmo Alves de Lima M., de Oliveira J. F., de Moura R. O., de Almeida S. M. V. (2024). Int. J. Biol. Macromol..

[cit101] Saral A., Shahidha R., Thirunavukkarasu M., Muthu S. (2023). Chem. Phys. Impact..

[cit102] Wang S. B., Deng X. Q., Zheng Y., Zhang H. J., Quan Z. S. (2013). Arch. Pharmacal Res..

[cit103] Shabana K., Salahuddin, Mazumder A., Singh H., Kumar R., Tyagi S., Datt V., Sharma A. S., Yar M. S., Ahsan M. J., Yadav R. K. (2023). ChemistrySelect.

[cit104] Song J., Zhu Y., Zu W., Duan C., Xu J., Jiang F., Wang X., Li S., Liu C., Gaoa Q., Li H., Zhang Y., Tang W., Lu T., Chen Y. (2021). Bioorg. Med. Chem..

[cit105] Suthar S. K., Jaiswal V., Lohan S., Bansal S., Chaudhary A., Tiwari A., Alex A. T., Joesph A. (2013). Eur. J. Med. Chem..

[cit106] Pattanashetty S. H., Hosamani K. M., Barretto D. A. (2018). Chem. Data Collect..

[cit107] Krishna A., Vijayakumar V., Sarveswari S. (2020). ChemistrySelect.

[cit108] Kostopoulou I., Diassakou A., Kavetsou E., Kritsi E., Zoumpoulakis P., Pontiki E., Hadjipavlou-Litina D., Detsi A. (2021). Mol. Diversity.

[cit109] Azad M., Munawar M. A., Athar M. (2007). J. Appl. Sci..

[cit110] Rowley M., Leeson P. D., Stevenson G. I., Moseley A. M., Stansfield I., Sanderson I., Robinson L., Baker R., Kemp J. A., Marshall G. R., Foster A. C., Grimwood S., Tricklebank M. D., Saywell K. L. (1993). J. Med. Chem..

[cit111] Tunna T. S., Zaidul I. S. M., Ahmed Q. U., Ghafoor K., Al-Juhaimi F. Y., Uddin M. S., Hasan M., Ferdous S. (2015). S. Afr. J. Bot..

[cit112] Hassan M. M., Hassanin H. M. (2017). J. Heterocycl. Chem..

[cit113] Krokhalev V. M., Saloutin V. I., Romas A. D., Ershov B. A., Pashkevich K. I. (1990). Div. Chem. Sci..

[cit114] Detsi A., Bardakos V., Markopoulos J., Igglessi-Markopoulou O. (1996). J. Chem. Soc., Perkin Trans. 1.

[cit115] Abdou M. M., Seferoğlu Z., Fathy M., Akitsu T., Koketsu M., Kellow R., Amigues E. (2019). Res. Chem. Intermed..

[cit116] Athanasellis G., Gavrielatos E., Igglessi-Markopoulou O., Markopoulos J. (2003). J. Heterocycl. Chem..

[cit117] Abu-elwafa S. M., Mohamed E. E., Issa R. M., Gaber M. (1985). Indian J. Chem..

[cit118] Dessai P. G., Dessai S. P., Dabholkar R., Pednekar P., Naik S., Mamledesai S., Gopal M., Pavadai P., Kumar B. K., Murugesan S., Chandavarkar S., Theivendren P., Selvaraj K. (2023). Mol. Diversity.

[cit119] Bürgi H. B. and Dunitz J. D., Structure Correlation, John Wiley & Sons, 2008

[cit120] Ukrainets I. V., Tkach A. A., Yang L. Y. (2009). Chem. Heterocycl. Compd..

[cit121] Kouznetsov V. V., Méndez L. Y. V., Gómez C. M. M. (2005). Curr. Org. Chem..

[cit122] Nainwal L. M., Tasneem S., Akhtar W., Verma G., Khan M. F., Parvez S., Shaquiquzzaman M., Akhter M., Alam M. M. (2019). Eur. J. Med. Chem..

[cit123] Ramann G. A., Cowen B. J. (2016). Molecules.

[cit124] Prajapati S. M., Patel K. D., Vekariya R. H., Panchal S. N., Patel H. D. (2014). RSC Adv..

[cit125] Barluenga J., Rodriguez F., FaÇanas F. J. (2009). Chem.–Asian J..

[cit126] Weyesa A., Mulugeta E. (2020). RSC Adv..

[cit127] Bharate J. B., Vishwakarma R. A., Bharate S. B. (2015). RSC Adv..

[cit128] Teja C., Khan F. R. N. (2020). Chem.–Asian J..

[cit129] Vessally E., Edjlali L., Hosseinian A., Bekhradnia A., Esrafil M. D. (2016). RSC Adv..

[cit130] Kappe T., Aigner R., Hohengassner P., Stadlbauer W. (1994). J. Prakt. Chem..

[cit131] Toan D. N., Thanh N. D., Truong M. X., Van D. T. (2020). Arabian J. Chem..

[cit132] Razzaq T., Kappe C. O. (2007). Tetrahedron Lett..

[cit133] Toan D. N., Thanh N. D., Truong M. X., Bang D. N., Ngaa M. T., Huong N. T. T. (2020). New J. Chem..

[cit134] Abdel-Kadera D., Abass M., Shawkat S. (2024). Russ. J. Org. Chem..

[cit135] Stadlbauer W., Hojas G. (2004). J. Heterocyclic Chem..

[cit136] Bowman R. E., Campbell A., Tanner E. M. (1959). J. Chem. Soc..

[cit137] Ibrahim M. A., Hassanin H. M., Abass M., Badran S. (2013). Arkivoc.

[cit138] Tomita K. (1951). Yakugaku Zasshi.

[cit139] Elgogary S., Abd Elghafar H., Mashaly M. (2021). J. Chin. Chem. Soc..

[cit140] Hassan M. M., Othman E. S., Abass M. (2013). Res. Chem. Intermed..

[cit141] Alla K., Sarveswari S. (2019). Iran J. Sci. Technol. Trans. Sci..

[cit142] Sarveswari S., Vijayakumar V., Siva R., Priya R. (2015). Appl. Biochem. Biotechnol..

[cit143] Brawley J., Etter E., Heredia D., Intasiri A., Nennecker K., Smith J., Welcome B. M., Brizendine R. K., Gould T. W., Bell T. W., Cremo C. (2020). J. Med. Chem..

[cit144] Shariat M., Samsudin M. W., Zakaria Z. (2013). Chem. Cent. J..

[cit145] Mitsos C., Petrou J., Igglessi-Markopoulou O., Markopoulos J. (1999). J. Heterocycl. Chem..

[cit146] Roussaki M., Hall B., Lima S. C., da Silva A. C., Wilkinson S., Detsi A. (2013). Bioorg. Med. Chem. Lett..

[cit147] Baruah B., Dasu K., Vaitilingam B., Vanguri A., Casturi S. R., Yeleswarapu K. R. (2004). Bioorg. Med. Chem. Lett..

[cit148] Dutt S., Tyagi V. (2021). Tetrahedron Lett..

[cit149] Satheeshkumar R., Shankar R., Kaminsky W., Prasad K. J. R. (2016). ChemistrySelect.

[cit150] Zhang Q., Yuan J., Yu M., Zhang R., Liang Y., Huang P., Dong D. (2017). Synthesis.

[cit151] Hassanin H. M., Ibrahim M. A., Gabr Y. A., Alnamer Y. A. (2012). J. Heterocycl. Chem..

[cit152] Kumar D. K., Rajkumar R., Rajendran S. P. (2016). Chem. Heterocycl. Compd..

[cit153] Abass M., Alzandi A. R. A., Hassan M. M., Mohamed N. (2021). Polycyclic Aromat. Compd..

[cit154] Naik S. D., Chandavarkar S. K., Tawade S. S., Shingade S. G., Palkard M. B., Desai S. N. M. (2022). Indian J. Chem..

[cit155] Roschger P., Stadlbaue W. (1990). Liebigs Ann. Chem..

[cit156] Steinschifter W., Fiala W., Stadlbauer W. (1994). J. Heterocycl. Chem..

[cit157] Hojas G., Fiala W., Stadlbauer W. (2000). J. Heterocycl. Chem..

[cit158] Chimichi S., Boccalini M., Matteucci A. (2007). Tetrahedron.

[cit159] Raiturkara R. R., Desaia S. N. M., Graciasa V. M., Sangavkara A. D. S., Fernandesa C., Biradarb B. S., Chandavarkar S. K. (2022). Indian J. Chem..

[cit160] Reis M. G., Desai S. M., Naik S., Fernandes J., Tari P. (2016). Indian J. Chem..

[cit161] Salman G. A. (2018). Al-Mustansiriyah J. Sci..

[cit162] Desai S. N. M., Priolkar R. N. S., Karmali H. A. N., Amambe P. D., Birdar B. S. (2017). Int. J. Pharm. Pharm. Sci..

[cit163] Hassanin H. M., El-edfawy S. M. (2012). Heterocycles.

[cit164] Faber K., Kappe T. (1984). J. Heterocycl. Chem..

[cit165] Goswami S., Ghosh K., Mukherjee R., Adak A. K., Mahapatra A. K. (2001). J. Heterocycl. Chem..

[cit166] Nandeshwarappa B. P., Manjunatha S. K., Ramesh D. K., Suchitra M., Sadashiv S. O. (2020). J. Pharm. Sci..

[cit167] Girges M. M., Hanna M. A., Hassan H. M., Moawad E. B. (1988). Collect. Czechoslov. Chem. Commun..

[cit168] Bolakatti G., Palkar M., Katagi M., Hampannavar G., Karpoormathd R. V., Ninganagouda S., Badiger A. (2021). J. Mol. Struct..

[cit169] Vilsmeier A., Haack A. (1927). Ber. Dtsch. Chem. Ges..

[cit170] Ibrahim M. A., Hassanin H. M., Alnamer Y. A. (2014). Synth. Commun..

[cit171] Manaev A. V., Okhrimenko I. N., Lyssenko K. A., Traven V. F. (2008). Russ. Chem. Bull..

[cit172] kappe T., Aigner R., Jobstl M., Hohengassner P., Stadlbauer W. (1995). Heterocycl. Commun..

[cit173] Islamuddin M., Afzal O., Khan W. H., Hisamuddin M., Altamimi A. S. A., Husain I., Kato K., Alamri M. A., Parveen S. (2021). ACS Omega.

[cit174] Sankaran M., Kumarasamy C., Chokkalingam U., Mohan P. S. (2010). Bioorg. Med. Chem. Lett..

[cit175] Arasakumar T., Mathusalini S., Lakshmi K., Mohan P. S., Ata A., Lin C. H. (2016). Synth. Commun..

[cit176] Ibrahim S. S., El-Gendy Z. M., Allimony H. A., Othman E. S. (1999). Chem. Pap..

[cit177] Hassan M. M., Alzandi A. R. A., Hassan M. M. (2020). Arabian J. Chem..

[cit178] Abdel-Megid M., Abass M., Hassan M. (2007). J. Heterocycl. Chem..

[cit179] Hassan M. M., Abdel-Kariem S. M., Ali T. E. (2017). Phosphorus, Sulfur Silicon Relat. Elem..

[cit180] Sarveswari S., Vijayakumar V. (2016). Arabian J. Chem..

[cit181] Kalechits G. V., Ol’khovik V. K., Kalosha I. I., Skakovskii E. D., Pap A. A., Zenyuk A. A., Matveenko Y. V. (2001). Russ. J. Gen. Chem..

[cit182] Munawar M. A., Azad M., Siddiquia H. L., Nasim F. (2008). J. Chin. Chem. Soc..

[cit183] Bhupathi R. S., Devi B. R., Dubey P. K. (2012). Indian J. Chem..

[cit184] Maruthesh H., Katagi M. S., Nandeshwarappa B. P. (2023). Russ. J. Org. Chem..

[cit185] Hussein M. F., Ismail M. A., El-Adly R. A. (2016). Int. J. Org. Chem..

[cit186] Traven V. F., Manaev A. V., Voevodina I. V., Okhrimenko I. N. (2008). Russ. Chem. Bull..

[cit187] Krishna A., Sarveswari S. (2019). ChemistrySelect.

[cit188] Abass M., Abdel-Megid M., Hassan M. (2007). Synth. Commun..

[cit189] Abass M., Hassan A. (2003). Chem. Pap..

[cit190] Abass M., Mostafa B. B. (2005). Bioorg. Med. Chem..

[cit191] El-Taweel F. M. A. A. (2005). J. Heterocycl. Chem..

[cit192] Elnaggar N. N., Hamama W. S., Abd El Salam M., Ghaith E. A. (2025). RSC Adv..

[cit193] Abass M., Othman E. S. (2001). Synth. Commun..

[cit194] Hassan A., Badr M., Abdelhamid D., Hassan H. A., Abourehab M. A. S., Abuo-Rahma G. A. (2022). Bioinorg. Chem..

[cit195] Elagamey A. A., El-Taweel F. M. A., Khalil M. H. M. (2012). Sci. J. Damietta Fac. Sci..

[cit196] Rao V. S., Darbarwar M. (1989). Synthesis.

[cit197] Chen Z., Wang Z. (2016). Tetrahedron.

[cit198] Kappe T., Schnell B. (1996). J. Heterocycl. Chem..

[cit199] Fiala W., Stadlbauer W. (1993). J. Prakt. Chem..

[cit200] El-Sheref E. M., Elbastawesy M. A. I., Brown A. B., Shawky A. M., Gomaa H. A. M., Bräse S., Youssif B. G. M. (2021). Molecules.

[cit201] Madhu B., Reddy C. V. R., Dubey P. K. (2017). Synth. Commun..

[cit202] Madhu B., Sekar B. R., Reddy C. V. R., Dubey P. K. (2017). Res. Chem. Intermed..

[cit203] Bhat S. I., Trivedi D. R. (2014). RSC Adv..

[cit204] Bhupathi R., Madhu B., Devi B. R., Reddy C. V. R., Dubey P. K. (2016). J. Heterocyclic Chem..

[cit205] Barr S. A., Neville C. F., Grundon M. F., Boyd D. R., Malone J. F., Evans T. A. (1995). J. Chem. Soc., Perkin Trans. 1.

[cit206] Ye J. H., Ling K. Q., Zhang Y., Li N., Xu J. H. (1999). J. Chem. Soc., Perkin Trans. 1.

[cit207] Abass M., Allimony H. A., Hassan H. (2013). Phosphorus, Sulfur Silicon Relat. Elem..

[cit208] Morsy J. M., Hassanin H. M., Ismail M. M., Abd-Alrazk M. M. A. (2016). J. Chem. Res..

[cit209] Ali T., Wang H., Iqbal W., Bashir T., Shah R., Hu Y. (2023). Adv. Sci..

[cit210] Kappe T., Aigner R., Roschger P., Schnell B., Stadlbauer W. (1995). Tetrahedron.

[cit211] Luca Q. R., Fenwick A. Q. (2015). J. Photochem. Photobiol., B.

[cit212] Fonsecaa V., Chandavarkar S., Dabholkara R., Dessaia P. G., Deshpandec M., Desai S. N. M. (2021). Indian J. Chem..

[cit213] Azzman N., Anwar S., Mohamed W. A. S., Ahemad N. (2024). Med. Chem..

[cit214] Pervaiza A., Athara M. M., Khana M. A., Pervaizb M., Sagirc M., Naz M. Y. (2014). Sci. Int..

[cit215] Al-Majedy Y., Kadhum A. A., Ibraheem H., Al-Amiery A., Moneim A. A., Mohamad A. B. (2018). Sys. Rev. Pharm..

[cit216] Subhasrao B. A. , Master's thesis, Rajiv Gandhi University of Health Sciences, India, 2010

[cit217] Prousis K. C., Tzani A., Avlonitis N., Calogeropoulou T., Detsi A. (2013). J. Heterocycl. Chem..

[cit218] Ukrainets I. V., Bereznyakova N. L., Mospanova E. V. (2007). Chem. Heterocycle. Cmpd..

[cit219] Collin X., Robert J. M., Duflos M., Wielgosz G., Le Baut G., Robin-Dubigeon C., Grimaud N., Lang F., Petit J. Y. (2001). J. Pharm. Pharmacol..

[cit220] Zaman A., Ahmad I., Pervaiz M., Ahmad S., Kiran S., Khan M. A., Gulzar T., Kamal T. (2019). J. Mol. Struct..

[cit221] Vijayakumar V. (2016). Int. J. Chemtech Res..

[cit222] Elshaier Y. A. M. M., Aly A. A., Abd El-Aziz M., Fathy H. M., Brown A. B., Ramadan M. (2021). Mol. Diversity.

[cit223] Ukrainets I. V., Golik M. Y., Sidorenko L. V., Korniyenko V. I., Grinevich L. A., Sim G., Kryvanych O. V. (2018). Sci. Pharm..

[cit224] Yadav V., Reang J., Sharma V., Majeed J., Sharma P. C., Sharma K., Giri N., Kumar A., Tonk R. K. (2022). Chem. Biol. Drug Des..

[cit225] Priya N., Gupta A., Chand K., Singh P., Kathuria A., Raj H. G., Parmar V. S., Sharma S. K. (2010). Bioorg. Med. Chem..

[cit226] Shiro T., Fukaya T., Tobe M. (2015). Eur. J. Med. Chem..

[cit227] Kwak S. H., Shin S., Lee J. H., Shim J. K., Kim M., Lee S. D., Lee A., Bae J., Park J. H., Abdelrahman A., Müller C. E., Cho S. K., Kang S. G., Bae M. A., Yang J. Y., Ko H., Goddard III W. A., Kim Y. C. (2018). Eur. J. Med. Chem..

[cit228] Senerovic L., Opsenica D., Moric I., Aleksic I., Spasić M., Vasiljevic B. (2020). Adv. Exp. Med Bio..

[cit229] Aly A. A., El-Sheref E. M., Mourad A. E., Bakheet M. E. M., Bräsem S. (2020). Mol. Diversity.

[cit230] Arya K., Agarwal M. (2007). Bioorg. Med. Chem. Lett..

[cit231] Katagi M. S., Bolakatti G. S., Badiger A. M., Satyanarayana D., Mamledesai S. N., Sujatha M. L. (2013). Drug Res..

[cit232] Sharma V., Das R., Mehta D. K., Sharma D., Aman S., Khan M. U. (2024). Mol. Diversity.

[cit233] Quan Z. S., Wang J. M., Rho J. R., Kwak K. C., Kang H. C., Jun C. S., Chai K. Y. (2005). Bull. Korean Chem. Soc..

[cit234] Pradhanand A., Vishwakarma S. K. (2020). Chem. Int..

[cit235] Smita S., Anand G., Ranjit S., Vikrant V. (2011). Int. J. Pharm. Sci. Res..

[cit236] Katagi M. S., Fernandes J., Satyanarayana D., Bolakatti G., Mamledesai S. N. (2014). J. Pharm. Sci..

[cit237] Katagi S. M., Fernandes J., Mamledesai S., Satyanarayana D., Dabadi P., Bolakatti G. (2015). J. Pharm. Res..

[cit238] Bi H., Ouyang Q., Wei Z., Zheng Z. (2020). Bioinorg. Chem..

[cit239] Katagi M. S., Mamledesai S., Bolakatti G., Fernandes J., ML S., Tari P. (2020). Chem. Data Collect..

[cit240] Toan D. N., Thanh N. D., Truong M. X., Van D. T. (2022). Med.Chem..

[cit241] Li Q., Woods K. W., Wang W., Lin N. H., Claiborne A., Gu W., Cohen J., Stoll V. S., Hutchins C., Frost D., Rosenberg S. H., Sham H. L. (2005). Bioorg. Med. Chem. Lett..

[cit242] Detsi A., Bouloumbasi D., Prousis K. C., Koufaki M., Athanasellis G., Melagraki G., Afantitis A., Igglessi-Markopoulou O., Kontogiorgis C., Hadjipavlou-Litina D. J. (2007). J. Med. Chem..

[cit243] Ginsburg A. S., Grosset J. H., Bisha W. R. (2003). Lancet Infect. Dis..

[cit244] Aubry A., Veziris N., Cambau E., Truffot-Pernot C., Jarlier V., Fisher L. M. (2006). Antimicrob. *Agents Chemother*..

[cit245] Muscia G. C., Bollini M., Bruno A. M., Asís S. E. (2006). J. Chil. Chem. Soc..

[cit246] Cheng A. F. B., Yew W. W., Chan E. W. C., Chin M. L., Hui M. M. M., Chan R. C. Y. (2004). Antimicrob. Agents Chemother..

[cit247] Dine I., Mulugeta E., Melaku Y., Belete M. (2023). RSC Adv..

[cit248] Yin H., Wu Y., Gu X., Feng Z., Wang M., Feng D., Wang M., Chenga Z., Wang S. (2022). RSC Adv..

[cit249] Elanany M. A., Osman E. E. A., Gedawy E. M., Abou-Seri S. M. (2023). Sci. Rep..

[cit250] Thurner F., Alatraktchi F. A. (2023). Chemosensors.

[cit251] Ji C., Sharma I., Pratihar D., Hudson L. L., Maura D., Guney T., Rahme L. G., Pesci E. C., Coleman J. P., Tan D. S. (2016). ACS Chem. Biol..

[cit252] Alla K., Vijayakumar V., Sarveswari S. (2023). Polycycl.

[cit253] Khasnobis S., Escuyer V. E., Chatterjee D. (2002). Expert. Opin. Ther. Targets.

[cit254] Ahsan M. J., Ansari M. Y., Yasmin S., Jadav S. S., Kumar P., Garg S. K., Aseri A., Khalilullah H. (2015). Infect. Disord. Drug Targets.

[cit255] Minovski N., Vracko M., Šolmajer T. (2011). Mol. Diversity.

[cit256] Lilienkampf A., Mao J., Wan B., Wang Y., Franzblau S. G., Kozikowski A. P. (2009). J. Med. Chem..

[cit257] Villemagne B., Crauste C., Flipo M., Baulard A. R., Déprez B., Willand N. (2012). Eur. J. Med. Chem..

[cit258] Reddy D. S., Hosamani K. M., Devarajegowda H. C. (2015). Eur. J. Med. Chem..

[cit259] Sharma M. K., Kumawat M. K., Diwan A., Sardana S., Yadav N., Kumar B. (2024). Herb. Med. J..

[cit260] Andriole V. T. (1994). Infect. Dis. Clin. Pract..

[cit261] Dube P. S., Legoabe L. J., Beteck R. M. (2023). Mol. Diversity.

[cit262] Appelbaum P. C., Hunter P. A. (2000). Int. J. Antimicrob. Agents.

[cit263] Pham T. D. M., Ziora Z. M., Blaskovich M. A. T. (2019). Med. Chem. Commun..

[cit264] Emmerson A. M., Jones A. M. (2003). J. Antimicrob. Chemother..

[cit265] Stahlmann R. (2002). Toxicol. Lett..

[cit266] Wagenlehner F. M. E., Naber K. G. (2005). Curr. Infect. Dis. Rep..

[cit267] Pommier Y., Leo E., Zhang H. L., Marchand C. (2010). Chem. Biol..

[cit268] Kumar, A. A., Fernandes J., Kumar P. (2014). Orient. J. Chem..

[cit269] Krishnakumar V., Khan F. R. N., Mandal B. K., Mitta S., Dhasamandha R., Govindan V. N. (2012). Res. Chem. Intermed..

[cit270] Kumar C. P., Katagi M. S., Nandeshwarappa B. P. (2022). Chem. Data Collect..

[cit271] Al-Issa S. A. (2013). Saudi Pharm. J..

[cit272] Butler M. M., LaMarr W. A., Foster K. A., Barnes M. H., Skow D. J., Lyden P. T., Kustigian L. M., Zhi C., Brown N. C., Wright G. E., Bowlin T. L. (2007). Antimicrob. Agents Chemother..

[cit273] Azaba H. A., Ibrahim I. A., Hassan N., Abbas A. M., Darwish H. M. (2017). J. Lumin..

[cit274] An R., Ahmed M., Li H., Wang Y., Zhang A., Bi Y., Yu Z. (2021). Sci. Rep..

[cit275] Liu Z. L., Chu S. S., Jiang G. H. (2009). J. Agric. Food Chem..

[cit276] Liu Y. X., Zhao H. P., Wang Z. W., Li Y. H., Song H. B., Riches H., Beattie D., Gu Y. C., Wang Q. M. (2013). Mol. Diversity.

[cit277] Jampilek J., Musiol R., Pesko M., Kralova K., Vejsova M., Carroll J., Coffey A., Finster J., Tabak D., Niedbala H., Kozik V., Polanski J., Csollei J., Dohnal J. (2009). Molecules.

[cit278] Abe M., Imai T., Ishii N., Usui M. (2006). Biosci., Biotechnol., Biochem..

[cit279] Uivarosi V. (2013). Molecules.

[cit280] Khalaf M. M., Gouda M., Shalabi K., Shaaban S., Abd El-Lateef H. M. (2024). ACS Omega.

[cit281] Althobiti H. A., Zabin S. A. (2020). Open Chem..

[cit282] Fouad R., El-Shafiy H. F. (2019). J. Mol. Struct..

[cit283] Creaven B. S., Duff B., Egan D. A., Kavanagh K., Rosair G., Thangella V. R., Walsh M. (2010). Inorg. Chim. Acta.

[cit284] Habibi K., Mamaghani M., Nikpassand M. (2018). Bulg. Chem. Commun..

[cit285] Azad M. (2007). J. Appl. Sci..

[cit286] Ayyannan G., Karthikeyan K., Vivekananthan S. S., Gopiraman M., Rathinavelu A. (2013). Ionics.

[cit287] Fernandes C. M., Costa A. R., Leite M. C., Martins V., Lee H. S., da CS Boechat F., de Souza M. C. B. V., Batalha P. N., Lgaz H., Ponzio E. A. (2023). J. Mol. Liq..

[cit288] Abdou M. M., EL-Haddad M. N. (2025). J. Mol. Struct..

[cit289] Meng X. M. , Wang S. X. and Zhu M. Z., Quinoline-Based Fluorescence Sensors, Molecular Photochemistry - Various Aspects, 2012, ch. 2, 10.5772/31771

[cit290] Bag B., Pal A. (2011). Org. Biomol. Chem..

[cit291] Grabchev I., Chovelon J. M., Petkov C. (2008). Spectrochim. Acta, Part A.

[cit292] Zhang L., Fan J., Peng X. (2009). Spectrochim. Acta, Part A.

[cit293] Diwan U., Kumar A., Kumar V., Upadhyay K. K. (2013). Dalton Trans..

[cit294] Yang Y., Yu K., Yang L., Liu J., Li K., Luo S. (2014). Sensors.

[cit295] Li B., Tian J., Zhang D., Tian F. (2017). Lumin..

